# Unilateral magnetic resonance-guided focused ultrasound for medication-refractory essential tremor: 5-year continued access study

**DOI:** 10.3389/fneur.2025.1659203

**Published:** 2025-10-22

**Authors:** Pejman Ghanouni, Vibhor Krishna, Howard M. Eisenberg, W. Jeffrey Elias, G. Rees Cosgrove, Ryder Gwinn, Michael G. Kaplitt, Gordon H. Baltuch

**Affiliations:** ^1^Department of Radiology, Stanford University, Stanford, CA, United States; ^2^Department of Neurosurgery, University of North Carolina, Chapel Hill, NC, United States; ^3^Department of Neurosurgery, University of Maryland, Baltimore, MD, United States; ^4^Department of Neurosurgery, University of Virginia, Charlottesville, VA, United States; ^5^Department of Neurosurgery, Brigham and Women's Hospital, Harvard Medical School, Boston, MA, United States; ^6^Evergreen Health Neurosurgery, Kirkland, WA, United States; ^7^Department of Neurological Surgery, Weill Cornell Medicine, New York, NY, United States; ^8^Department of Neurosurgery, Columbia University, New York, NY, United States

**Keywords:** essential tremor (ET), magnetic resonance-guided focused ultrasound (MRgFUS), unilateral MRgFUS, thalamotomy, medication-refractory ET

## Abstract

**Background:**

Essential tremor (ET) is a common neurologic disorder, with 30–50% of patients experiencing medication-refractory symptoms. Magnetic resonance-guided focused ultrasound (MRgFUS) thalamotomy is an approved, effective treatment for medication-refractory ET. In this open-label, continued access study, subjects were enrolled prospectively after the pivotal MRgFUS trial completed enrollment, but before US Food and Drug Administration approval. The objective of this study was to evaluate the long-term (5-year) effectiveness and safety of unilateral MRgFUS thalamotomy in medication-refractory ET patients.

**Methods:**

Effectiveness was evaluated by change from baseline in Clinical Rating Scale for Tremor (CRST) scores and quality of life (QoL) with the Quality of life in Essential Tremor (QUEST) questionnaire. Adverse events (AEs) following MRgFUS thalamotomy were recorded. Observed data were utilized for the main analysis. Sensitivity analyses using last observation carried forward and best-worst case scenarios were completed to evaluate the impact of missing data at long-term visits.

**Results:**

Of 61 treated subjects, the mean (SD) age was 69.5 (14.0) years, most (67.2%) were male, and 26 (42.6%) were observed for 5 years. MRgFUS thalamotomy improved tremor/motor function (CRST Parts A and B), tremor severity (postural component of CRST Part A for the treated side), and functional disability (CRST Part C) scores throughout the study. At 1- and 5-year follow-up, respective percentage improvements from baseline were: tremor/motor function, 62.2% and 51.9%; tremor severity, 75.6% and 67.4%; and functional disability, 65.4 and 35.4%. QoL improved by 53.6 and 43.7% at 1- and 5-year follow-up, respectively. Almost all related AEs were mild (85%) or moderate (12%) in severity, with 3% being severe. More than half of related events resolved in 6 months.

**Conclusion:**

MRgFUS thalamotomy is a safe and effective long-term treatment for patients with medication-refractory ET and is associated with improved QoL.

## Introduction

Essential tremor (ET) is one of the most common neurologic disorders. The primary feature of ET is kinetic tremor, which mainly affects the hands and arms, although other areas such as the head, voice and lower limbs may also be involved (1, 2). A meta-analysis of population-based epidemiological studies estimated the pooled prevalence of ET worldwide to be 1.3%, increasing to 5.8% for individuals aged ≥65 years (3). In the United States, approximately 7 million people, corresponding to about 2.2% of the US population, are estimated to experience ET (4).

First-line pharmacological treatment options for ET are propranolol and primidone (5). However, 30% to 50% of patients are either unresponsive to these agents or experience adverse side effects (6), and approximately 40% of patients discontinue medication within 2 years of prescription (7). Surgical interventions for ET patients refractory to medication include deep brain stimulation (DBS) and thalamotomy using stereotactic radiosurgery, radiofrequency ablation, or magnetic resonance-guided focused ultrasound (MRgFUS). These advanced techniques substantially reduce upper extremity tremor at 1 year and in the long term (8, 9).

In 2016, MRgFUS was approved by the US Food and Drug Administration (FDA) to treat medication-refractory ET (10). A randomized clinical trial found that MRgFUS thalamotomy significantly reduced upper extremity tremor and functional disability, and improved quality of life (QoL), in patients with ET compared with a sham procedure group at 12 months (11). Results at 2-year (12), 3-year (13), and 5-year (14) follow-up showed that tremor remained significantly improved, with associated improvements in disability and QoL, and without progressive or delayed complications.

In this open-label, continued access study, subjects were enrolled after the pivotal trial (11) completed enrollment, but before FDA approval. The objective of this study was to evaluate the long-term effectiveness and safety of MRgFUS in medication-refractory ET patients. Data on tremor reduction, disability, QoL and treatment-related adverse effects are presented.

## Methods

This was a multi-site, open-label, prospective, single-arm, interventional study investigating unilateral MRgFUS thalamotomy for the treatment of medication-refractory ET. This continued access study followed subjects for 5 years. Eight academic medical centers in the United States screened and enrolled subjects with medication-refractory ET between April 2015 and June 2017.

### Standard protocol approvals, registrations, and patient consents

The trial was registered prospectively at ClinicalTrials.gov (registration number: NCT02289560). The study was performed under investigational device exemption (FDA IDE G120246), and the protocol was approved by the FDA and the institutional review board of each participating center. All subjects provided written informed consent. The most recent signed copies of the Protocol and the Statistical Analysis Plan are available as Supplementary material.

### Inclusion and exclusion criteria

Eligibility criteria have been described previously (11). The main inclusion criteria were subjects aged ≥22 years with a diagnosis of medication-refractory ET and with a postural or intention tremor severity (Clinical Rating Scale for Tremor [CRST]) score (15) of ≥2 in the dominant hand/arm with significant disability due to ET (CRST score of ≥2 in any one of the items in subsection C for functional disability). Subjects with skull density ratio (a measure of the transparency of the skull to ultrasound) (16) less than 0.40 were excluded.

### Study procedure

Unilateral MRgFUS thalamotomy was conducted using high-resolution MRI and the ExAblate 4000 System (Insightec, Inc.) as described previously (17). Briefly, the ventral intermediate (Vim) nucleus of the thalamus was targeted based on stereotactic coordinates and tractography. Acoustic energy was increased gradually to raise temperature at the target, which was monitored using real-time MR thermometry. Clinical evaluations followed each sonication to evaluate safety and tremor response, and the target was moved until optimized. Thermal ablation was ultimately achieved, based on continual assessment of safety and tremor response throughout the procedure.

### Effectiveness assessments

Effectiveness was assessed using the CRST at baseline compared to follow-up visits at 1, 3, 6 months and annually from 1 to 5 years. The tremor/motor score was derived from the sum of CRST Part A (resting, postural and action components of tremor) for the treated upper extremity and CRST Part B (tasks of handwriting, drawing and pouring; scored 0–32 for the dominant side, 0–28 for the non-dominant). The tremor severity score was defined as the postural component of tremor (Part A, scored 0–4) for the treated upper extremity (contralateral to the thalamotomy). Similarly, action tremor represents the action component of tremor (Part A, scored 0–4). Functional disability was assessed by Part C of the CRST (scored 0–32). Higher scores indicate more severe tremor, motor dysfunction, or disability (15, 18). The primary effectiveness measure was a reduction in tremor/motor score (CRST A and B) of the treated upper extremity. The validated rating assessment of tremor was administered by a site-based movement disorder specialist. QoL was evaluated using the validated 30-item Quality of Life in Essential Tremor (QUEST) questionnaire (19, 20).

### Safety assessments

Adverse events (AEs) were recorded and assessed for causality and severity (mild, moderate, and severe; defined by the Common Terminology Criteria for Adverse Events (21) and relationship to the device or procedure. The Standard Code of Federal Regulation definitions was used in the assessment of serious or unanticipated AEs. A data safety monitoring board evaluated safety throughout the study and determined the relationship of all serious AEs. Related AEs were summarized by customized medical dictionary.

### Primary endpoints

The primary endpoint for effectiveness was evaluated using change in tremor/motor score (CRST A and B) from baseline to each follow-up. The primary endpoint for safety of MRgFUS thalamotomy was evaluated by the incidence and severity of device-/treatment-related complications from the treatment day through 5-years of follow-up.

### Secondary endpoints

Secondary endpoints were change in tremor severity (CRST Part A) from baseline, change in functional disability (CRST Part C) from baseline, and change in Quality of Life (QUEST) from baseline.

### Statistical analysis

For this long term follow up, no sample size or statistical analysis considerations were pre planned. Categorical variables were summarized by number (*n*) and percentage (%) and continuous variables by mean, standard deviation (SD) and 95% confidence intervals (CI). Primary and secondary endpoints were calculated based on observed data. All analyses were performed by modified Intention-To-Treat (mITT). To consider the impact of subjects lost to follow up, missing data were imputed using Last Observation Carried Forward (LOCF). Means and standard deviations were calculated for each subject in the observed and LOCF imputed datasets across visits and compared using Pearson's correlation coefficient (*r*) and Cohen's d effect size. In addition, an extreme case analysis, consisting of best-(100% improvement) and worst-case (no improvement) scenarios, was completed to assess sensitivity. Final observed outcomes of subjects who exited the study early were evaluated. Thresholds of 25 and 50% improvement in tremor/motor score were set to correspond with a substantial change in tremor amplitude (average 1-point change in each item of CRST A+B) (22) and “good” tremor outcomes as described previously (23, 24). Microsoft Excel for Microsoft 365 MSO (Version 2307 Build 16.0.16626.20198, 64-bit) was used to generate summary statistics using means, standard deviations, and confidence intervals. The study protocols and statistical analysis plan are available in eSAP 1, eSAP 2, and eSAP 3, respectively.

### Data availability

De-identified participant data will not be shared. The data are collectively covered by the research agreements with the enrolling sites and analysis is still ongoing. The study protocols (enrollment to 12 months; 1–5 year long-term follow-up) and statistical analysis plan are included.

## Results

**Figure 1:**
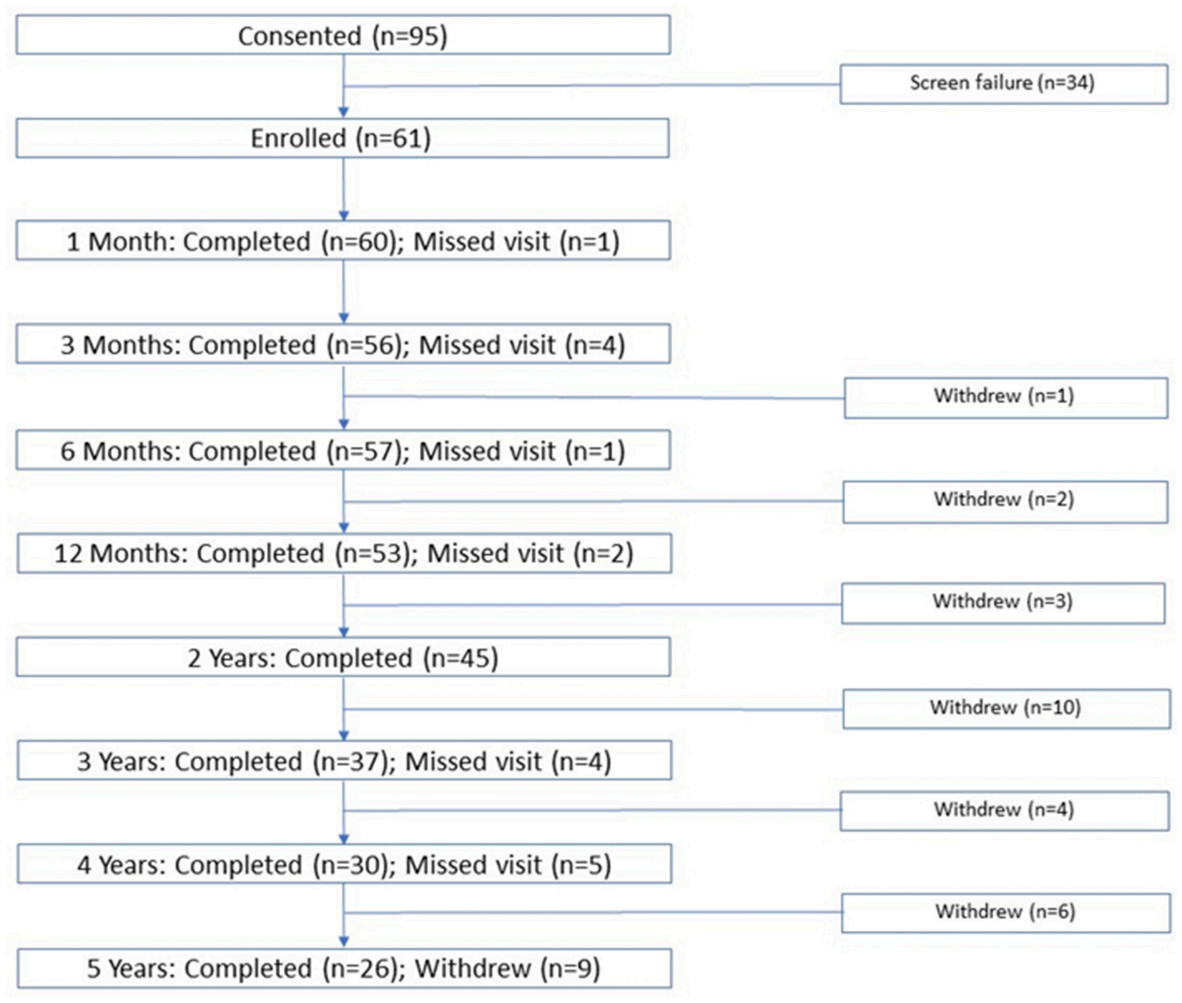
Patient disposition.

A total of 95 subjects provided informed consent, 34 of whom did not fulfill eligibility criteria (skull density ratio < 0.40, personal health/claustrophobia/anxiety). Subjects (*N* = 61) had a mean (SD) age of 69.5 (14.0) years and most (67.2%) were male. Most subjects were white (93.4%), 4.9% were Asian, and 1.6% were American-Indian/Eskimo (Table 1). Subject disposition, showing the number of subjects attending each follow-up visit (1, 3, and 6 months and 1, 2, 3, 4, 5 years) is shown in Figure 1. Of 61 treated subjects, 57 subjects (93%) were observed at 6 months, 53 (87%) at 12 months, and 26 subjects (42.6%) were followed for the full 5 years. Of the 35 subjects who did not complete the 5 years of long-term follow-up, 20 withdrew for reasons unrelated to the study, including restrictions imposed by the COVID-19 pandemic, commonly during long-term follow up (years 2–5). Six subjects were lost to follow-up and 4 received alternative treatment (DBS on ipsilateral or contralateral side, second MRgFUS procedure). Three patients died of unrelated causes and two withdrew due to dissatisfaction with treatment outcomes. Due to missing data, the number of subjects with observed data for each outcome does not necessarily match the total number of subjects shown in Figure 1.

**Table 1:** Patient demographics.

**Parameter**	**Value**
Age (years), mean (SD)	69.5 (14.0)
Sex, *n* (%)	
Male	41 (67.2)
Female	20 (32.8)
Race, *n* (%)	
White	57 (93.4)
Asian	3 (4.9)
American Indian-Eskimo	1 (1.6)
Family history of ET, *n* (%)	45 (74)
Time since ET diagnosis (years), mean	19.5
Skull Density Ratio, mean	0.54
Dominant Hand, *n* (%)	
Right	47 (77)
Left	14 (23)
Treated Side of the Brain, *n* (%)	
Right	16 (26)
Left	45 (74)
Prior pharmacological treatment	61 (100)
Prior surgical treatment	0 (0)
Prior botulinum toxin injections	[n] (1.6)

### Effectiveness

**Figure 2:**
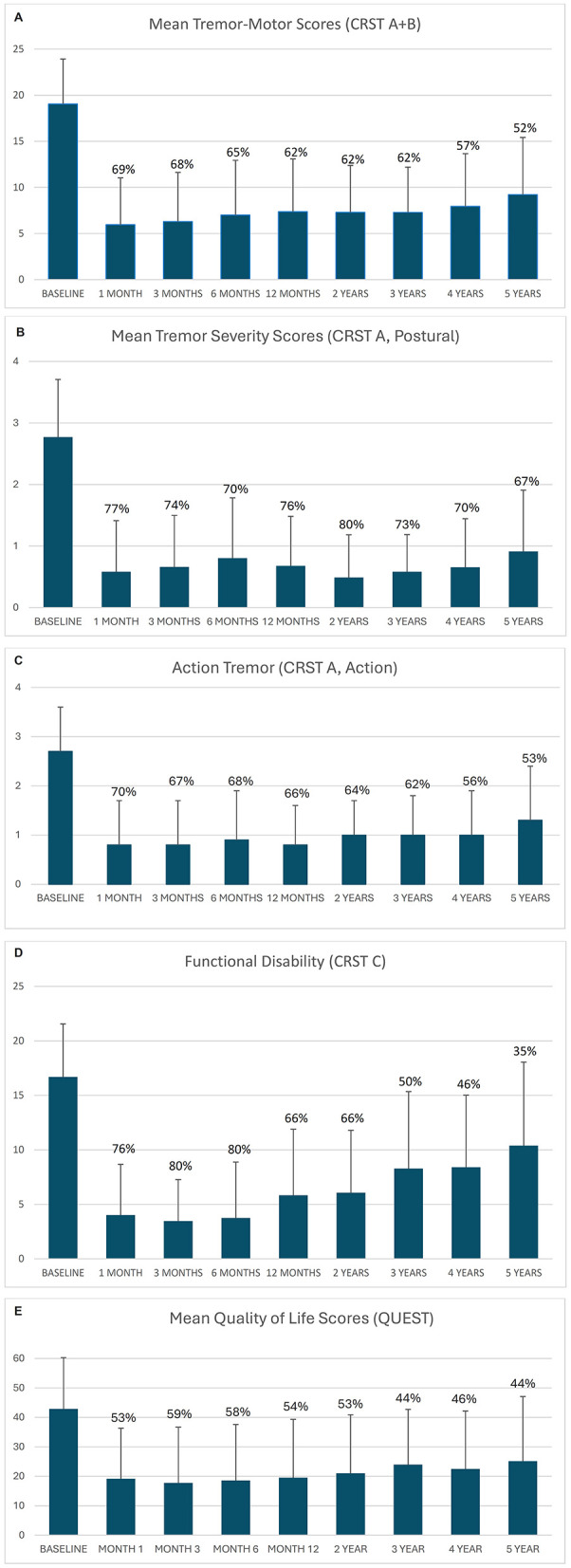
**(A)** Mean tremor/motor score (CRST Part A and B). **(B)** Mean tremor severity scores (CRST Part A, Postural). **(C)** Mean action tremor scores (CRST Part A, Action). **(D)** Mean functional disability scores (CRST Part C). **(E)** Mean quality of life scores (QUEST). Error bars represent standard deviation. Data labels represent % reduction from baseline.

Measures of effectiveness (CRST domain scores, mean and standard deviation and percentage change from baseline) are shown in Figure 2 and Supplementary Table 1 and at all observed time points. Improvement in CRST Part A and B (tremor/motor), CRST Part A (postural tremor in treated upper extremity), and CRST Part C (functional disability) scores from baseline were observed throughout the study. At 1-, 2-, 3-, 4- and 5-year follow-up, percentage reductions in tremor/motor (CRST Part A and B) scores from baseline were 62.2%, 62.4%, 61.8%, 57.6%, and 51.9%, respectively (Figure 2a). Tremor severity (CRST Part A) scores were reduced by 75.6%, 80.0%, 73.1%, 70.6%, and 67.4%, respectively (Figure 2b). Action tremor scores were reduced by 65.7%, 64.1%, 62.3%, 56.0%, and 53.2%, respectively (Figure 2c). Respective reductions in functional disability (CRST Part C) scores were 65.4%, 66.2%, 50.4%, 45.7% and 35.4% (Figure 2d).

**Figure 3:**
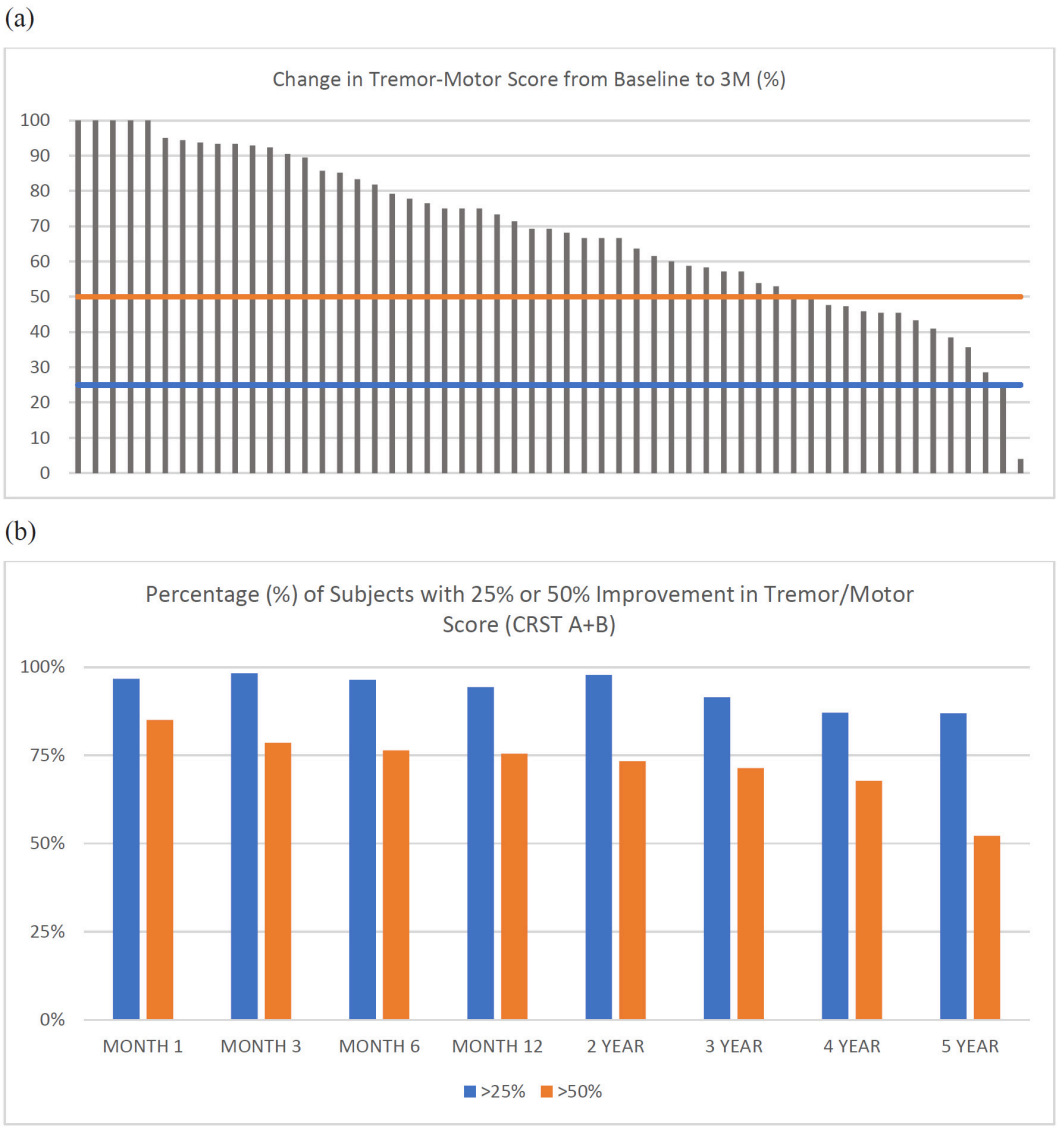
**(a)** Individual tremor/motor scores (CRST A+B) for each subject (*n* = 55) at 3-months. Horizontal lines represent a reduction (improvement) in tremor/motor score of 25% (blue) or 50% (orange). **(b)** Percentage (%) of subjects with greater than 25% or 50% improvement in tremor/motor score (CRST A+B) at each timepoint.

Improvement in individual tremor/motor scores ranged from 4% to 100% at 3 months (median 69.2%; Figure 3a). The percentage of subjects with an improvement of 25 and 50% in tremor/motor scores, respectively, at each study visit were: 96.7 and 85% at month 1 (*n* = 60), 98.2 and 78.6% at 3 months (*n* = 56), 96.4 and 76.4% at month 6 (*n* = 56), 94.3 and 75.5% at month 12 (*n* = 53), 97.8 and 73.3% at year 2 (*n* = 45), 91.4 and 71.4% at year 3 (*n* = 35), 87.1 and 67.7% at year 4 (*n* = 31), and 87 and 52.2% at year 5 (*n* = 23) (Figure 3b).

Improvements in tremor, motor function, and disability were accompanied by improvements in QoL, as shown by reduced overall QUEST scores (Supplementary Table 2, Figure 2e). At 1-, 2-, 3-, 4- and 5-year follow-up, percentage improvements from baseline in QUEST scores were 53.6% (95% CI 41.8–65.5), 52.6% (95% CI 42.4–62.8), 43.6% (95% CI 31.8–55.4), 45.5% (95% CI 30.5–60.6), and 43.7% (95% CI 27.1–60.4), respectively.

**Figure 4:**
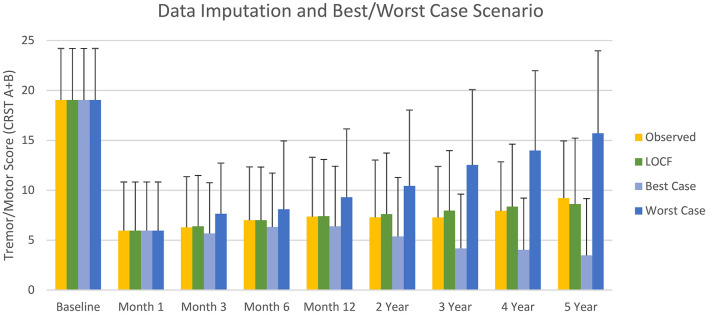
Mean (SD) observed tremor/motor scores, and scores imputed by last observation carried forward (LOCF) and for best- and worse-case scenarios (sensitivity analysis) during the study.

Mean imputed LOCF and observed CRST A+B data were similar (Figure 4), with Pearson's coefficient r >0.99. Based on Cohen's d < 0.2, the effect size was small. Overall, the mean (SD) imputed LOCF CRST A+B was 7.4 (6.1) at 1-year follow-up compared with an observed value of 7.4 (5.7). The respective mean (SD) of best- and worst-case scenarios for CRST A+B, were 5.4 (5.9) and 9.3 (7.6) at 1 year follow-up; 4.2 (5.2) and 12.5 (8.0) at year 3; and 3.5 (5.8) and 15.7 (7.3) at year 5 (Figure 4). The wide interval between best- and worst-case scenarios at long-term follow-up highlights the implausibility of either scenario but does fully account for all uncertainty due to missing data.

In an exploratory *post-hoc* analysis that compared the observed data for the primary efficacy endpoint (tremor-motor scores) at each timepoint to the corresponding group at baseline using a paired samples *t*-test, the difference in samples at each timepoint was significant (*p* < 0.0005) [data not reported], although a large number of subjects were lost to long-term follow-up.

At baseline, the subjects who completed all study visits (*n* = 23) had similar tremor-motor scores compared to the subjects who dropped out (*n* = 38) (18.2 + 4.6 for completers; 19.6 + 5.0 for dropouts). Of the subjects who dropped out of the study, the majority (73.7%; 28 of 38) had an improvement of 50% or greater in tremor/motor score at the last visit.

### Safety

In this study, 121 related AEs were reported in 52 subjects. All related AEs started within 30 days and no new related events were reported after 30 days. Most AEs (103/121, 85.1%) were mild, some (14/121, 11.6%) were moderate, and four (3.3%; 2 imbalance, 1 ataxia, 1 dysmetria) were severe. The most common related AEs were numbness/paresthesia (21 of 61 subjects, 34.4%) and imbalance (23 of 61 subjects, 37.7%). By 6 months, more than half (73/121, 60.3%) of all related AEs resolved. Numbness/tingling (13 of 57 subjects, 22.8%) and imbalance (12 of 57 subjects, 21.1%) were the most common ongoing related events reported at 6 months. At 5 years, 19 related events were ongoing in 14 subjects. Thirteen were mild in 9 subjects, 4 were moderate in 4 subjects, and 2 were severe in 1 subject.

Table 2 shows the reporting of related AEs where the incidence is greater than 2 subjects per event. The later visit columns show the ongoing AEs that had not yet resolved at each time point. Numbness/tingling (4/26 subjects, 15.4%) was the most common ongoing AE reported at 5 years.

**Table 2:** Number of subjects reporting related adverse events (AEs) following MR-guided focused ultrasound (MRgFUS) thalamotomy.

**Adverse event**	**Total (*n* = 61)**	**1 Month (*n =* 60)**	**3 Months (*n =* 56)**	**6 Months (*n =* 57)**	**12 Months (*n =* 53)**	**2 Year (*n =* 45)**	**3 Year (*n =* 37)**	**4 Year (*n =* 30)**	**5 Year (*n =* 26)**
Imbalance	23 (37.7)	19	12	12 (21.1)	11	9	8	6	3 (11.5)
Numbness/tingling	21 (34.4)	18	15	13 (22.8)	9	8	7	5	4 (15.4)
Ataxia	10 (16.4)	10	6	6 (10.5)	4	4	1	1	1 (3.8)
Dysmetria	9 (14.8)	6	6	6 (10.5)	6	6	4	3	3 (11.5)
Gait disturbance	9 (14.8)	8	6	5 (8.8)	5	5	2	2	1 (3.8)
Dysarthria	7 (11.5)	7	3	2 (3.5)	1	1	1	0	0 (0.0)
Dysgeusia	6 (9.8)	5	4	3 (5.3)	2	2	2	2	2 (7.7)
Headache	6 (9.8)	4	1	1 (1.8)	0	0	0	0	0
Fatigue	5 (8.2)	4	3	2 (3.5)	2	2	1	1	1 (3.8)

AEs at 12 months and 5 years represent subjects experiencing ongoing events, as no new related events were reported during 5-year follow-up. Number of subjects experiencing AEs are displayed as n. Percentage of subjects is shown at selected timepoints (%).

Less commonly reported AEs occurring in 2 or fewer subjects (< 3.3% of subjects) include dysgnathia, nausea/vomiting, vision problem, edema, muscle weakness, IV site infection, jaw pain, lethargy, myoclonus, slurred speech, and unclear thinking.

## Discussion

This open-label, prospective, interventional study investigated the long-term effectiveness and safety of unilateral MRgFUS for the treatment of medication-refractory ET. Effectiveness was demonstrated at 5 years by a clinical improvement from baseline in mean (SD) tremor/motor score [9.2 (6.2) vs. 19.1 (4.9), 51.9% reduction], tremor severity [0.9 (1.0) vs. 2.8 (0.9), 67.4% reduction], action tremor [1.3 (1.1) vs. 2.7 (0.9), 53.2% reduction], and functional disability [10.4 (7.7) vs. 16.7 (4.9), 35.4% reduction], which remained improved through 5 years. Although functional disability scores remained improved, they did gradually decline over time. As functional disability is a comprehensive measure that is less directly related to tremor control, this gradual decline may be expected when observing an elderly population with ET over 5 years. In addition, progressive worsening of QoL in this elderly, aging population may be expected, as QoL incorporates multiple domains, including physical, psychosocial, communication, work and finances, and hobbies and leisure.

The study followed subjects for 5 years, but less than half of the cohort (42.6%) completed the 5-year follow-up visit. Missing long-term data is common in longitudinal studies of elderly cohorts with comorbidities (25). We attempted to address this by imputing data by LOCF, which gave very similar results when compared to the observed data. We also provided a range of potential, though improbable, outcomes, based on best- and worst-case scenarios. It is probable, based on published data of the long-term (5-year) durability of tremor relief following MRgFUS (14), that the imputed results realistically reflect the missing data. Finally, the majority (73.7%) of subjects who discontinued in the study had good outcomes (tremor/motor score with 50% improvement or more at last visit), suggesting that subjects did not drop out due to bad outcomes.

In the current study, changes in tremor reduction, assessed by CRST scores, were accompanied by a clinically meaningful improvement in QoL, similar to those reported by Cosgrove et al. (14); QUEST scores exceeded the minimal clinically important difference (MCID; −4.47) (26) for improvement throughout the study. Results from the current multicenter study align with those reported in other long-term studies of MRgFUS for ET, adding to the existing body of literature regarding the long-term efficacy and safety of MRgFUS (27, 28). Based on a 5-year single-center experience in 44 patients with ET, Sinai et al. reported that MRgFUS thalamotomy was an effective and safe procedure that provided long-term tremor relief and improvement in QoL, even in patients with medication-resistant disabling tremor (27). In another long-term single-center study which followed ET patients treated with MRgFUS thalamotomy for up to 8 years (mean follow-up time 3 years), approximately two-thirds of patients reported improvement in hand tremor at last follow-up and 73% reported meaningful change in their overall condition post-procedure. In addition, most (89%) affirmed their treatment decision in retrospect (28). Meta-analyses found that MRgFUS results in significantly improved tremor outcomes and QoL in patients with ET (29, 30).

Most related AEs were transient (60.3%), and mild (85.1%). By definition, mild events do not interfere with activities of daily living or require therapy. No new AEs were reported after Year 1 follow-up and no persistent serious events were reported at 5 years. Numbness/paresthesia and imbalance were the most common AEs reported, similar to the pivotal clinical trial (11). Since roughly half the cohort observed at 12 months were not observed at 5 years, it is possible that the long-term rate of AEs may be higher. Therefore, while the rate within the population studied for most AEs reduced at 5 years compared to 1 year, we have no way to account for censored, ongoing events in those subjects lost to follow up.

Thalamic DBS is a commonly used surgical approach for ET and represents a suitable option for bilateral treatment (9). DBS can be adjusted to optimize efficacy and minimize AEs, but because of its disadvantages, including the need for ongoing stimulation adjustments, device-related issues, and, although uncommon, surgical risks of intracranial bleeding and infection, it may be rejected by patients (31, 32). Long-term complications of DBS include loss of benefit due to tolerance, habituation, or device malfunction (lead fracture, pulse generator failure) and other factors including device infection or erosion, intermittent stimulation and pain or discomfort (33). Moreover, although MRgFUS is a relatively new treatment option, tremor outcomes and safety profiles have improved since the pivotal study (34).

The study has several limitations. Noting that this was a continued access study, the single-arm, open-label design, which lacked a comparator group and blinding of participants or assessors, limits the ability to draw definitive causal inferences. Furthermore, enrollment during 2015–2017 may have affected study outcomes due to earlier operator experience, evolving protocols, and less mature MRgFUS technology, potentially influencing both effectiveness and safety profiles. The patient's perspective is important in ET; although QoL was assessed in the current study, subjective scales to assess patients' perspectives (e.g., Patient Global Impression) were not employed. In addition, the potential influence of lesion placement or SDR on long-term outcomes was not assessed. In common with the pivotal clinical trial of MRgFUS with 5-year follow-up in patients with ET (11–14), the lack of inclusion of black or Hispanic subjects represents a limitation of our study. With additional data being generated from more widespread use of MRgFUS, more generalizable data may soon be available to address these limitations.

In conclusion, unilateral MRgFUS thalamotomy is an effective long-term treatment for patients with medication-refractory ET and is associated with improved QoL. AEs were often transient and mainly mild in severity, and no new safety signals were observed after 1-year follow-up.

## Data Availability

The original contributions presented in the study are included in the article/Supplementary material, further inquiries can be directed to the corresponding author.
