# Supplementary material for: Unilateral magnetic resonance-guided focused ultrasound for medication-refractory essential tremor: 5-year continued access study
Source: Front Neurol. 2025 Oct 22;16:1659203. doi: 10.3389/fneur.2025.1659203 (PMC12587677; doi:10.3389/fneur.2025.1659203)
Supplement: Supplementary file 1 [file Table_1.docx]

**Supplementary Table 1 Effectiveness: CRST scores and percentage (%) change from baseline during 5-year follow-up.** Composite CRST Parts A+B represents tremor/motor function, CRST Part A postural scores assess tremor severity, and CRST Part C represents functional disability.

|  | **Baseline** | **Follow-up** | | | | | | | |
| --- | --- | --- | --- | --- | --- | --- | --- | --- | --- |
|  |  | **1 Month** | **3 Months** | **6 Months** | **1 Year** | **2 Years** | **3 Years** | **4 Years** | **5 Years** |
| Composite CRST Parts A + B (tremor/motor function) scores and percentage change from baseline | | | | | | | | | |
| n | 61 | 60 | 55 | 55 | 53 | 45 | 35 | 31 | 23 |
| Scores, mean (SD) | 19.05 (4.88) | 5.95 (5.08) | 6.29 (5.34) | 7.00 (5.94) | 7.36 (5.73) | 7.29 (5.10) | 7.29 (4.91) | 7.94 (5.73) | 9.22 (6.16) |
| 95% CI | 17.8–20.3 | 4.7–7.2 | 4.9–7.7 | 5.4–8.6 | 5.8–8.9 | 5.8–8.8 | 5.7–8.9 | 5.9–10.0 | 6.7–11.7 |
| % reduction, mean (SD) | – | 69.29 (22.47) | 68.22 (22.32) | 64.46 (24.47) | 62.19 (23.58) | 62.39 (22.56) | 61.78 (21.85) | 57.59 (24.91) | 51.93 (26.07) |
| 95% CI |  | 63.6–75.0 | 62.3–74.1 | 58.0–70.9 | 55.8–68.5 | 55.8–69.0 | 54.5–69.0 | 48.8–66.4 | 41.3–62.6 |
| CRST Part A postural (tremor severity) scores and percentage change from baseline | | | | | | | | | |
| n | 61 | 60 | 56 | 56 | 53 | 45 | 36 | 32 | 23 |
| Scores, mean (SD) | 2.77 (0.94) | 0.58 (0.83) | 0.66 (0.84) | 0.80 (0.98) | 0.68 (0.80) | 0.49 (0.69) | 0.58 (0.60) | 0.66 (0.79) | 0.91 (1.00) |
| 95% CI | 2.54–3.01 | 0.37–0.79 | 0.44–0.88 | 0.55–1.06 | 0.46–0.90 | 0.29–0.69 | 0.39–0.78 | 0.38–0.93 | 0.51–1.32 |
| % reduction, mean (SD) | – | 76.81 (31.86) | 73.66 (37.06) | 69.64 (38.74) | 75.63 (27.09) | 80.00 (28.50) | 73.15 (30.35) | 70.57 (34.33) | 67.39 (34.35) |
| 95% CI | – | 68.74–84.87 | 63.96–83.37 | 59.50–79.79 | 68.33–82.92 | 71.67–88.33 | 63.23–83.06 | 58.68–82.47 | 53.35–81.43 |
| CRST Part C (functional disability) scores and percentage change from baseline | | | | | | | | | |
| n | 61 | 60 | 56 | 56 | 53 | 45 | 35 | 32 | 23 |
| Scores, mean (SD) | 16.69 (4.87) | 4.02 (4.66) | 3.46 (3.81) | 3.75 (5.13) | 5.83 (6.07) | 6.07 (5.73) | 8.29 (7.05) | 8.41 (6.61) | 10.39 (7.66) |
| 95% CI | 15.47–17.91 | 2.84–5.19 | 2.47–4.46 | 2.41–5.09 | 4.20–7.46 | 4.39–7.74 | 5.95–10.62 | 6.12–10.70 | 7.26–13.52 |
| % reduction, mean (SD) | – | 75.93 (27.17) | 79.85 (19.87) | 80.16 (24.97) | 65.39 (34.36) | 66.19 (29.54) | 50.40 (40.25) | 45.72 (42.26) | 35.35 (45.85) |
| 95% CI | – | 69.06–82.81 | 74.65–85.06 | 73.62–86.70 | 56.14–74.64 | 57.56–74.82 | 37.07–63.73 | 31.08–60.36 | 16.19–54.51 |

**Supplementary Table 2 Total Quality of Life in Essential Tremor Questionnaire (QUEST) scores and percentage change from baseline during 5-year follow-up**

|  |  | **Follow-up** | | | | | | | |
| --- | --- | --- | --- | --- | --- | --- | --- | --- | --- |
|  | **Baseline** | **1 Month** | **3 Months** | **6 Months** | **1 Year** | **2 Years** | **3 Years** | **4 Years** | **5 Years** |
| n | 61 | 59 | 56 | 57 | 53 | 45 | 35 | 31 | 22 |
| QUEST scores | | | | | | | | | |
| Mean (SD) | 42.88 (17.40) | 19.18 (17.18) | 17.76 (18.91) | 18.58 (19.03) | 19.55 (19.79) | 21.04 (19.85) | 23.96 (18.74) | 22.51 (19.68) | 25.14 (21.92) |
| 95% CI | 38.51–47.25 | 14.80–23.57 | 12.81–22.71 | 13.64–23.52 | 14.22–24.88 | 15.24–26.84 | 17.75–30.17 | 15.59–29.44 | 15.98–34.30 |
| Percentage change in QUEST scores from baseline | | | | | | | | | |
| Mean (SD) | – | 53.39% (38.08) | 58.72% (33.93) | 58.42% (33.12) | 53.62% (43.96) | 52.56% (34.89) | 43.61% (35.54) | 45.54% (42.79) | 43.73% (39.91) |
| 95% CI | – | 43.67–63.11 | 49.83–67.61 | 49.82–67.02 | 41.78–65.45 | 42.37–62.75 | 31.83–55.38 | 30.48–60.60 | 27.06–0.41 |
