# Supplementary material for: Unilateral magnetic resonance-guided focused ultrasound for medication-refractory essential tremor: 5-year continued access study
Source: Front Neurol. 2025 Oct 22;16:1659203. doi: 10.3389/fneur.2025.1659203 (PMC12587677; doi:10.3389/fneur.2025.1659203)
Supplement: Supplementary file 2 [file Data_Sheet_1.pdf]

STROBE Statement—checklist of items that should be included in reports of observational studies

	Item No	Recommendation	Page No
**Title and abstract**	1	(*a*) Indicate the study's design with a commonly used term in the title or the abstract	1; Unilateral Magnetic Resonance-guided Focused Ultrasound for Medication-Refractory Essential Tremor: 5-Year Continued Access Study
		(*b*) Provide in the abstract an informative and balanced summary of what was done and what was found	2-3; Essential tremor (ET) is a common neurologic disorder, with 30–50% of patients experiencing medication-refractory symptoms.
**Introduction**			
Background/rationale	2	Explain the scientific background and rationale for the investigation being reported	4-5; Essential tremor (ET) is one of the most common neurologic disorders.
Objectives	3	State specific objectives, including any prespecified hypotheses	5; The objective of this study was to evaluate the long-term effectiveness and safety of MRgFUS in medication-refractory ET patients.
**Methods**			
Study design	4	Present key elements of study design early in the paper	5; This was a multi-site, open-label, prospective, single-arm, interventional study investigating unilateral MRgFUS thalamotomy for the treatment of medication-refractory ET.
Setting	5	Describe the setting, locations, and relevant dates, including periods of recruitment, exposure, follow-up, and data collection	5; Eight academic medical centers in the United States screened and enrolled subjects with medication-refractory ET between April 2015 and June 2017.
Participants	6	(*a*) *Cohort study*—Give the eligibility criteria, and the sources and methods of selection of participants. Describe methods of follow-up<br>*Case-control study*—Give the eligibility criteria, and the sources and methods of case ascertainment and control selection. Give the rationale for the choice of cases and controls<br>*Cross-sectional study*—Give the eligibility criteria, and the sources and methods of selection of participants	5-6; Eligibility criteria have been described previously.[11] The main inclusion criteria were…
		(*b*) *Cohort study*—For matched studies, give matching criteria and number of exposed and unexposed	NA

		Case-control study—For matched studies, give matching criteria and the number of controls per case	
Variables	7	Clearly define all outcomes, exposures, predictors, potential confounders, and effect modifiers. Give diagnostic criteria, if applicable	6-7; Effectiveness was assessed using the CRST at baseline compared to follow-up visits at 1, 3, 6 months and annually from 1 to 5 years.
Data sources/ measurement	8*	For each variable of interest, give sources of data and details of methods of assessment (measurement). Describe comparability of assessment methods if there is more than one group	6-7; Effectiveness was assessed using the CRST at baseline compared to follow-up visits at 1, 3, 6 months and annually from 1 to 5 years.
Bias	9	Describe any efforts to address potential sources of bias	NA
Study size	10	Explain how the study size was arrived at	NA
Quantitative variables	11	Explain how quantitative variables were handled in the analyses. If applicable, describe which groupings were chosen and why	6-7; The primary endpoint for effectiveness was evaluated using change in tremor/motor score (CRST A and B) from baseline to each follow-up.
Statistical methods	12	(*a*) Describe all statistical methods, including those used to control for confounding	7-8; For this long term follow up, no sample size or statistical analysis considerations were pre planned.
		(*b*) Describe any methods used to examine subgroups and interactions	NA
		(*c*) Explain how missing data were addressed	8; To consider the impact of subjects lost to follow up, missing data were imputed using Last Observation Carried Forward (LOCF).
		(*d*) *Cohort study*—If applicable, explain how loss to follow-up was addressed<br>*Case-control study*—If applicable, explain how matching of cases and controls was addressed<br>*Cross-sectional study*—If applicable, describe analytical methods taking account of sampling strategy	8; To consider the impact of subjects lost to follow up, missing data were imputed using Last Observation Carried Forward (LOCF).
		(*e*) Describe any sensitivity analyses	8; In addition, an extreme case analysis, consisting of best-(100% improvement) and worst-case (no improvement) scenarios, was completed to assess sensitivity.

Continued on next page

Results

Participants	13*	(a) Report numbers of individuals at each stage of study—eg numbers potentially eligible, examined for eligibility, confirmed eligible, included in the study, completing follow-up, and analysed	8-9; A total of 95 subjects provided informed consent, 34 of whom did not fulfill eligibility criteria (skull density ratio < 0.40, personal health/claustrophobia/anxiety). Figure 1
		(b) Give reasons for non-participation at each stage	9; Most subjects withdrew for reasons unrelated to the study (65.7%), including restrictions imposed by the COVID-19 pandemic, commonly during long-term follow up (years 2–5).
		(c) Consider use of a flow diagram	Figure 1
Descriptive data	14*	(a) Give characteristics of study participants (eg demographic, clinical, social) and information on exposures and potential confounders	8-9; Subjects (N = 61) had a mean (SD) age of 69.5 (14.0) years and most (67.2%) were male. Table 1
		(b) Indicate number of participants with missing data for each variable of interest	9; Due to missing data, the number of subjects with observed data for each outcome does not necessarily match the total number of subjects shown in **Figure 1.**
		(c) *Cohort study*—Summarise follow-up time (eg, average and total amount)	9; Of 61 treated subjects, 57 subjects (93%) were observed at 6 months, 53 (87%) at 12 months, and 26 subjects (42.6%) were followed for the full 5 years.
Outcome data	15*	*Cohort study*—Report numbers of outcome events or summary measures over time	9-11; Measures of effectiveness (CRST domain scores, mean and standard deviation and percentage change from baseline) are shown in **Figure 2** and **Supplementary Table 1** and at all observed time points.
		Case-control study—Report numbers in each exposure category, or summary measures of exposure	
		Cross-sectional study—Report numbers of outcome events or summary measures	
Main results	16	(*a*) Give unadjusted estimates and, if applicable, confounder-adjusted estimates and their precision (eg,	9-11, Figures 2-4 Table 2, Suppl. Tables 1 and 2; Measures of effectiveness

		95% confidence interval). Make clear which confounders were adjusted for and why they were included	(CRST domain scores, mean and standard deviation and percentage change from baseline) are shown in **Figure 2** and **Supplementary Table 1** and at all observed time points.
		(*b*) Report category boundaries when continuous variables were categorized	NA
		(*c*) If relevant, consider translating estimates of relative risk into absolute risk for a meaningful time period	NA
Other analyses	17	Report other analyses done—eg analyses of subgroups and interactions, and sensitivity analyses	10, Figure 4; The respective mean (SD) of best- and worst-case scenarios for CRST A+B, were 5.4 (5.9) and 9.3 (7.6) at 1 year follow-up; 4.2 (5.2) and 12.5 (8.0) at year 3; and 3.5 (5.8) and 15.7 (7.3) at year 5 (**Figure 4**).
**Discussion**			
Key results	18	Summarise key results with reference to study objectives	11-13; This open-label, prospective, interventional study investigated the long-term effectiveness and safety of unilateral MRgFUS for the treatment of medication-refractory ET.
Limitations	19	Discuss limitations of the study, taking into account sources of potential bias or imprecision. Discuss both direction and magnitude of any potential bias	13; In common with the pivotal clinical trial of MRgFUS with 5-year follow-up in patients with ET,[11-14] the lack of inclusion of black or Hispanic subjects represents a limitation of our study.
Interpretation	20	Give a cautious overall interpretation of results considering objectives, limitations, multiplicity of analyses, results from similar studies, and other relevant evidence	13; In conclusion, unilateral MRgFUS thalamotomy is an effective long-term treatment for patients with medication-refractory ET and is associated with improved QoL. AEs were often transient and mainly mild in severity, and no new safety signals were observed after 1-year follow-up.
Generalisability	21	Discuss the generalisability (external validity) of the study results	13; With additional data being generated from more widespread use of MRgFUS, more generalizable data may

			soon be available to address this limitation.
**Other information**			
Funding	22	Give the source of funding and the role of the funders for the present study and, if applicable, for the original study on which the present article is based	Title page (blinded); This study was funded by Insightec.

*Give information separately for cases and controls in case-control studies and, if applicable, for exposed and unexposed groups in cohort and cross-sectional studies.

Note: An Explanation and Elaboration article discusses each checklist item and gives methodological background and published examples of transparent reporting. The STROBE checklist is best used in conjunction with this article (freely available on the Web sites of PLoS Medicine at http://www.plosmedicine.org/, Annals of Internal Medicine at http://www.annals.org/, and Epidemiology at http://www.epidem.com/). Information on the STROBE Initiative is available at www.strobe-statement.org.
