# Supplementary material for: Unilateral magnetic resonance-guided focused ultrasound for medication-refractory essential tremor: 5-year continued access study
Source: Front Neurol. 2025 Oct 22;16:1659203. doi: 10.3389/fneur.2025.1659203 (PMC12587677; doi:10.3389/fneur.2025.1659203)
Supplement: Supplementary file 3 [file Data_Sheet_2.pdf]

Clinical Protocol

**Version Date**	**July 18, 2016**
**Version No.**	**1**
**Amendment No.**	**2**
**Protocol Number**	**ET002-CA**
**IDE Number**	**G120246**
**Protocol Title**	**A Continued Access Study to Evaluate the Effectiveness and Safety of ExAblate Transcranial MRgFUS Thalamotomy Treatment of Medication Refractory Essential Tremor Subjects**

Approvals:

**Site**	**Sponsor**
7/19/17<br>Principal Investigator Signature — Date	8/23/16<br>Nadir Alikacem, PhD, — Date<br>VP Global Regulatory Affairs and CRO<br>InSightec

Scanned by CamScanner

A Continued Access Study to Evaluate the Effectiveness and Safety of ExAblate Transcranial MRgFUS Thalamotomy Treatment of Medication Refractory Essential Tremor Subjects

The objective of this prospective, multisite, single-arm study is to capture the efficacy of treatment using the ExAblate Transcranial System and to further demonstrate safety in medication-refractory tremor inpatients with essential tremor (ET).

The Indications for Use claim for this system is as follows: Treatment of medication-refractory tremor in patients with essential tremor.

Protocol Number: ET-002-CA

InSightec

4851 LBJ Freeway

Suite 400

Dallas, Texas 75244

Table Of Content

1 BACKGROUND and SIGNIFICANCE 6

1.1 ExAblate MR Guided Focused Ultrasound Treatments 6

1.2 Movement Disorders 6

1.2.1 Movement Disorder in Essential Disorders Patients 6

1.3 ExAblate MR Guided Focused Ultrasound Transcranial System 8

1.3.1 ExAblate Transcranial MRgFUS System. 10

1.3.2 Rationale for ExAblate Transcranial MRgFUS Thalamotomy for the Treatment of the Essential Tremor 10

1.3.2.1 Risks associated with the current standard of practice in stereotactic surgery. 10

1.3.2.2 ExAblate Transcranial MR-guided Focused Ultrasound (TcMRgFUS) 13

1.3.2.3 Potential advantages of ExAblate TcMRgFUS Vim thalamotomy as a treatment for Essential tremor. 13

1.3.2.4 Potential risks of ExAblate TcMRgFUS Vim thalamotomy. 14

1.4 Clinical Experience with the ExAblate MR Guided Focused Ultrasound Transcranial System 15

1.4.1 Clinical ExAblate Body System 15

1.4.1.1 ExAblate Body System for the treatment of Uterine Fibroids 15

1.4.1.2 ExAblate New Software Validation (IDE #G050221) 15

1.4.1.3 Enhanced Sonication Protocol (IDE #G060017) 15

1.4.1.4 Enhanced Sonication Post Marketing Study-P040003/S007 15

1.4.1.5 Validation of ExAblate UF V2 – IDE G100127. 16

1.4.1.6 ExAblate Body System for the treatment of Breast Cancer 16

1.4.1.7 ExAblate Ablation of Breast Fibroadenoma 16

1.4.1.8 ExAblate Body System for the treatment of Prostate Studies – Investigational Feasibility Studies 16

1.4.1.9 ExAblate Body System for the Palliative treatment of Metastatic Bone Tumors 17

1.4.2 ExAblate Transcranial System 18

1.4.2.1 ExAblate Transcranial Treatment of Brain 19

2 OBJECTIVES 25

2.1 Primary Endpoints 26

2.1.1 Safety 26

2.1.2 Effectiveness 26

2.1.2.1 Efficacy Assessments 26

2.2 Secondary Endpoints 27

2.3 Study Hypothesis 27

2.4 Case Report Form Data 27

3 DESCRIPTION OF PATIENT POPULATION 27

3.1 Patient Selection 27

3.1.1 Inclusion Criteria 27

3.1.2 Exclusion Criteria 28

4 INVESTIGATION PLAN 31

Study Design Points 31

4.1.1 ExAblate Study Design 31

4.1.2 Movement Disorders Assessments 31
4.1.4 Data Safety Monitoring Board 31

4.1 Screening Procedures 31
4.1.1 Treatment Procedures 33
4.1.1.1 ExAblate Arm Treatment Procedures 36
4.1.1.2 Patient Management during Treatment 37
4.1.2 Post-Treatment Follow-up – 37
4.1.2.1 Day 1 Follow-up Procedures: 37
4.1.2.2 Week 1 38
4.1.2.3 Month 1 and 3 38
4.1.2.4 Month 6 and 12 39

4.2 Study Requirements and Visit Schedule 39

5 DATA ANALYSIS PLAN 41

5.1 Safety 41

5.2 Efficacy 41

5.3 Subject Health Status 41

5.4 Statistical Considerations and Sample Size 41

5.5 Statistical Analysis Plan. 41

5.6 Subject Confidentiality 41

6 RISK ANALYSIS 42

6.1 General Procedure Related Risks 42
6.1.1 Risk of Magnetic Resonance Scanners: 42
6.1.2 Risk of Intravenous (IV) Catheter: 42
6.1.3 Risk of Urinary Catheter: 42
6.1.4 Risk of Stereotaxic Head Frame: 43
6.1.5 Risks Incidental to the ExAblate Treatment 43
6.1.6 Risks Associated with the ExAblate Treatment 43

6.2 Anticipated Treatment Side Effects from ExAblate 50

6.3 Adverse Reactions and Precautions 51

6.4 Criteria for Removal from the Study 52

6.5 Rules for Stopping the Study 52

6.6 Adverse Event Reporting 53

6.7 Data Safety Monitoring Board 54

7 POTENTIAL BENEFITS 54

8 MONITORING PLAN 55

8.1 Electronic Data Capture (EDC) 55

9 INVESTIGATOR RESPONSIBILITIES 56

10 APPENDICES 56

11 REFERENCES 57

1 BACKGROUND and SIGNIFICANCE

1.1 ExAblate MR Guided Focused Ultrasound Treatments

ExAblate MR Guided Focused Ultrasound is an attractive modality for non-invasive thermal ablation of soft tissue tumors [1-6]. Treatment begins by acquiring a series of MR images of the target tissue. The physician then reviews the images on the ExAblate system workstation, identifies a target volume on the MR images, delineates the treatment contours on the images, and reviews the treatment plan. Therapy planning software calculates the parameters required to effectively treat the defined region. During the treatment, an ultrasound transducer generates a point of focused ultrasound energy, called a *sonication*. The sonication raises the tissue temperature within a well-defined region, causing a thermal coagulation effect. MR images acquired during sonication provide a quantitative, real-time temperature map of the entire field-of-view around the target area to confirm the location of the sonication and the size of the coagulated region. The sonication process is repeated at multiple adjacent points to cover the entire prescribed treatment volume.

1.2 Movement Disorders

Stereotactic radiofrequency lesioning and deep brain stimulation (DBS) have been used to treat a number of neurological and psychiatric diseases. Today, DBS is more commonly used to treat movement disorders, predominantly Parkinson's disease (PD), essential tremor (ET), and dystonia. In this study, the investigation will be limited to treatment of movement disorders [7] as related to ET patients.

1.2.1 Movement Disorder in Essential Disorders Patients

Essential tremor is the most common movement disorder with an estimated prevalence between 0.3% and 5.55% [8-12]. Recent epidemiological reports[1] indicate that prevalence across 19 countries (pooled) was 0.9%, but increased to 4.6% among those 65 years and as high as 21.7% in those aged 95 and older. The condition is a genetically inherited disorder with a child of an ET person having a 50% chance of inheriting a gene causing the condition. Approximately 50-70% of people diagnosed with ET have a positive family history for the condition. Caucasians are 5 times more likely to report physician diagnoses of ET than are African Americans; Hispanics have a rate between them. Gender predisposition as reported in various articles is variable depending upon the author’s population.

ET is a slowly progressive neurological disorder characterized by a tremor of the arms or hands that occurs during voluntary movements (intention tremor), such as eating/drinking and writing. The tremor may also present in the head (neck) and jaw and may affect

[1] http://www.medmerits.com/index.php/article/epidemiology_of_movement_disorders/P4

voice. The disease may present in the teens or in the 40-50 age range. Generally, tremor begins in the arms and then spreads to these other regions in selected patients. Other types of tremor may also present, including postural tremor of the outstretched arms, and intentional tremor (below 5 Hz) and rest tremor of the arms. The amplitude of an intention tremor increases as an extremity approaches the endpoint of deliberate and visually guided movement (hence the name intention tremor). An intention tremor is usually perpendicular to the direction of movement. An intention tremor causes the person to overshoots or undershoots their target, (dysmetria).

ET is commonly viewed as a pure movement disorder with a relatively benign clinical course, but specialists familiar with movement disorders recognize its associated features and disabling aspects [13]. Gait abnormalities and ataxia may accompany severe ET [14, 15], and patients may progress to develop head tremor [11] that results in more postural instability [16]. Cognitive deficits with verbal fluency, naming, and verbal memory have also been demonstrated during neuropsychological testing in patients with ET [17]. While tremor is not medically dangerous, it is very disabling and affects mental status as well as functioning at home and in the work place [18]. In fact, Lorenz *et. al*. have demonstrated that patients with ET suffer more from the mental effects on quality of life measures than from the actual physical symptom [18]. Patients with ET may have lower perceived health status [19], and some suffer significantly from social phobias [20], and depression [21]. The degree of tremor does not always correlate with the severity of disability resulting from ET [22], and so it is not surprising that the number of ET patients who may benefit from treatment is likely underestimated [21].

Up to 30% of tremor patients do not respond to first-line medical therapy, and thus may consider surgical treatment options [23]. It has long been known that stereotactic lesioning of the thalamus controls tremor, but the original targets were controversial [24]. Improved imaging with MRI and refined electrophysiological localization over the past two decades have revealed that the Vim nucleus of the thalamus is the most effective target, integrating the inflow of cerebellothalamic projections with proprioceptive and kinesthetic sensory information. Furthermore, an abundance of tremor cells which fire synchronously with the peripheral tremor can be recorded in this region [25]. With electrophysiological confirmation and identification of these cells, very small volumes of Vim (~40 mm^3) can be targeted for effective treatment [26].

Both stereotactic RF-thalamotomy and DBS targeted to the Vim have proven effective for the treatment of ET and other tremors [27]. Numerous studies of Vim ablation and stimulation have demonstrated dramatic improvements of appendicular tremors in ET, and prospective and retrospective comparisons of the two reports similar control rates of tremor with 69-90% improvement in appendicular symptoms [28, 29]. Qualitative and quantitative measures have been used to depict the benefits of thalamic stimulation in the upper extremities [30]. Axial tremors also improve with Vim stimulation [31, 32] including those tremors that extend to the voice [33]. Most importantly, quality of life in ET improves with unilateral and bilateral therapies targeted to the Vim [34-36].

A long term study of RF-thalamotomy, however, revealed that nearly 12% of treated patients experienced tremor recurrence when followed for a mean of 8.6 years [37]. DBS

is now much more widely accepted because the therapy is reversible and adjustable. Thalamic DBS is associated with long term tremor benefits [38], but tolerance to thalamic stimulation can occur in up to 30% of cases [38-41]. Furthermore, DBS has its own inherent hardware-related complications, infection, expense, maintenance demands and other risks mentioned above.

The non-invasive high-intensity focused ultrasound has been coupled with high resolution MRI to provide precise, consistent treatments that can be monitored in real-time. The development of phased array transducers allows for tightly focused treatment volumes and for the ability to compensate for distortions by tissue heterogeneity [42-44]. The landmark advance in the ExAblate TcMRgFUS for neurosurgeons occurred as the ability to sonicate through the intact cranium was achieved with phased array transducers and acoustic modeling using CT reconstructions of the skull [39, 42-45]. By coupling Focused ultrasound technology with MRI, the ExAblate system allows detailed treatment plans to be performed and real time intra-procedure monitoring [5]. Standard MR sequences have been shown to reliably predict tissue damage during thermal lesioning with ultrasound [5, 46]. The precision of the technology has already been demonstrated in animal models [47] and is currently being investigated in humans with brain tumors [48] and neuropathic pain syndromes [49]. Unlike stereotactic radiosurgery, the treatment can be monitored continuously in real time with MRI and MR-thermography [4, 50-55]. We anticipate that the ExAblate TcMRgFUS non-invasive thermal lesioning is similarly as safe as DBS (or other current procedure and will provide several years of benefit through reduction of contralateral motor symptoms and potential medication side effects in ET, while still permitting subsequent treatment with DBS should that become clinically desirable [56].

1.3 ExAblate MR Guided Focused Ultrasound Transcranial System

Ultrasound energy was shown to propagate through intact skull. Transcranial ultrasound has been used in pediatric subjects to detect midline shift of the brain[57]. In adults, blood velocity in the basal arteries may be monitored through the intact temporal bone using the Doppler effect [58]. In fact, since the 1950's, the ability of focused ultrasound to produce focal thermal lesions deep in the brain has been shown in several studies. Animal studies and early clinical studies provided encouraging results, showing well-defined tissue coagulation at the focal zone [59-62]. Fry *et al*. showed that a low frequency (around 0.5 MHz) beam could be focused through the skull [63, 64]. Their work produced thermal lesions in animal brains through a piece of skull immersed in water (bone temperature was not monitored).

However, ultrasound is strongly attenuated by bone [65]. For this reason, a consensus was reached that therapeutic ultrasound cannot be delivered through an intact skull due to the deflecting effect of the bone; the variable thickness of the skull affects the wave propagation so much that the focal spot is lost [66].

- high temperatures that are generated in the bone, due to energy absorption, could damage the scalp, bone and adjacent brain tissue.

For these reasons, previous focused ultrasound treatments of the brain have involved removal of the skull (i.e. craniotomy) for the sonication pathway [60, 67] resulting in an invasive procedure with additional risk and costs.

The device used in this study, namely the ExAblate transcranial system (see below), addresses the above limitations by combining a large phased array, active water-cooling, acoustic aberration correction algorithm, and patient specific CT data of the skull thickness registration.

Large Phased Array Transducer. The system utilizes a large phased array transducer that utilizes numerous transducer elements (current system has 1000 elements). InSightec numerous clinical studies has indeed shown that large hemispherical phased arrays can deliver adequate energy through human skulls to coagulate brain tissue in vivo without excessive temperature elevation on the skull surface [68, 69] (see **Sections 1.4.2.1** for clinical experience with ExAblate transcranial system).

Active water-cooling. The interface between the subject head and the transducer is filled with degassed water, which provides the acoustic coupling path. The system includes a chiller (refrigerating unit with degassing capabilities) that keeps the degassed water chilled at constant temperature so that the skull-bone temperature remains within safety limits.

Acoustic aberration correction algorithm. Acoustic aberration is created mostly by the variations in the bony structure of the skull. The degree of compensation necessary for each transducer element is based on predicting the aberration along the acoustic path from that element to the target and calculating the relative phase and amplitude correction necessary for that element. The result of this compensation is that the acoustic energy contribution from each element will arrive at the focal point in phase.

CT data analysis. The phase/amplitude correction algorithm, based on ray acoustics methods, relies on an input that provides the bone density profile along a ray cast between each acoustic element and the target point. This information is extracted from a three dimensional CT image of the skull [45]. This is done for each patient eligible for ExAblate treatment.

InSightec has now conducted several clinical studies with the ExAblate Transcranial system both in the United States under Investigational Device Exemption ("IDE") FDA approvals, and outside the United Sates under all applicable regulatory approvals. To date, the clinical data shows a very favorable safety and effectiveness profiles of the ExAblate transcranial MRgFUS thalamotomy procedure as well as the initial safety and effectiveness in terms of ability to ablate a targeted brain tissue (see **Section-1.4.2.1).**

1.3.1 ExAblate Transcranial MRgFUS System.

The ExAblate transcranial system combines a focused ultrasound surgery [70] delivery system and a conventional diagnostic 3T MRI scanner. This ExAblate transcranial system provides real-time therapy planning algorithm, thermal dosimetry, and closed-loop therapy control. The latter is achieved by utilizing the unique interactive MRI scan control features of the GE MRI system.

The treatment process concept of this ExAblate transcranial system is not different from the ExAblate body system which is currently in clinical use for some soft tissue applications (see Section 1.4). The treatment begins with a series of standard diagnostic MR images to identify the location and shape of tumor/target to be treated. The ExAblate computer uses the physician's designation of the target volume to plan the best way to cover the target volume with small spots called "sonications." These treatment spots are cylinder shaped. Their size depends on sonication power and duration. During the treatment, a specific MR scan, which can be processed to identify changes in tissue temperature, provides a thermal map of the treatment volume to confirm the therapeutic effect [71]. The thermal map is used to monitor the treatment in progress, and confirm that the ablation is proceeding according to plan, thus closing the therapy loop.

The ExAblate transcranial operates a helmet-shaped transducer (currently utilizing 1000-element phased array transducer) positioned above the subject head. The ExAblate transcranial system also includes means to immobilize the subject head, cool the interface water, and software for CT analysis and phase correction computation.

1.3.2 Rationale for ExAblate Transcranial MRgFUS Thalamotomy for the Treatment of the Essential Tremor

Both stereotactic radiofrequency (RF) lesions and deep brain stimulation (DBS) targeted to the Vim have proven effective for the treatment of ET and other tremors [27]. Ablation and stimulation have demonstrated equivalent dramatic improvements of tremors, with prospective and retrospective comparisons reporting 69-90% improvement in appendicular tremors [28, 29]. Stereotactic radiosurgery using the gamma knife (GK) is a third technique directed to the Vim thalamus to control tremor. The GK thalamotomy does not require an incision or bur hole, and has been reported to have efficacy equal to other lesion methods. Unlike the first two stereotactic techniques, the effects of the GK thalamotomy operation may require 6 months or more to develop.

Niranjan *et al*. [72] compared results of 15 gamma knife thalamotomies, 13 RF thalamotomies, and 11 thalamic DBS implants. They reported all three to be safe and effective, with each approach having advantages and disadvantages.

1.3.2.1 **Risks associated with the current standard of practice in stereotactic surgery.**

Hemorrhagic surgical complications

Stereotaxy uses modern, computer-assisted, volumetric imaging techniques to identify targets deep in brain in order to advance an electrode to the target. Both of these

stereotactic procedures require a scalp incision, bur hole drilled through the skull, and then penetration of the brain with an electrode to reach the target location. In any open stereotactic procedure, there is a risk of hemorrhage associated with insertion of the electrode. The overall risk of hemorrhagic complications is about 2% per electrode insertion, with a risk of permanent neurologic deficit of about 1%. Intraventricular hemorrhages occur in 5% of cases when the electrode traverses the lateral ventricular system. Typically in a stereotactic procedure, the majority of surgical complications are associated with traversal of overlying structures such as the cortex or cerebral ventricles [73].

Placement error

Target identification in stereotaxy is derived from preoperative CT or MR scans taken with the patient in a supine position [74]. The stereotactic surgery is often performed with the patient in a semi-recumbent position to minimize the loss of cerebrospinal fluid. Problems can arise under some circumstances [75] such that the brain moves relative to the preoperative scan and the calculated coordinates. This represents a potential source of error in electrode placement. Any deviation in the mechanical geometry of the electrode or the stereotactic apparatus will also contribute error which can have a considerable impact on the safety/efficacy of the treatment.

Risk from RF ablation

The electrode used for RF ablations has an RF heated tip. The peak temperature and the time it is maintained define the ultimate size of the lesion. Temperature drops off smoothly with distance from the heated tip, and there is a fairly wide zone of thermal injury that extends for several mm around the necrotic core of the lesion. The damaged tissue will rapidly produce edema which can produce local mechanical stress. The risk of perioperative hemorrhage after RF ablation is higher than after DBS implant [76]. This may be the result of damage to blood vessels within the perimeter of the lesion, in areas hot enough to damage the vessel but not hot enough to coagulate it. Mechanical strains on the damaged vessel can develop as the necrotic tissue contracts and injured tissue swells, leading to a rupture and intracranial hemorrhage. The ability to produce very sharp temperature gradients at the margins of the planned lesion would provide a more homogenous lesion and reduce the extent of potentially dangerous adjacent edematous regions.

Risk from DBS

DBS therapy has a lower risk of acute perioperative complications than does RF ablation [76]. It is also programmable and able to adapt to some degree to the typical progression of ET. However, DBS requires the permanent implantation of at least one multi-contact electrode, a lead extension and an implanted pulse generator (IPG). The DBS system will likely be in place for the life of the patient. This means the patient will need to be seen in follow-up for device management several times per year, and undergo replacement of the IPG every 3 to 5 years. Furthermore, DBS devices produce electromagnetic interference and are sensitive to high energy electrical fields which can switch them off or even cause a "factory reset" of the device.

As an implantable device, the DBS hardware problems are not uncommon. Some reports suggest that upwards of 10% of DBS patients experience some form of hardware failure, and this number is likely to rise as the existing implants age. Hardware failures can lead to a precipitous, unexpected (and possibly dangerous) loss of efficacy and invariably require surgical intervention to replace one or more components. Subjects receiving DBS implants, either unilateral or bilateral, are still subject to the same safety events and complications that are typical of thalamotomy of the Vim of the nucleus such as paresthesia and pain, dysarthria, and balance [77].

Implanted DBS hardware is associated with higher risks of infection and skin complications than lesioning procedures. The rate of postoperative infection with DBS surgery has been estimated between 3-10%, and such infections typically lead to device explanation if the infection cannot be cleared with antibiotics. Such a scenario leaves the patient without treatment. Wound dehiscence can also occur over the implanted hardware leading to infection as well.

Even though the DBS technology continues to gain acceptability, its technology remains very expensive. A bilateral Vim implant will incur an institutional cost nearing $100,000 for hardware and hospitalization. Additionally, expensive pulse generator replacements are required every three to five years.

An intervention to inactivate the Vim thalamus without requiring the use of implanted hardware would be much more economical and would avoid the considerable risks associated with chronic implants. ExAblate TcMRgFUS lesioning can be repeated should symptoms progress or recur. There is no reason why DBS could not be implemented should ExAblate lesioning be ineffective.

Risk from gamma knife thalamotomy

The GK uses ionizing radiation to denature cellular DNA and ultimately cause cell death within the area defined by the 50% isodose margins around the target. It requires a long time (median several months) [72, 78, 79], for the lesion to develop. This means that the procedure is performed without intraoperative testing to verify the target and confirm the absence of side effects. In fact, it has been reported that the lesions eventually observed on MR after 3 months are variable in volume and distribution, although the clinical effects seem consistent [79].

Because of a lack of intraoperative feedback and a small risk of radiation-induced neoplasia, GK thalamotomy remains very uncommon, restricted to patients with advanced age or medical conditions (e.g. anticoagulant therapy) perceived to be high risk for open stereotactic surgery such as DBS or RF lesioning. Because "delayed complications have been reported, and clinical improvement may take weeks to months to occur," the American Academy of Neurology concluded in 2005 that "There is insufficient evidence to make recommendations regarding the use of gamma knife thalamotomy in the treatment of ET (Level U)" [23].

1.3.2.2 ExAblate Transcranial MR-guided Focused Ultrasound (TcMRgFUS)

High-intensity focused ultrasound (HIFU) has been used to destroy soft tissue such as neoplasms for more than half a century [80]. Until very recently, lesioning brain by sonication has been difficult because the overlying skull absorbs most of the sound energy and distorts the transmitted acoustic waves. The landmark advance in ExAblate TcMRgFUS technology for neuro-related clinical applications occurred with the ability of the ExAblate to sonicate through the intact cranium [42-45]. By coupling CT-based phase tuning with ExAblate, precise and small (2x2x3mm) lesions have been produced in thalamus while real-time thermal monitoring is available to observe the heating caused with each sonication [5]. Standard MR sequences have been shown to reliably predict the precise locus of tissue damage during thermal lesioning with the ExAblate system [5, 46]. The precision of the ExAblate technology has already been demonstrated in animal models [47] and in clinical trials for neuropathic pain syndromes [49], in treatment of essential tremor (IDE # G100169), and is currently being investigated in humans with brain tumors (IDE # G020182) as well as in humans with medication-refractory tremor dominant idiopathic Parkinson's disease (IDE # G120017).

1.3.2.3 Potential advantages of ExAblate TcMRgFUS Vim thalamotomy as a treatment for Essential tremor.

As a non-invasive, non-ionized MR guided procedure with real time imaging and thermal feedback, ExAblate TcMRgFUS VIM thalamotomy has several advantages for the treatment of Essential tremor:

- The treatment can be monitored in real-time with MRI and MR-thermography [56-62]. This allows for immediate confirmation of the targeting process.
- Thermal lesioning can be performed discreetly and accurately, and can be evaluated immediately.
- Unlike stereotactic radiosurgery, ExAblate TcMRgFUS does not use ionizing radiation and does not carry a risk of radiation-induced tumorigenesis.
- Because the ExAblate TcMRgFUS procedure is non-invasive, there is no scalp incision, no bur hole, and no electrode penetrating the brain. The risk of hemorrhagic complication should be reduced, and this noninvasive procedure should eliminate the risk of infectious complications.
- Unlike DBS treatment, there is no implanted hardware, no concern of interference with external sources of electromagnetic noise, no need for extensive follow-up for programming, and no need for periodic battery replacement. This represents a much simpler treatment plan for a patient suffering from ET. There will be hours of clinic time that is saved from DBS device management and replacement. Additionally, health care costs will be greatly reduced.

- As with other surgical treatments, it will be possible to re-treat a patient who develops a recurrence of tremor if the disease progresses.

1.3.2.4 **Potential risks of ExAblate TcMRgFUS Vim thalamotomy.**

The full risks of the ExAblate TcMRgFUS thalamotomy in the treatment of Essential tremor are listed in this study protocol under **Section - 6.1** For this study, a series of risks mitigating factors have also been identified and implemented (**see Attachment – B3 of this submission**) in similar fashion as in the feasibility study (IDE # G100169). Here are two (2) potential risks, which are unique to the ExAblate system:

A- Mis-registration of the CT skull model with the MR volume.

If the CT and MR volumes are not well aligned the tuning of the ultrasonic elements will be suboptimal and distortion will make it difficult to achieve a crisp volume of heating

Risk Mitigation- The software protocols for image fusion are well-established and will be visually confirmed by clinicians experienced in stereotactic targeting. Observation of the location, size and pattern of areas heated by the low-power sonication trials will provide direct confirmation of the accuracy of the ExAblate TcMRgFUS focus. MR thermography allows for confirmation of accuracy before the process of therapeutic sonications begins.

B- Skull heating may be sufficient to damage the bone and/or adjacent soft tissues.

Risk Mitigation- The sonication times are calculated to keep skull temperatures below dangerous levels, and the minimum ten minutes allotted for skull cooling will provide time for the skull to return to normal temperature before any additional heating can take place. The small target of Vim thalamus will not typically require serial coagulations to produce effective tremor control, so cumulative effects will be minimized. Additionally, the patient will be awake, alert, and in constant communication with the investigators; so that any clinically significant heating of the scalp or skull can be immediately reported and the procedure suspended.

The safety profile of the ET001 feasibility study that was conducted under the FDA IDE # G100169 for the ExAblate TcMRgFUS treatment of subjects with medication refractory essential tremor was very favorable (**see Attachment-B2 of this submission**) .

Summary

Based on published animal and human studies, InSightec animal study, as well as the various clinical studies performed with the ExAblate system, the ExAblate TcMRgFUS Vim thalamotomy safety and effectiveness are very favorable, and compare favorably to

other surgical treatments within the currently accepted standard of care including RF lesioning and DBS. A single ExAblate lesion targeted to the VIM nucleus should provide reduction of contralateral tremor symptoms in ET (as demonstrated during the pilot study (IDE # G100169), while still permitting subsequent treatment should that becomes clinically desirable. The ExAblate technology has several potential advantages over current therapies including the fact that noninvasive lesioning can be performed in a precise manner with continuous clinical and radiographic monitoring. If the potential of the ExAblate TcMRgFUS thalamotomy can be realized, it may supplant other thalamotomy procedures (such as GK thalamotomy and RF thalamotomy), providing a viable alternative procedure for patients unable or unwilling to undergo an invasive and uncomfortable DBS procedure while still cost effective.

1.4 Clinical Experience with the ExAblate MR Guided Focused Ultrasound Transcranial System

1.4.1 <u>Clinical ExAblate Body System</u>

1.4.1.1 ExAblate Body System for the treatment of Uterine Fibroids

The ExAblate 2000 system received FDA approval for the treatment of Uterine Fibroids in October 2004 (PMA # P040003). Furthermore, this system gained both AMAR authorization (Israel Ministry of Health) and CE (European and others) approval for the indication of treating Uterine Fibroids. Subsequent studies lead to a software upgrade and an enhanced sonication protocol. A further upgrade to the system to allow the transducer arm 3-dimensional movement is currently under IDE investigation as IDE G100127

1.4.1.2 ExAblate New Software Validation (IDE #G050221)

This was an FDA-approved study to validate the new ExAblate application software as well as the use of the ExAblate system with 3T MR scanners for the treatment of UFs. This was only a safety study. A total of 40 subjects were treated under this protocol IDE. The PMA-S was approved on February 27, 2007 under P040003/S002.

1.4.1.3 Enhanced Sonication Protocol (IDE #G060017)

This was an FDA-approved study to validate the new Enhanced Sonication feature of the ExAblate system, a detachable cradle, and several other modifications to the ExAblate 2000 system. This was a safety study only. A total of 50 subjects were treated under this protocol IDE. Following completion of this study, a full PMA supplement was submitted to FDA for review and approval [PMA# P040003]. Approval was granted on 12/22/2009 under PMA Supp P040003/S006. The system is marketed under the trade name ExAblate 2000/2100 and is indicated for use in treating symptomatic uterine fibroids.

1.4.1.4 Enhanced Sonication Post Marketing Study-P040003/S007

InSightec is currently recruiting subjects for a post-market study using the FDA approved enhanced sonication feature to demonstrate the safety of the enhanced sonication feature within current treatment guidelines of 100% individual fibroid ablation within established serosal and sacral treatment margins; this study has completed its enrollment. Data

analyses and final report are in progress, and due for submission to the FDA in March-2013.

1.4.1.5 Validation of ExAblate UF V2 – IDE G100127.

InSightec is currently recruiting centers and initiating IRB review for study conduct in order to gain approval for the ExAblate Model 2100 Type 1.1 (also refer to as ExAblate UF V2). This ExAblate system will be operated with a NEW Clinical Application SW utilizing the added 5th degree of freedom of the transducer (A/P movement) in its overall planning and treatment of the uterine fibroids. This study will enroll 106 subjects under IDE # G 100127.

1.4.1.6 ExAblate Body System for the treatment of Breast Cancer

InSightec conducted FDA approved clinical trials under IDE # G990184 and G990201 to evaluate the safety and efficacy of the ExAblate system in the treatment of breast carcinomas [81-83]. Both of these studies are now closed. Currently, InSightec has an FDA conditional approval for a new breast cancer phase-2 study (IDE # G060023).

1.4.1.7 ExAblate Ablation of Breast Fibroadenoma

InSightec conducted a feasibility FDA approved clinical trials under IDE # G930140 to evaluate the safety and efficacy of the ExAblate system in the ablation of breast fibroadenoma [2]. Under this study, a total of 11 subjects were treated. The results of this study showed that 8 of the 11 subjects who had ablations were either partially (>50%) or completely (>90%) successful. No adverse effects were reported, except for one case of transient edema in the pectoralis muscle two days after therapy.

Following this feasibility study, InSightec initiated an FDA approved pivotal protocol to study ExAblate ablation of Breast Fibroadenoma (IDE # G010225). A total of 110 subjects were approved for this trial, and only 27 subjects were treated before the study was closed for enrollment due to lack of subjects enrollment. No unanticipated adverse effects have been reported or detected by MRI. Clinically, acute pain and discomfort were tolerable, and no long-term complications occurred.

1.4.1.8 ExAblate Body System for the treatment of Prostate Studies – Investigational Feasibility Studies

Prostate cancer is the second leading cause of cancer death in men in the United States. Most prostate cancers grow slowly with a high survival rate past 10 years if it is still confined to the prostate. The current treatment methods (surgical, external beam radiation therapy and male hormone suppression therapy) have significant side effects, such as impotence, incontinence, post-radiation colitis, etc. Because prostate cancer is usually slow growing, active surveillance at 6 month intervals using a prostate cancer marker (PSA – prostate-specific antigen) has been a primary treatment option. A focal treatment that could destroy the cancerous cells without harming the neurovascular bundle could provide a more palatable treatment with fewer side effects and extend life expectancy from prostate cancer causes.

Feasibility studies have been performed outside the United States to demonstrate the ability of the ExAblate to successfully target the prostate gland and ablate it (Total Gland Ablation – TGA). Additionally studies are now underway using focal therapy to ablate only cancerous foci within the prostate and leave the remainder of the gland intact. The difficulty here is in the methods available to identify the cancerous foci. These cancerous foci are generally not visible on MRI or CT, so careful, methodical biopsy mapping with multiple cores (minimum of 12 cores, commonly 16 cores for larger glands) must be performed in order to identify the portion of the gland with the cancer. In pilot studies with 14 subjects treated to date, the outcomes generally have demonstrated a minimal degree of sexual and urinary side effects (except for transient obstructive urinary symptoms) unless the cancerous foci involve the neurovascular bundles and the conscious decision is made to include them in the treatment region-of-interest. Subjects that are eligible for participation are those with slow cancer growth being followed with active surveillance or with Gleason score of 6 (3+3) and no more than 2 cancerous foci in two or fewer adjacent sectors that would be amenable to ExAblate treatment. To date, feasibility studies are underway in Russia, India, Singapore, Italy, and will soon start in Canada.

In the United States under FDA oversight, InSightec has received the full FDA approval under IDE # G100108) to initiate a feasibility study in the treatment of prostate cancer.

1.4.1.9 ExAblate Body System for the Palliative treatment of Metastatic Bone Tumors

First, InSightec performed FDA approved study for a feasibility study of ExAblate ablation of metastatic bone tumors under IDE # G050177. A total of 10 subjects were enrolled and treated at two (2) study sites. This study is now completed, and a final report was submitted to the FDA [84]. Most recently a PMA submission (PMA # P110039) with the pivotal study data was submitted to FDA on 5, DEC-2011.

1.4.1.9.1 Bone Feasibility Study IDE# G050177

The objective of this trial was to evaluate the safety and effectiveness of using ExAblate as a treatment for pain palliation in subjects with metastatic bone tumors. This study was designed as a prospective, one arm, non-randomized study. Ten subjects were enrolled at two sites. Nine subjects completed the study; one subject could not complete treatment due to limited device accessibility to the lesion.

Only 3 mild AEs were reported in the study with no device-related deaths, life-threatening injuries or permanent injuries, nor serious adverse events. There were 2 events of mild sonication-induced pain; both resolved the day that sonications ended. In addition, there was 1 event of a mild shivering reaction to conscious sedation lasting only a few minutes during the procedure with subsequent resolution. All of these events were anticipated side effects that were identified in the study protocol as possible treatment-related complications.

As noted above, effectiveness was measured by the level of pain relief (as measured by VAS), decrease in analgesics/opiate medication usage, and improved quality of life (as

measured by SF-36). Prior to ExAblate treatment, the mean pain score was 5.6 ± 1.2 (N=10, score range: 4-7). A very rapid and sustained relief response was observed in subjects' pain relief. At 3 months, the mean score had dropped to 0.4 ± 0.6 (a 93% decrease from baseline). With respect to medication usage, all subjects maintained or decreased their medication usage.

Overall, these results demonstrate the safety and effectiveness of using ExAblate as a treatment for pain palliation in subjects with metastatic bone tumors.

1.4.1.9.2 Pivotal Bone Metastasis Study (IDE# G070022) – Brief Overview

InSightec received full approval for a phase-3 Pivotal study for the ExAblate treatment of bone metastases palliation (IDE # G070022). A total of 148 subjects were to be enrolled and treated at up to 20 sites. The pivotal clinical trial was a prospective, randomized (3:1), single-blind, sham-controlled, multicenter, two-arm study with sham-crossover option. Consistent with prior correspondence between the company and FDA, InSightec has conducted an interim analysis of study data, providing for a statistical penalty addressing the early look at the data, in order to initiate PMA approval for this indication. This interim analysis has been performed under the interim statistical analysis plan previously submitted to and approved by FDA (G070022/S54). The study objective is to evaluate the safety and effectiveness of an ExAblate thermal ablation treatment as compared to a sham treatment (where no energy is delivered) to reduce/relieve the pain of metastatic or multiple Myeloma bone tumors in subjects who are not suitable candidates for radiation therapy. The submission is under review at the Agency (PMA # P110039).

1.4.2 ExAblate Transcranial System

InSightec has two ExAblate Transcranial systems: mid (650 KHz) and low frequency (200 KHz). These 2 systems (medium and low range frequency) have two different characteristics:

1. Mid frequency: functional discrete lesioning for deep central locations; focal thermal lesions
2. Low frequency: tumor ablation and has wide treatment envelope

These differences are summarized in the following table:

**Table 1: Summary of main differences between the low and mid frequency ExAblate systems**	
**Low frequency ExAblate Transcranial System**	**Mid frequency ExAblate Transcranial System**
Enables access to most of brain volume	Deep brain targets
Spot diameter: 4-12mm	Spot diameter: 2-6mm
Low frequency (~220kHz)	Medium frequency (~650kHz)
Support both standard and burst Sonication regimes	Support standard sonications only
Standard sonication delivers the required energy in a continuous fashion to the target<br>*Burst Sonication: delivers the energy in a series of burst (high amplitude short duration of each burst of energy). The total accumulated energy is the same. The only difference is the way it is delivered to the target.*	

For the proposed study, the same mid-frequency ExAblate Transcranial system that was investigated for the treatment of Essential Tremor subjects (under IDE # G100169) will be used; it should be noted this same system is utilized for the FDA approved study for tremor in Parkinson's disease subjects. The system uses the same transducer, ALL clinical features and tools of this FDA ET IDE approved system version, subject interface and coupling, etc. There is no change to the thermal modeling, energy delivery, beam forming, nor treatment parameters and guidelines, and mitigating steps. Furthermore, the manufacturing process, device risk analysis, SW and HW verification and validation have also remained unchanged.

1.4.2.1 **ExAblate Transcranial Treatment of Brain**

1.4.2.1.1 <u>Feasibility Study for Brain Tumor IDE # G020182 – ExAblate Transcranial Low Frequency System</u>

In 2002, the FDA approved an IDE for a feasibility clinical study for the ExAblate Transcranial system in the treatment of brain tumors[48]. The purpose of this study is to evaluate the safety of MRI-guided focused ultrasound thermal ablation of brain tumors performed through intact human skull using the ExAblate system. Specifically, the objectives of this non-randomized study are:

a) To evaluate the safety of FUS delivered through intact human skull to the brain, during the treatment, and during the follow-up period of 3 months.
b) To evaluate the effect of thermal ablation in the target tumor with contrast MR imaging to identify viable tumor, and non-viable thermally ablated tissue

This study was limited to subjects with a newly diagnosed glioma, recurrent glioma, or metastatic cancer to the brain for whom surgery was felt to be not indicated by a physician not associated with the study.

Per FDA order under IDE # G020182/S02, this study was approved for a total of 10 subjects. Also, per same FDA order, a report was requested after enrollment and treatment of the first 3 subjects in order to gain the FDA approval for continuation.

- During these 3 treatments, all safety subsystems and monitoring of the device provided the intended safety monitoring capabilities. The adverse events that were captured were Non-Significant, Anticipated, Treatment Side Effects and incidental to the treatment.

The treatment of the 4th subject was done with an upgraded system (approved under IDE G#020182/S04). The treatment day safety was no different than those previously reported. The skull/dura temperature change was in the range of previous treatments. Utilizing the burst sonication regime, the designated tumor was completely ablated. This was consistent with our overall plan to achieve the efficacy needed.

Despite an apparently uneventful treatment, this tumor subject died of an intracerebral hemorrhage five days after ExAblate. The Study Safety Committee determined the cause of the hemorrhage to be unknown but possibly multi-factorial. It was related to the propensity of glioblastomas to bleed, exacerbated by radiotherapy, medications and an underlying coagulopathy. The latter was suggested by the fact that this particular subject had a hemorrhage at the biopsy site long before ExAblate, skin bruising, and a peri-orbital hematoma that worsened dramatically at the time of his demise.

The neuropathologic findings raised the possibility that pre-existing changes in the vessels, such as mineralization and wall thickening, may have rendered those vessels more susceptible to damage by ultrasound at the doses or frequencies used. The Study Safety Committee recommended protocol changes in the exclusion criteria (tumors with a known tendency to bleed, subjects with abnormal clotting studies or on drugs known to affect coagulation) and in clarification of the imaging criteria (target volume maximum size requirement < 2.5 cm diameter, or an 8 cc volume - the tumor volume may be larger, as long as true midline shift is < 5 mm and the subject is not clinically compromised; definition of midline shift > 5 mm – does not include tumor growth across midline). With these provisos, the Safety Committee recommended continuation of the study. The FDA approved the recommendation of the Safety Committee under IDE # G020182/S15. The full FDA approval to restart this study was covered under IDE#G020182/S19.

1.4.2.1.2 ExAblate Transcranial MRgFUS system and Thalamotomy Procedures

The InSightec ExAblate TcMRgFUS system has been used in the thalamotomy treatment of neurological disorder such as neuropathic pain, essential tremor (“ET”), and Parkinson’s disease (“PD”) induced tremor. These studies were conducted under FDA IDE approvals (ET under IDE # G100169; enrolling for PD under IDE G # 120017), Health Canada (ET under Application # 166556), Swissmedic (NP001 study included Neuropathic pain, ET and tremor in PD subjects – Reference: 2011-MD-0001), and

Korean FDA (ET under KFDA Application # 260) oversights. Overall, more than fifty (50) subjects received the ExAblate TcMRgFUS thalamotomy procedure. It should be noted that recently, we additional studies have been approved by both FDA and Health Canada for the subthalamotomy treatment of tremor in PD patient (IDE # G140018), GPi treatment of tremor in PD patients (IDE # G140082 and Health Canada Application # 222434), and in the Blood-Brain Barrier Disruption treatment of brain tumor under Health Canada Application # 215600.

1.4.2.1.2.1 Study for Neuropathic Pain Outside the United States

The original study was an investigator initiated and sponsored study (Study number BZ001) in the treatment of neuropathic pain that was conducted at the University Hospital Zurich (Zurich Switzerland) using the InSightec ExAblate Transcranial (650 KHz) system. The study was approved by and performed according to the guidelines of the ethics committee of the University and the State of Zurich. At the conclusion of this study, an InSightec sponsored study was conducted under Swissmedic (Reference #: 2011-MD-0001) and local IRB approvals. This study (NP001) included subjects with Neuropathic pain, Essential tremor, and those subjects with Parkinson's disease induced tremor.

To date, more than thirty-five (35) subjects underwent selective central lateral thalamotomy (CLT) using the ExAblate Transcranial treatment. For all subjects, the treatment was well tolerated and did not result in any side effects or neurological deficits. The safety profile of these procedures was no different from those known to occur in any thalamotomy procedure such as DBS or RF ablation. To be specific, all the reported adverse events observed in these studies were all non-significant and anticipated events already identified as potential events in the study protocol. These were headaches, nausea, and vomiting. All resolved on the same day as treatment. The only significant event reported to date from these two (2) studies an event of neurological deficit (Subject BZ001-12), i.e. "dysmetria (dyscoordination) of the right hand, dysarthria, motor neglect and gait disorder". This event, originally reported as part of IDE # G100169, was reported immediately following the last sonication. All symptoms improved significantly 1-hour post treatment. By 24 hours, 70-80% of the motor symptoms had abated and with time all dysmetric symptoms cleared except when the subject tried to write or speak (see Jeanmonod *et al* paper[2]).

As it was shown in the brain tumor study under IDE G020281, for these studies there was no clinically significant heating at the skull-brain interface. The mean brain surface temperature was approximately 39° C.

Other than a couple of subjects, all Neuropathic pain treated subjects (N=19) experienced some level of pain relief during the procedure, and at 48 hours after the treatment,

---

[2] Jeanmonod *et al*. "Transcranial magnetic resonance imaging-guided focused ultrasound: noninvasive central lateral thalamotomy for chronic neuropathic pain". *Neurosurg Focus*, 2012, Jan 32: E1

subjects reported pain relief ranging from 32 to 100% (mean ≈ 65%). Partial results of this study were published in the *Annals of Neurology Journal* [3]

1.4.2.1.2.2 Study for Tremor in Parkinson Disease subjects - Outside the United States

As stated above, the Swissmedic NP001 included cohort of six subjects with Parkinson's disease induced tremor. Following the ExAblate treatment, the tremor suppression ranged from 20 to 50% at the 3-Months time point in 5 out of the 6 treated subjects.

1.4.2.1.2.3 Study for Essential Tremor - Outside the United States

In addition to the Swissmedic study that included cohort of 1 subject (study is still on-going) with Essential tremor, InSightec conducted separate studies for the treatment of Essential tremor in both Canada and in Korea. A total of 8 subjects were treated to date as follows: 3 subjects in Canada, and 5 subjects in Korea. Both of these study protocol were similar to the FDA feasibility study (IDE G#100169). Overall, the ExAblate treatment safety profile is no different from the FDA feasibility study, nor from the safety profile of typical thalamotomy device procedures such as RF ablation and DBS. In short, safety events such as Dizziness and vomiting were reported, and both resolved without sequelae.

1.4.2.1.2.4 Feasibility Study for Essential Tremor IDE # G100169

InSightec received FDA approval for a feasibility trial of ExAblate Transcranial System for unilateral thalamotomy in the treatment of Essential Tremor under IDE # G100169. Total of 15 subjects were enrolled and treated at one site. This study has been completed and the full results of this study were published in the *New England Journal of Medicine.* [73] Based on the investigator and the subject's feedback, subjects have shown a great level of acceptance of the procedure. Furthermore, subjects have shown a significant improvement in their Essential Tremor disease following their unilateral treatment with the ExAblate Transcranial device. Subjects who completed the study requirements have shown stability of the tremor suppression all the way to the end of the study.

1.4.2.1.2.5 Pivotal Study for Essential Tremor – IDE # G120246

This is a global, multi-center, randomized, sham-controlled pivotal study to evaluate the safety and efficacy of ExAblate Transcranial unilateral thalamotomy for treating medication-refractory Essential Tremor. A total of 72 subjects will be recruited for this study which has just gotten underway. After the last subject completes 1 year follow-up, a Pre-Market Approval submission will follow.

---

[3] Martin, E., et al., *High-intensity focused ultrasound for noninvasive functional neurosurgery.* Ann Neurol, 2009. **66**(6): p. 858-61.

1.4.2.1.2.6 Feasibility Study for Tremor Dominant Parkinson's Disease – IDE # G120017

This is a, multi-center, randomized, sham-controlled study to evaluate the safety and efficacy of ExAblate Transcranial unilateral thalamotomy for treating medication-refractory Tremor Dominant Parkinson's Disease. A total of 30 subjects will be recruited for this study. After the last subject completes the study, a final clinical report will be written and submitted to FDA. To date, 18 patients have been treated.

1.4.2.1.2.7 Feasibility Study for Unilateral Lesioning of the Subthalamic Nucleus in treatment of Motor Symptoms of Advanced Idiopathic Parkinson's Disease - IDE # G140018

This is a study to evaluate the safety and efficacy of ExAblate Transcranial unilateral Sub-Thalamotomy for treating motor symptoms of advanced idiopathic Parkinson's disease. A total of 10 subjects will be recruited for this study. After the last subject completes the study, a final clinical report will be written and submitted to FDA.

1.4.2.1.2.8 Feasibility Study for Pallidotomy in treatment of Dyskinesia of Advanced Idiopathic Parkinson's Disease - IDE # G140082

This is a study to evaluate the safety and efficacy of ExAblate Transcranial unilateral Pallidotomy for treating motor symptoms of advanced idiopathic Parkinson's disease. A total of 20 subjects will be recruited for this study. After the last subject completes the study, a final clinical report will be written and submitted to FDA.

1.4.2.1.2.9 Feasibility Study for Pallidotomy in treatment of Dyskinesia of Advanced Idiopathic Parkinson's Disease - Health Canada Application # 222434

This is a study to evaluate the safety and efficacy of ExAblate Transcranial unilateral Pallidotomy for treating motor symptoms of advanced idiopathic Parkinson's disease. A total of 6 subjects will be recruited for this study. After the last subject completes the study, a final clinical report will be written and submitted to Health Canada.

1.4.2.1.2.10 Feasibility Study for Blood Brain Barrier Disruption in the treatment of Brain Tumors – Health Canada Application # 215600

This is a study to evaluate the safety and efficacy of ExAblate Transcranial unilateral for the BBB disruption for the treatment of brain tumors. A total of 10 subjects will be recruited for this study. After the last subject completes the study, a final clinical report will be written and submitted to Health Canada.

1.4.2.1.2.11 CE Approval of the ExAblate Transcranial MRgFUS System Tremor - Outside the United States

In December-2012, InSightec received CE Mark of Conformity approval for the ExAblate Model 4000 Type 1 for the following Indication of Use:

Intended use	ExAblate 4000 transcranial MR guided focused ultrasound (TcMRgFUS) system (type 1) intended for thermal ablation of targets in the thalamus, sub thalamus and Pallidum regions of the brain.
Indication for use	ExAblate 4000 transcranial MR guided focused ultrasound (TcMRgFUS) can be used for the treatments of neurological disorders (Essential Tremors, Tremor Dominant Idiopathic Parkinson’s Disease – Unilateral) and Neuropathic pain in the brain by heat induced focusing using ultrasound energy under full MR planning and thermal imaging control.

The ExAblate system for this submission and all its components including and not limited to patient interface, Hardware, Software is the same system as the one currently approved under IDE’s G020182/S24, G120017 (including Supplement S11), G120246 (including Supplement S04), G140018, and G140082.

2 OBJECTIVES

The objective of this prospective, multi-site, single-arm, open-label study is to capture the efficacy of treatment using the ExAblate Transcranial System and to further demonstrate safety in subjects with medication-refractory tremor essential tremor (ET).

Safety: To evaluate the incidence and severity of adverse events (AE/AEs) associated with ExAblate Transcranial MRgFUS treatment of medication-refractory ET

Effectiveness: Collect further evidence of the effectiveness of the ExAblate Transcranial MRgFUS treatment of medication-refractory tremor (i.e. ET). Efficacy will be determined utilizing the Clinical Rating Scale for Tremor (CRST) in ET from examinations at baseline and 3-Months post-ExAblate treatment.

- This study is designed as a prospective, multi-site, single-arm, open-label study.

Assessments of primary efficacy endpoints will compare the three months after ExAblate treatment to Baseline measurements for clinical symptom relief. Safety of ExAblate in the treatment of ET will be collected for one year after ExAblate treatment. Relative Safety will be evaluated using a common description of Significant Clinical Complications for patients treated in this study. This study will be performed on the 3T MR scanners.

Note:

It should be noted that the pre-market submission to the FDA will be performed following the completion of the 12 months follow up visit of the last Test Arm subject. This continued access study will allow sites to continue to perform the procedure and to collect additional safety and efficacy data that will be part of the FDA review of the PMA submission for ET002 "IDE G120246. However, per FDA order, all participating subjects will be consented for a total of 5 years.

The second objective of this study is to collect evidence of cost data associated with the management and treatment of the proposed study cohort of subjects to support submission to the Centers for Medicare and Medicaid Services ("CMS") for code and reimbursement assignment levels for the ExAblate Neuro device. For this purpose, all participating centers will be expected to provide to InSightec representatives in full confidentiality the associated forms (Forms "UB-04 (In Patient subjects) or CMS-1500

(Out Patient subjects) of enrolled and treated subjects. All forms must be de-identified from all patient and institution identifiers and qualifiers prior to sharing them with InSightec representatives.

2.1 Primary Endpoints

2.1.1 Safety

Safety of ExAblate will be determined by an evaluation of the incidence and severity of device / treatment related complications from the first treatment day visit through ALL study follow ups. Adverse events (type, frequency, severity) are expected to be similar to those of previous studies using ExAblate TcMRgFUS for Thalamotomy

All AEs will be reported and categorized by investigators as definitely, probably, possibly, or unrelated to the device or procedure and categorized by treatment group / treatment arm.

2.1.2 Effectiveness

Primary effectiveness will be evaluated using a validated, tremor rating scale: the Clinical Rating Scale for Tremors (CRST) for ET subjects, based upon subjects in whom unilateral ExAblate lesioning is attempted (i.e., Intent-to-Treat analysis).

Clinical assessments will be made at the following time points: screening, baseline with subjects on confirmed stable medication (at 1-months post screening), and post-treatment at 1 week, 1 month, 3 months, 6 months, and 12 months. Baseline and follow-up efficacy assessments will be performed by a site neurologist who is a movement disorders specialist.

2.1.2.1 **Efficacy Assessments**

Tremor will be assessed for each treated ET patient using the CRST [85] at baseline following the medications observation period (i.e.: subjects on confirmed stable medication) but before treatment and at post-treatment intervals: 1 month, 3 months, 6 months and 12 months. The validated rating assessment of tremor will be administered by a movement disorder specialist (e.g.: neurosurgeon, or neurologist or physical therapy specialist in movement disorder). The primary measure utilized in this protocol will be a reduction in the treated (contralateral) upper extremity CRST applicable subscores. Additionally, a total/overall tremor score (out of 160 points) will be obtained for each ET patient at each time interval by summing the appendicular and axial scores.

Quality of life will be evaluated using the QUEST assessment. The QUEST is an ET specific assessment of quality of life changes associated with ET. Tröster *et al*., 2005, developed QUEST as a clinical tool for correlating changes in 30 aspects of tremor severity, social and personal disability, and perception of health. An independent

validation study of the QUEST performed by Martinez-Martin *et al*. concluded that most of the psychometric parameters were found to be satisfactory in their ability to assess the impact of ET on the patients' quality of life.

2.2 Secondary Endpoints

The secondary endpoints of the study are as follows:

1. Quality life claims: Questionnaire for Essential Tremor (QUEST) outcome (upper extremity questions) at Months 3 change from Baseline as compared between treatment groups
2. Durability (as measured by CRST upper arm extremity questions) of the procedure as reflected by the efficacy data through change from baseline measures through Month 12 follow up
3. Subject daily functionalities: as measured by CRST Part-C (subscales) Month 12 as compared to Baseline, and between treatment groups through Month 3.

2.3 Study Hypothesis

The purpose of this study is to provide additional safety and efficacy data to support the indication of MRI-guided focused ultrasound thermal ablation of a designated area in the brain of patients suffering from medication-refractory ET using the ExAblate transcranial system.

- For ET Patients: a unilateral thermal lesion will be created in the *ventralis intermedius* nucleus of the thalamus [54]

2.4 Case Report Form Data

The study data will be collected electronically. This electronic data capture (EDC) system complies with the current guidance of 21 CFR Part 11, Electronic Records and Signatures.

3 DESCRIPTION OF PATIENT POPULATION

3.1 Patient Selection

Patients with confirmed medication-refractory ET as confirmed by screening will be eligible for this study; full copy of subject consent is provided as **Appendix-A** of this protocol.

3.1.1 Inclusion Criteria

1. Men and women age 22 years or older
2. Subjects who are able and willing to give consent and able to attend all study visits,

3. A diagnosis of ET as confirmed from clinical history and examination by a neurologist or neurosurgeon specialized in movement disorder
4. Tremor refractory to adequate trials of at least two medications, one of which should be a first line therapy of either propranolol or primidone. An adequate medication trial is defined as a therapeutic dose of each medication or the development of side effects as the medication dose is titrated.
5. Subjects must be on stable medication for tremor prior to treatment. Stable is defined as on the same drug/same dose for a minimum of 30 days and meets eligibility criteria.
6. Vim nucleus of thalamus can be target by the ExAblate device. The thalamic region must be apparent on MRI such that targeting can be performed by measurement from a line connecting the anterior and posterior commissures of the brain.
7. Able to communicate sensations during the ExAblate TcMRgFUS treatment
8. Postural or intention tremor severity score of greater than or equal to 2 in the dominant hand/arm as measured by the CRST rating scale while stable on medication.
9. May have bilateral appendicular tremor
10. Significant disability due to essential tremor despite medical treatment (CRST score of 2 or above in any one of the items 16-23 from the Disability subsection of the CRST: [speaking, feeding other than liquids, bringing liquids to mouth, hygiene, dressing, writing, working, and social activities])
11. Inclusion and exclusion criteria have been agreed upon by two members of the medical team.
12. Subjects on stable antidepressant medications for at least 3 months may be enrolled into this study (i.e., no change in medication drug or dosage for 3 months).

3.1.2 <u>Exclusion Criteria</u>

1. Subjects with unstable cardiac status including:
    - Unstable angina pectoris on medication
    - Subjects with documented myocardial infarction within six months of protocol entry
    - Significant congestive heart failure defined with ejection fraction < 40
    - Subjects with unstable ventricular arrhythmias
    - Subjects with atrial arrhythmias that are not rate-controlled

2. Subjects exhibiting any behavior(s) consistent with ethanol or substance abuse as defined by the criteria outlined in the DSM-IV as manifested by one (or more) of the following occurring within a 12 month period:

    - Recurrent substance use resulting in a failure to fulfill major role obligations at work, school, or home (such as repeated absences or poor work performance related to substance use; substance-related absences, suspensions, or expulsions from school; or neglect of children or household).
    - Recurrent substance use in situations in which it is physically hazardous (such as driving an automobile or operating a machine when impaired by substance use)
    - Recurrent substance-related legal problems (such as arrests for substance related disorderly conduct)
    - Continued substance use despite having persistent or recurrent social or interpersonal problems caused or exacerbated by the effects of the substance (for example, arguments with spouse about consequences of intoxication and physical fights).

3. Severe hypertension (diastolic BP > 100 on medication)
4. Subjects with standard contraindications for MR imaging such as non-MRI compatible implanted metallic devices including cardiac pacemakers, size limitations, etc.
5. Known intolerance or allergies to the MRI contrast agent (e.g. Gadolinium or Magnevist) including advanced kidney disease
6. Patient with severely impaired renal function with estimated glomerular filtration rate <30 $mL/min/1.73m^2$ (or per local standards should that be more restrictive) and/or who is on dialysis;
7. History of abnormal bleeding and/or coagulopathy
8. Receiving anticoagulant (e.g. warfarin) or antiplatelet (e.g. aspirin) therapy within one week of focused ultrasound procedure or drugs known to increase risk or hemorrhage (e.g. Avastin) within one month of focused ultrasound procedure
9. Active or suspected acute or chronic uncontrolled infection
10. History of immunocompromise including those who are HIV positive.
11. History of intracranial hemorrhage
12. Cerebrovascular disease (multiple CVA or CVA within 6 months)
13. Subjects with uncontrolled symptoms and signs of increased intracranial pressure (e.g., headache, nausea, vomiting, lethargy, papilledema).
14. Individuals who are not able or willing to tolerate the required prolonged stationary supine position during treatment (can be up to 4 hrs of total table time.)

15. Are participating or have participated in another clinical trial in the last 30 days
16. Significant claustrophobia that cannot be managed with mild medication.
17. Subjects unable to communicate with the investigator and staff.
18. Presence of any other neurodegenerative disease such as Parkinson-plus syndromes suspected on neurological examination. These include: multisystem atrophy, progressive supranuclear palsy, dementia with Lewy bodies, and Alzheimer's disease.
19. Anyone suspected to have the diagnosis of idiopathic Parkinson's disease. Anyone with the presence of parkinsonian features including bradykinesia, rigidity, or postural instability will be excluded. Subjects who exhibit only mild resting tremor but no other symptoms or signs of PD may be included.
20. Presence of significant cognitive impairment as determined with a score ≤ 24 on the Mini Mental Status Examination (MMSE)
21. Subjects with life-threatening systemic disease that include and not limited to the following will be excluded from the study participation: HIV, Liver Failure, blood dyscrasias, etc.
22. Subjects with a history of seizures within the past year
23. Subjects with presence or history of psychosis will be excluded. Subjects with significant or active mood disorders including depression will be excluded. For the purpose of this study, we consider a significant mood disorder to include any subject who:
    - Scores ≥ 20 on the PHQ-9 questionnaire
    - Is currently under the care of a psychiatrist
    - Is currently participating in cognitive-behavioral therapy
    - Has been hospitalized for the treatment of a psychiatric illness within 12 months
    - Has ever received transcranial magnetic stimulation
    - Has ever received electroconvulsive therapy
24. Subjects with risk factors for intraoperative or postoperative bleeding: platelet count less than 100,000 per cubic millimeter, INR coagulation studies exceeding local institution laboratory standards, or a documented coagulopathy
25. Subjects with brain tumors
26. Any illness that in the investigator's opinion preclude participation in this study.
27. Pregnancy or lactation.
28. Legal incapacity or limited legal capacity.

29. Subjects who have had deep brain stimulation or a prior stereotactic ablation of the basal ganglia
30. Subjects who have been administered botulinum toxins into the arm, neck, or face for 5 months prior to Baseline.
31. Subjects who have an Overall Skull Density Ratio of 0.45 (±0.05) or less as calculated from the screening CT.

4 INVESTIGATION PLAN

Study Design Points

4.1.1 ExAblate Study Design

The eligible Subjects will proceed to the ExAblate treatment and be followed up for 3-months post-ExAblate treatment to assess the primary study endpoints at study follow-up: 1day, 1week, 1 and 3 month visits.

All subjects will be followed for 12-months post-treatment to assess the treatment long term safety and durability with study visits at 6 and 12 months.

4.1.2 Movement Disorders Assessments

For this study, the efficacy endpoint assessment from Baseline (i.e. immediately post medication observation/stability) through the Month 12 follow up visit will be performed by a site assessor at each participating site.

4.1.4 Data Safety Monitoring Board

A Data Safety Monitoring Board (DSMB) will be used to review all AEs on the study. Their role is to evaluate all AEs that occur throughout the study and provide their assessment of the study safety profile as well as their recommendations for the study continuation. The DSMB recommendations will be communicated to the FDA as part of the annual reporting for this proposed IDE study.

All adverse events will be assessed for their relationship to the study device or procedure. Standard Code of Federal Regulation (CFR) definitions for SAEs and UADEs will be used in assessment of adverse events (**see Section-6.5**).

4.1 Screening Procedures

The following steps are common to all subjects

1. Subjects believed to have medication-refractory ET movement disorder will be offered an Informed Consent to sign prior to further evaluation. Those who

accept and sign the consent will be assigned a subject study identification number (ID#).

2. The diagnosis of ET will be confirmed by a neurosurgeon or neurologist specializing in movement disorders, where a comprehensive neurologic examination will be performed, including the screening CRST
3. Medication refractoriness will be defined when tremor persists despite adequate doses of at least trials of appropriate medical therapy. At least one of the medications trialed should be a first line agent, namely propranolol or primidone. The other medication trialed should be a class B agent as defined by the AAN (2005 and 2011 references). Adequate trial (*daily*) dosages are noted below or occur if dose-limiting side effects occur:

First line (class A) tremor therapy:

- Primidone 250 mg
- Propranolol 240 mg
- Propranolol LA 240 mg

Second line (class B) tremor therapy:

- Gabapentin
- Topiramate 400 mg
- Other Beta blockers: Atenolol or Sotalol

  Benzodiazepimes: Alprazolam or Clonazepam

CT Imaging:

For the purpose of this study, the CT exam should be an Axial scan with bone filter, an image resolution of 512x512, and image thickness of 1mm with zero (0) spacing. This CT imaging will be utilized to compute the Skull Density ratio, and as part of the treatment planning should the subject qualifies for the study.

During this one month "medication observation" phase, the current regimen of medications will be maintained to ensure tremor stability. Screening laboratory and imaging studies will be obtained to complete the enrollment process.

4. A complete medical history will be obtained to determine subject's general health status; including blood analyses (for PT, PTT, CBC, platelets, and creatinine), and pregnancy urine test for women of childbearing age. Positive test will exclude subjects from study. In the event the pregnancy test is negative, she will be asked to use an acceptable form of contraception throughout entire course of study.
5. PHQ-9 depression will be obtained. All subjects who score ≥ 20 on the test will be excluded from the study.
6. Medication stability and baseline measurements:

In this study, all subjects will be tested again at the one month time point (±7days) post screening date. At this visit, the following will be performed:

- Medication stability: subjects will be asked to report any changes in medication taken for tremor consumed over the preceding month.
- All those subjects on antidepressants must be on the same antidepressant medication at the same dose for the prior 3 months to be eligible.
- Once the medication stability is confirmed, then all other baseline assessments will be performed;
    - CRST must be performed by a movement disorders specialist.
    - QUEST and imaging will also be performed after medication stability has been demonstrated.

7. If at this point it is determined that the subjects ***do not*** meet all Inclusion and Exclusion criteria and cannot be treated, the subjects will be exited from the study. These subjects will be considered screening failures, and will not be included in any of the safety or efficacy endpoint analyses.

8. If the subject meets all the pre-imaging criteria, then the subjects will be scheduled for the MR .

   ➢ MR Imaging: For the purpose of this study, **the following** MR Exams will be performed.

      1. The pre-contrast MR Imaging should include the following:

         a) T2 Weighted imaging exam along these 2 axes: Axial and Sagittal

   ➢ CT Imaging: For the purpose of this study, a CT exam will be performed and used to both determination of Skull Density Ratio and during treatment for determining skull contours.

8. A second physician specializing in movement disorders will review all the inclusion/exclusion criteria and verify that the subject meets the study screening criteria prior to randomization.

9. Subjects who have been verified as meeting all study criteria may be scheduled for the ExAblate treatment.

4.1.1 Treatment Procedures

WARNING:

In this study, subject participation is necessary during the course of the ablation procedures. USE OF SEDATIVE MEDICATION MAY IMPAIR COGNITIVE FUNCTIONS OF THE SUBJECT.

However, to alleviate subject anxiety that can develop during a lengthy procedure inside the MR scanner, low dose sedatives like benzodiazepines or dexmedetomidine or equivalent may be administered by slow titration so as

to not impair the level of consciousness. Subjects MUST always remain responsive to physician instructions.

Any subject who is not appropriately responsive will not be allowed to continue on with any further treatment procedures until it is deemed clinically safe by the treatment team. Sedation reversal with flumazenil or equivalent may be utilized if excessive sedation occurs. If a subject cannot participate in the treatment after receiving sedation, the procedure will be aborted, and the subject will be considered as screen failure

The following procedure steps will be performed as follows:

Subjects will present to the ExAblate TcMRgFUS treatment center on the morning of surgery "*off*" medication.

1. An IV line of saline will be positioned for the delivery of fluids and any medications required during the procedure. Some of the subjects may require a urinary catheter to keep the bladder empty during treatment. Noninvasive monitoring of heart rate, blood pressure, systemic oxygen saturation, electrocardiogram, and end-tidal CO_2 will be maintained throughout the procedure using standard MR-compatible monitoring devices.
    - The anesthesiologist, or nurse anesthetist, or treating physician will be present to monitor subject's overall well being. Their major role is to monitor and control of blood pressure to prevent hypertension during the procedure. Subjects must be able to communicate during the entire procedure, and must be able to operate the stop sonication button.
    - To address emergency situations should they occur, a standard ventilator should be available in close proximity to the MR scanning room; per local site clinical standards.
2. The subject's head will be carefully shaved and examined for pre-existing scalp scars or any other scalp lesions.
3. Graduated compression stockings will be worn to prevent deep venous thrombosis in the lower limbs.
4. The subject's head will be placed in the stereotactic head frame (similar to those used in stereotactic surgery and radiotherapy) for use during the actual ExAblate TcMRgFUS treatment.
5. Subject will be positioned supine and headfirst on the MR/ExAblate therapy table.
6. The half-spherical helmet containing the elements of ExAblate transducer will be positioned around the subject's head in the treatment position. This should be done according to measurements taken from the pre-operative/imaging session(s).
7. An elastic membrane will be attached to the Subject's head and to the transducer to create the acoustic coupling system between the ultrasound transducer and the scalp.

8. The immobilization system will be secured over the subject head to maintain a constant relationship between the target and the transducer.
9. A baseline assessment of symptoms of tremor will be obtained before the ablation procedure begins
10. The interface between the scalp and the transducer (within the elastic membrane) will then be filled with degassed water. This volume will be completely filled with care to avoid air bubbles between the face of the transducer and the scalp. Through active circulation and cooling system, the water will be maintained chilled throughout the procedure to avoid undesired heating of the scalp and skull.

> ➢ **The water circulation and temperature status are continuously displayed on the clinical workstation. Ensure that water is continuously at its base temperature of 15 ± 3C prior to starting the procedure or any subsequent sonication(s).**

11. A 3D localizer MR scan will be obtained to allow further refinement of the position of the ExAblate transducer focal point with respect to the targeted zone.
12. A series of MR images will be acquired to identify the target area, and plan the actual treatment
    - T2 Weighted imaging exam along at least 2 axes: Axial and Sagittal
13. The pre-therapy MRI and CT image datasets will be registered to the T2 weighted MR images that were just acquired. This image fusion of pre-operative MR assists in the accurate delineation of the target area and determination of a safe sonication pathway
    - The fusion of the CT data is required for the computation of phase correction values to correct for skull aberration, and identification of intracranial calcifications
    - Scars of the scalp will be designated to ensure the ultrasound beam avoids these specific areas
14. The treatment volume and plan will be defined by the neurosurgeon. The ExAblate TcMRgFUS system will automatically compute the number of sonications, and the (per sonication spot) phase and amplitude corrections necessary for the system to produce a focal spot at each of the desired locations.

<u>At this stage, all those subjects who do not meet the MR requirement will be considered screen failures</u>.

> ➢ **Prior to starting the actual Sonication Procedure(s), ensure that (1) the water temperature is at its base of 15 ± 3C, (2) that**

the water circulation has been on for at 20 – to - 30 minutes to ensure patient scalp is being cooled. It should be noted that typically this period of time would correspond to the imaging time of steps-11-13 above.

4.1.1.1 ExAblate Arm Treatment Procedures

The following will be performed for all ExAblate subjects:

1. A central point in the targeted area will be targeted with a low dose, sub-lethal energy level sonication to confirm the targeting accuracy on the MR images. Focal point position and/or transducer location will be adjusted as necessary.

   WARNING:

   The subject MUST be examined by the clinical team after each sonication for neurologic signs and symptoms and evidence of tremor suppression.

2. To enhance the procedure safety and mitigate some of the inherent risks of thermal lesioning of brain tissue:

   a) The ExAblate Transcranial Thalamotomy will be performed as a series of sonications with increments in power within the designated target volume in the awake subject.

   b) The subject will be examined by the Subject Assessor during & after each sonication for neurologic signs and symptoms, and evidence of tremor suppression. The subject assessor and subject are in direct communication with the neurosurgeon and treatment team at all times from within the MR suite.

   c) The series of sonications will start with low energy prior to permanent thermal ablation. This is to ensure the planned sonication to be centered on the Vim nucleus of the Thalamus. Low energy sonication will non-destructively warm the target. The warming will be captured by the MR thermometry and the MR thermal images will be displayed in real time to the treating physician. The neurosurgeon will then verify that the warming is centered on the prescribed anatomic target. This will allow the centering of the eventual permanent thermal lesion in the planned location.

   d) The titration of escalating focal sonications will continue up to full ablation of the targeted planned area for ablation. This would be performed by utilizing the full feedback that is provided by the real time MR Thermometry.

For this study, ONLY a unilateral lesioning/ablation will be performed using the ExAblate TcMRgFUS system.

4.1.1.2 Patient Management during Treatment

The following treatment procedure instructions are applicable to all subjects

- The ExAblate TcMRgFUS system is equipped with Stop Sonication Buttons: one for the subject to utilize, one for the nurse/anesthesiologist (subject monitor), and one for the treating physician to use. Hence, in the event of discomfort or pain, the subject will have the ability to abort the sonication at any time by activating the Stop Sonication Button. Once this button is activated, the system will instantly stop the energy delivery. The same thing will happen in the event that the treating physician or the subject monitor activates their button. After addressing the subject concerns or discomfort, the procedure may continue without further delay. All adverse events that may be caused by these potential activation(s) of the Stop Sonication Button will be captured on the CRFs.
- After the treatment or within 24 hours of treatment, a series of MR images will be acquired to assess the treatment effects:
    a. T2 Weighted sequences to assess for peri-lesional edema

Note: In the event new neurological deficits or seizures are observed, other imaging modalities (including CT) should be performed immediately per standard of care of local site for similar events. These are in addition to neurological and physical examinations.

- Once the treatment is complete or otherwise terminated:
    a. Remove the subject from the ExAblate table and remove all monitoring equipment, elastic membrane, and stereotactic frame.
    b. Document neurological and physical examinations.
- The subject will be transferred to the hospital for recovery and neurological observation until the next morning. Discharge from the hospital is at the discretion of the physician based upon the subject's overall well-being. Holding the subject for an additional night does not constitute a serious adverse event.

ALL imaging exams of this study must be forwarded to the Sponsor in a timely fashion immediately following their acquisition and properly anonymized from all subject identifiers.

4.1.2 Post-Treatment Follow-up –

4.1.2.1 Day 1 Follow-up Procedures:

The neurosurgeon will perform the following exams and make a decision as to whether or not to discharge the subject. Subjects who are found to be neurologically unstable will remain in the hospital until the neurosurgeon determines it is medically indicated to discharge.

a) Review of medications
b) physical exam
c) neurological status

Any adverse events will be recorded and followed. The physician should treat the subject according to their best clinical judgment if adverse events are noted.

4.1.2.2 **Week 1**

The following evaluations should be performed at Week 1:

- Review of medications
- Physical exam
- Neurological status
- Adverse events

4.1.2.3 **Month 1 and 3**

The following evaluations should be performed at 1 & 3 months:

- Review of medications
- Physical exam
- Neurological Status
- CRST
- QUEST and PHQ-9-depression questionnaires should be completed by the subject
  - NOTE:

    At any follow up visit, any subject who scores ≥ 20 on the PHQ-9 questionnaire, the subject should be referred out for psychiatric evaluation. The Columbia Suicide Severity Rating Scale, or like, may be used. Should there be a psychiatric treatment, then this psychiatric treatment should be recorded as an adverse event. A Copy of the psychiatric evaluation should become part of the CRF information as part of the adverse event outcomes and description. The investigator will evaluate whether the subject may continue participation in the study, or should be exited from the study for treatment
- Adverse events

4.1.2.3.1 Reasons for Study Exit

In this study, subjects who opt for alternative treatments for Essential Tremor (not including medication change) at any point in the follow-up period will be exited from the study after completing required study examinations. The last set of evaluations prior to alternative therapy is considered the last study visit. The reason(s) for study exit will be

noted on the Case Report Forms. No analyses of post alternative treatment changes are planned.

4.1.2.4 Month 6 and 12

To assess treatment long term safety and efficacy durability (i.e.: study secondary endpoint), follow up will also be completed at the 6 and 12 months post treatment.

The following evaluations should be performed at Month 6 and 12:

- Review of medications
- Physical exam
- Neurological status
- CRST, QUEST and PHQ-9-depression questionnaires should be completed by the subject
  - NOTE:

    At any follow up visit, any subject who scores ≥ 20 on the PHQ-9 questionnaire, the subject should be referred out for psychiatric evaluation. The Columbia Suicide Severity Rating Scale, or like, may be used. Should there be a psychiatric treatment, then this psychiatric treatment should be recorded as an adverse event. A Copy of the psychiatric evaluation should become part of the CRF information as part of the adverse event outcomes and description. The investigator will evaluate whether the subject may continue participation in the study, or should be exited from the study for treatment
- Adverse events
- At Month 12 (ONLY), all subjects should undergo the following MR exams:
  - T2 weighted imaging

4.2 Study Requirements and Visit Schedule

The table below summarizes the study visit schedule and procedures.

The study visits are as follows:

- Pre-treatment:

Baseline: 1 Month ± 7 days post screening

- Post-treatment:

1 Week (i.e. 7 days) ± 3 days, 1 Month ± 7 days, 3 Month ± 14 days (or 2 weeks), 6 Months ± 21 days (or 3 weeks), 12 Months ± 1 Month.

Table 4.1.2.3.1—1 Summary of Study Schedules and Measurements									
	**Screening**	**Baseline Assessment***	**Treatment**	**1 Day**	**1 Week**	**1 Month**	**3 Month**	**6 Month**	**12 Month**
**Consent**	**X**								
**Eligibility Evaluation with labs**	**X**	**X**							
**Medications**	**X**	**X**	**X**	**X**	**X**	**X**	**X**	**X**	**X**
**30 day meds stabilization**		**X**							
**Medical History**	**X**								
**Physical Exam**	**X**	**X**		**X**	**X**	**X**	**X**	**X**	**X**
**Neurological status**	**X**		**X**	**X**	**X**	**X**	**X**	**X**	**X**
**CRST**	**X**					**X**	**X**	**X**	**X**
**QOL (QUEST)**	**X**	**X**				**X**	**X**	**X**	**X**
**PHQ-9**	**X**					**X**	**X**	**X**	**X**
**CT**	**X**								
**MR**		**X**	**X**						**X**
**Treatment**			**X**						
**Adverse Events**			**X**	**X**	**X**	**X**	**X**	**X**	**X**
Forms “UB-04 (In Patient subjects) or CMS-1500 (Out Patient subjects)		**X**	**X**	**X**	**X**	**X**	**X**	**X**	**X**
**Exit Form**									**X**

5 DATA ANALYSIS PLAN

5.1 Safety

Adverse events will be recorded and categorized according to severity, relationship to procedure and relationship to device. All AEs will be assessed for their relationship to the study device or procedure. Standard Code of Federal Regulation definitions for Serious Adverse Events (SAEs) and Unanticipated Adverse Device Effects (UADEs) will be used in assessment of AEs.

5.2 Efficacy

Primary effectiveness will be evaluated using validated scores; the Clinical Rating Scale for Tremors (CRST) for the ET patients, based upon patients where unilateral ExAblate thalamotomy was attempted (i.e., Intent-to-Treat analysis). Efficacy is defined as a reduction in contralateral tremor of ET at 3-months post-treatment.

5.3 Subject Health Status

The results from the physical and neurological exams will be recorded in the CRFs and will be analyzed.

5.4 Statistical Considerations and Sample Size

.

This continued access, open-label, single-arm study will continue to enroll subjects from the time that enrollment on the pivotal study is completed until the FDA has rendered its decision on the PMA submission for ET002 – IDE # G120246. The centers that were part of the pivotal study and additional centers that have expressed an interest in this technique of thalamotomy may be included as recruiting centers. All data that were part of this study will be collected and reported to FDA during the PMA review. For this particular study, the total number of subjects for this ET002-Continued Access would be up to 100 subjects at up to 10 sites.

5.5 Statistical Analysis Plan.

The statistical analyses of this Continued Access ET002-CA study will be performed according to the Statistical Analyses Plan ("SAP") of the original study (ET002 under IDE # G120246) adjusted to "single arm" analyses.

5.6 Subject Confidentiality

Subject confidentiality will be maintained throughout this study, including all publications. Data collected and entered into the CRFs are the property of the study

sponsor. Representatives from the study sponsor or authorized sponsor representatives, the Institutional Review Board [86], Ethics Committee or other regulatory bodies may receive copies of the study records and may review medical records related to the study.

6 RISK ANALYSIS

Worldwide, over 8000 treatments have been performed to date with the MR guided FUS ExAblate body system. Risk analysis for InSightec ExAblate systems/clinical investigations has been conducted as part of previously approved FDA IDE submissions (G930140, G990151, G990184, G990201, G000203, G010225, G020001, G020182, G050177, and G060023, G070022, G080009, G080206, G100127, G100169, P040003 and subsequent supplements, and P110039). This data has been re-examined by the study sponsor and it has been concluded that this risk analysis has limited applicability to the proposed clinical investigation. The key consideration here is the fact that this proposed study is conducted with an ExAblate transcranial system that is completely different from the body system. This is system is referred to internally as the Brain system. However, in principle, the body and brain systems have the same purpose namely to coagulate soft tissue within the body by means of MR guided high intensity focused ultrasound. Additional risks, new and unique to this study are presented in **Section-**6.2**.**

The potential risks described below will be explained to the subject in the informed consent process.

6.1 General Procedure Related Risks

6.1.1 <u>Risk of Magnetic Resonance Scanners:</u>

MRI has no known deleterious biological effects in subjects with no contraindications. The incidence of claustrophobia during MRI examinations is approximately 10-15%. All subjects exhibiting claustrophobia will be exited from the study.

6.1.2 <u>Risk of Intravenous (IV) Catheter:</u>

There is a potential risk from the IV catheter used during the procedure. Participants can expect a small amount of pain and/or bleeding/bruising at the IV site. There is a small risk of infection. This procedure will follow the 'standard of care' at the study site.

6.1.3 <u>Risk of Urinary Catheter:</u>

There is a potential risk if a urinary catheter is used during the treatment. Participant may expect varying levels of Urinary Tract Infection due to the use of the urinary catheter. In a different study for the ExAblate treatment of uterine fibroid (Pivotal study under IDE G020001 that lead to PMA approval under PMA # P040003), the incidence of this risk was found not to exceed 3.7%. This procedure will follow the "standard of care" at the study site.

6.1.4 <u>Risk of Stereotaxic Head Frame:</u>

In this study, the Integra Radionics frame will be used for all treatments. The main part of this frame is a metal based ring that serves as a base for the rest of the fixation components. This frame comes with non-metallic posts mounts. Into the non-metallic post mounts, accompanying nylon encapsulated screws with a metallic tip are fitted through the posts. Only the metal tip of the screw is in contact with the patient. The risk of any skin event at or in the vicinity of the metallic tips is mitigated by the fact that the screws outer shell are out of nylon, and therefore eliminates any electrical conductivity to the skin. This is further mitigated by the fact that these screws are mounted on the non-metallic posts that links them to the metallic ring. Hence, the Integra frame is actually manufactured and sold with built in mitigating steps aimed at reducing / eliminating skin event risks. When the full set up is completed, the metallic ring is NOT in contact with the patient.

6.1.5 Risks Incidental to the ExAblate Treatment

There is a potential risk to the subject of deep venous thrombosis from lying in a supine position for 3 to 4 hours, but this risk is substantially mitigated by the avoidance of sedative medications. The risk to the subject from lying still for this treatment should be no greater than that of lying still for any other reason. Subjects will be provided compression stockings, as described above (Section 4.3), for use during treatment and are able to move their lower extremities during the treatment. DVT screening will be performed at the discretion of the primary treatment team for any subject deemed to be at a high risk for thrombosis.

There is a risk that the subject may experience a sore neck or back, or discomfort / fatigue from lying in the same position for a long time during the treatment.

6.1.6 Risks Associated with the ExAblate Treatment

All efforts are undertaken to reduce risk related to the ExAblate treatment. The following mitigations are in place for all procedures:

1. The procedure is performed in the MR scanner. During the treatment MR images will be acquired. Using specific scanning sequences and a rapid post-processing program, changes in temperature can be detected, and a thermal map of the brain generated. This thermal map will reveal any potentially dangerous elevations in temperature.
2. Study personnel can regularly assess cognitive and motor function throughout the procedure (i.e.: after each sonication) as well as general neurological function. This will help to reveal any indication that tissue damage may be occurring along the beam paths.
3. Subjects are asked to relay all sensations (i.e., disorientation/dizziness, pain, numbness, etc) occurring during the procedure to the circulating staff so that adjustments can be made to accommodate modifications to the treatment plan or extend time between sonications.

4. MR-compatible pulse oximeter, blood pressure cuff, and EKG monitor will be monitored throughout the procedure. This information will permit detection of tissue damage, edema, or bleeding, if brain or blood vessels along the beam paths are injured by heat.
5. The subject, Subject Monitor and Assessor, and the neurosurgeon will each have a stop sonication button that can instantaneously interrupt the energy delivery at any time. The subject is given a stop sonication button in case aberrant tissue heating causes any compromise to speech, word finding, or other communication difficulties. The subject will be instructed prior to the proceeding that they should use the stop sonication button any time they feel excessive pain, discomfort, disorientation or any other unusual sensation. The neurosurgeon, Subject Monitor and Assessor, have stop sonication buttons so that if there is any sign of neurological change, the energy delivery can be immediately stopped and the subject carefully evaluated. Temporary interruption of energy delivery will in no way compromise the potential for therapeutic benefit to the subject. Following subject evaluation treatment can resume without delay

The following risks may be associated with the ExAblate Transcranial thermal ablation procedure:

1. <u>Pain and/or discomfort</u> – There is a risk of discomfort to the subject caused by heating of tissue. Focused ultrasound therapy involves precisely controlled pulses of thermal energy resulting in tissue coagulation (typically 55-65°C for several seconds) in small tissue volumes. This induces thermal coagulation of the targeted soft tissue. The energy intensity at the level of the skin away from the pin sites is quite low and sonication through scar tissue should be avoided.. The subject may experience both a cold sensation from the active cooling circulation of water within the rubber diaphragm as well as a rise in temperature in the skull which should be kept to below pain level by the active cooling. Because the focal point of the beam will be > 2.5 cm from the dura and there are no pain receptors in the brain, there should be no pain associated with ablation, itself.

   The subject will be in constant verbal contact with the neurosurgeon and appropriate action can be taken in the event that a subject does experience pain discomfort. Remedies could involve lowering energy levels, or increasing the time interval between consecutive treatment pulses. The subject also has the ability to abort the sonication at any time by activating a handheld cut off circuit (i.e., stop sonication button).

   See also Risk 9c regarding heating of the dura mater and the meninges.
2. <u>Headache</u> – The subject may experience a headache during or after the procedure which may be related to the pin site, slight swelling around the ablated brain tissue, or a reaction to the contrast media and should be transient.

3. Disorientation, light-headedness, dizziness, unsteadiness, nausea/vomiting: There is a potential risk that the subject will have sensations of light-headedness, dizziness, unsteadiness or even nausea/vomiting. Based on the current data, these events should be transient and resolve within a day or so.

4. Occurrences of transient numbness, tingling, eye twitch, hyperesthesia and/or paresthesia of the scalp, face and upper extremity have been reported in subjects undergoing Vim ablation for essential tremor. These generally are transient are resolved within minutes to one day. One paresthesia lasted for 4 days; one scalp numbness lasted 2.5 months.

5. BBB disruption, edema, swelling, hemorrhage or stroke: There is a potential risk of hemorrhage during ExAblate procedure

    a. At the focal point or targeted area - In ExAblate thermal ablation, the high temperature at the focal point results in immediate protein denaturation and coagulative necrosis. This should be expected to rapidly stop any bleeding that might occur in the capillary bed and within small vessels.

    b. Outside and remote to the targeted area.. Alternatively, there could be a disruption of the Blood Brain Barrier (BBB) which would allow blood to seep out of the cranial blood vessels into the brain tissue. These events may theoretically occur due to heating effects (i.e. secondary hot spots) and or to the pressure wave of the ultrasound beam. To address the risks due to pressure waves of the ultrasound beam path, the system has been designed to be well below the “pressure wave threshold” that may trigger events of this nature.

In all cases, thermal and regular imaging will be continuously assessed during the procedure. Furthermore, at the end of the ExAblate procedure, a contrast (Gadolinium) enhanced MR scan is performed to assess the blood flow within the treated area and adjacent tissue. This exam constitutes an independent treatment assessment tool that provides further information on blood flow and can serve as final check of the overall tissue status and alert the physician to any significant edema, hemorrhage, and any other significant BBB disruption effect(s). Finally, the subject(s) is continuously monitored during the ablation procedure for any change in condition.

6. Imprecise focal point and ablation of tissue outside of target. There is a risk of imprecise targeting of the focal point, and ablation of an area of tissue outside the planned treatment volume. If this occurred it is possible that serious neurological deficit or even death could result. To limit the risk of this occurring, the treatment process includes a mandatory verification step that requires the operator to first check the alignment of the subject anatomy, the focal point of the transducer and the MR imaging system. This procedure, done while the subject is in position for treatment, uses a very low energy sonication to confirm of the alignment of the

focal point and the targeted treatment point in all three axes. For each sonication delivered during treatment, the operator gets continuous feedback on the position of the intended treatment point superimposed on the thermal dosimetry image and can make corrections where required.

At any point in the treatment process this low-power verification of the localization may be repeated prior to full power sonication.

7. Subject movement during the procedure that causes focal point to move/change location – There is a risk associated with subject motion during a sonication or between sonications. This could cause a movement of the tissue relative to the planned treatment volume on the system, and in extreme cases could result in the treatment of a point outside the planned treatment volume. Also, because the skull functions as a defocusing lens, the phase correction map computed for the target spot will become ineffective if the subject moves. To prevent or minimize this risk, there are several precautions taken to prevent motion, and to detect it, if it occurs:

    a. During subject positioning every effort will be made to make the subject comfortable and the subject will be educated as to the importance of maintaining their position during the treatment.

    b. The subject will be placed in a head immobilization unit based on a stereotactic frame. This technology has been effective in preventing movement in stereotactic neurosurgery, and has been adapted and modified to the specification of the ExAblate. The transducer and the immobilizer are locked together so that the transducer moves with the frame.

    c. The system is designed to detect motion and will abort a sonication if motion occurs during the sonication and will indicate that motion has been detected prior to the next sonication being performed.

    d. One or more members of the medical team will be in the room throughout the sonication to monitor the subjects' medical status and comfort. Hence, subject motion will also be monitored.

8. Risk of cavitation - There is a risk of cavitation in the tissue at the focal point. Cavitation is the collapse of rapidly developed gas bubbles at the focal point due an extreme intensity of ultrasound excitation. This rapid collapse could cause high pressure, shock waves, and high temperatures. However, we believe that through proper system design and careful selection of system operation envelope, there is a very minimal risk that cavitation could occur during a treatment, even in the event of user error. We have developed an automated treatment planner that takes as its input the targeted ablation area depth in tissue, focal volume and tissue absorption, and based on pre-set safe operating limits selects sonication parameters that will keep the intensity of ultrasound excitation well below the intensity levels that could cause cavitation.

9. <u>Risk related to sonication pathway</u> - There is the risk that the tissue along the path to the target (scalp, skull, dura, brain, etc) could become heated to the point where tissue-damage might occur which could result in significant neurological damage or even death. This heating could be caused by direct improper treatment targeting, irregularities on the skin surface (e.g.: scars), treatment of a volume of tissue too close to the skin or bone, energy absorption by the bone, or the conduction of sufficient heat to cause a burn. The heating in the energy pass zone is always monitored and an additional cooling time can be administered when elevated temperatures are detected. Below are some possible risks related to the sonication pathway through different tissues in the head:.

    a. . **Skin burn** is a potential risk due to improper acoustic coupling. There have been no cases of skin burn in all ExAblate transcranial treatments to date. The treatment set-up process includes filling the gap between the ultrasound transducer and the skull with a water-filled membrane to provide acoustic coupling. There is a possibility of small air bubbles remaining attached to the skin. These could cause a small focal hot spot and cause local pain or a burn to the scalp. The active cooling mechanism unique to this system is designed to reduce the risk of skin burns and improve subject comfort. In previous studies, ExAblate treatments have caused burns of the skin using the ExAblate Body system. To minimize this risk, the scalp will be carefully shaved, and scars or other irregularities (e.g. eczema) will be kept outside the treatment pathway. Subjects with remarkable atrophy and poor healing capacity of the scalp (> 30% of the skull area traversed by the sonication pathway) will be excluded from this study

       The subject will be in constant verbal contact with the neurosurgeon and appropriate action can be taken in the event that a subject does experience pain discomfort. Remedies could involve lowering energy levels, or increasing the time interval between consecutive treatment pulses. The subject also has the ability to abort the sonication at any time by activating a handheld cut off circuit (i.e., stop sonication button).

    b. **Skull and air-filled spaces:** In the treatment planning, air-filled spaces (frontal, ethmoid, sphenoid sinus, mastoid) inside the skull are identified in bone window CT images and kept outside the pathway. Should the operator not identify all air filled cavities on the treatment path, sonication through these areas could cause heating and result in burn of the tissue lining the cavity. Operators are trained to avoid these sonication pathways by marking their existence on the CT scans.

       Other irregularities of the skull, which might scatter the acoustic energy, are compensated for in the system. Skull may become heated by absorbing more acoustic energy than normal soft tissue. The skull cannot sense pain but the overlying soft tissues may sense pain if the bone becomes heated.

MRI thermometry at 1.5 T is able to detect changes of ±3 Celsius in soft tissues [86]. Possible heat transfer from the skull bone to the brain by successive sonications is monitored by MRI thermometry of the cortex and white matter. The sonication duration and energy levels, and the cooling times between the sonications are adjusted so that the focus in the target tissue is heated while allowing other tissue to cool down between sonications. Local bone damage is unlikely because the active cooling mechanism system is designed to keep the bone temperature below a temperature that can damage it. Based on the data acquired to date and reviewed by FDA under G020182/S04, the average temperature rise at the skull level ranges between 1° to 5° Celsius. Hence, this active cooling strategy should continue to provide the safety needed.

c. **Dura, meningeal arteries and venous sinuses:** The dura adjacent to the skull may absorb heat if the bone becomes heated. Dura itself may sense pain and the main branches of the arteries are sensitive to heat. The meningeal arteries can generally be avoided in the treatment planning as their grooves in the skull are visible in 3D-CT. Local necrosis of the dura is unlikely, and were it to happen, it would not cause cerebrospinal fluid leakage. The venous sinuses between the two leaves of the dura, the sagittal sinus, the straight sinus and the transverse sinus may be in the sonication pathway. Their heating will be avoided by the active cooling sub system. The sigmoid sinus and the cavernous sinus will be kept outside the pathway due to their proximity to the skull base and cranial nerves, respectively.

d. **Subarachnoid space:** Cerebrospinal fluid in the thin subarachnoid space between the dura and the cortex could possibly transfer heat from bone to the cortex. There is no specific risk to the CSF itself becoming heated. Because it can flow within the subarachnoid space, this can serve as another mechanism to prevent local hot spots next to the skull.

e. **Cortex:** In previous studies, ExAblate thermal ablation of deep foci in the rabbit brain there was no detectable heating of the cortex on MRI or evidence of opening of the blood-brain-barrier elsewhere than at the focus of the beam. Elevated temperature in the eloquent cortical areas (motor, sensory, visual, auditory, speech) might cause neurological deficits or seizures Temperatures in the eloquent areas will be monitored by MRI throughout the procedure and the cooling time between sonications will be increased if unacceptable thermal buildup is detected.

f. **Brain, cranial nerves and cerebral arteries:** In this study the treatment path will avoid cranial nerves and major cerebral arteries. Operators are trained to mark them on the MR for the purpose of avoidance during sonication.

g. **Target ablation and the adjacent brain tissue:** Thermal lesioning for the treatment of tremor through thalamotomy may carry some risks in particular to the internal capsule, ventral posterolateral nucleus of the thalamus. To mitigate these risks, the ExAblate treatment will be performed in small incremental sonications within the designated target volume in the un-anesthetized subject.. The subject will be examined by the Subject Assessor after each sonication for evidence of symptom suppression or clinical side effect. The titration of escalating focal sonications will start from well before evidence of thermal heat is detect by MR thermometry and will continue until clinical symptom suppression. This procedure will allow re-adjusting the targeting based on real time feedback from intra-procedure examination. This process is designed to enhance the procedure safety and minimize the potential adverse events that may be encountered in this study.

h. **Micro-calcification**: The subject population of this study may have some level of micro-calcification present in the brain tissue. Given calcium's higher absorption of ultrasound energy, its presence may create additional heating effect along the beam path. This risk is mitigated by utilizing the CT data (to localize the calcified areas) and the various tools of the ExAblate system to delineate these areas so that the beam is blocked from passing through these calcified areas.

i. **Secondary Hot Spots**: Theoretically speaking, there is a potential risk due to secondary hot spots that may occur along the beam path outside the focus. This has been reported in the literature for different types of transducer configurations using similar frequencies. The ExAblate system, with its unique, highly focused transducer configuration, was tested extensively using advanced simulations. The results of this work showed no evidence of significant hot spots away from the focal area. In any case, the real time thermal imaging feedback samples the entire field of view around the targeted tumor. These thermal images are displayed during the course of the energy delivery and therefore if any evidence of a secondary hot spot is observed, the treating physician will be able to utilize the ExAblate system "real-time" stop sonication button that instantaneously halts energy delivery. Importantly, the system is well equipped to handle it in real time and prior to incurring any tissue damage by shutting down the sonication prior to use of a Stop Sonication button being depressed..

10. <u>Neurological risks</u> Thermal "lesioning" in the brain may lead to heat transfer to immediately surrounding brain tissue or to hemorrhage. For a short period of time following the treatment, the surrounding tissue may be affected by inflammatory reaction. The length of the period cannot be predicted, but would be expected to resolve in 2-3 weeks based on our prior experience with sequential imaging in

focused ultrasound thalamotomy for ET. These mechanisms may cause transient local neurological deficits or symptoms. These complications are not typically permanent. If these symptoms are detected, medical management with dexamethasone and/or mannitol might be effective. In severe cases, a craniotomy can be performed to relieve increased intracranial pressure, and permanent neurological deficits or death could possibly result.

No benefit – The subject may receive no benefit from this procedure. While ablation of the Vim has generally been shown to be effective in tremor reduction, there is the chance that it will not be effective for the subject, or that the effect may not be permanent.

6.2 Anticipated Treatment Side Effects from ExAblate

All adverse events will be reported in the Case Report Forms (CRFs) and analyzed for their relationship to the treatment device, the procedure, and disease progression. Based on previous treatment experience, the following anticipated side effects have been identified as possible treatment related complications of ExAblate treatment. These can be classified into Non-significant and Significant Anticipated Treatment Side Effects based on their medical severity, additional treatment required, and long-term consequences for the subject.

Non-significant Anticipated Treatment Side Effects of ExAblate treatment are those that normally resolve without sequelae within 1-14 days of the treatment:

- minor pain from subject positioning or system interface (i.e. back pain, soreness in neck).
- transient fever: oral temperature >100.4^{o}F/38°C, lasting less than 24 hours
- minor (1° or 2°) skin burns less than 2 cm in diameter
- bruising of the skin along the treatment path
- increase in edema surrounding the treatment area as shown on MRI.
- Headache
- Dizziness, nausea or vomiting

Significant Anticipated Treatment Side Effects of ExAblate are those which may require medical treatment, may have sequelae, and for which time of resolution is not defined. The following side effects are thought to be improbable but their relative risk remains to be defined:

Scalp in the sonication pathway:

- skin burns (>2°) with ulceration of the skin
- scar formation
- Loss of sensation
- atrophy

Bone in the sonication pathway:

- bone necrosis

<u>Dura, venous sinuses, and cortical veins</u>

- subdural bleeding
- vein thrombosis
- seizures
- symptoms from disturbances of eloquent cortical areas (motor, sensory, auditory, visual, speech)

<u>Other brain tissue</u>

- necrosis of normal tissue due to incorrect targeting
- thermal damage to adjacent functional brain tissue (e.g.: optical tract)
- bleeding in the treated area
- cerebral infarction
- neurological deficits
- moderate or severe increase in cerebral edema as shown by MRI scans
- symptomatic increase of intracranial pressure
- death

<u>Cerebral arteries</u>

- bleeding
- coagulation thrombosis
- vasospasm
- death

The experience of Significant Anticipated Treatment Side Effects in ExAblate treatment in the breast and uterus have been less than 5% as shown in prior clinical experience. In brain treatments there is limited prior experience with the ExAblate Transcranial system and the probability of an adverse effect is unknown. It is the purpose of this study to gain experience that will allow us to evaluate the safety of ExAblate Transcranial system of TDPD (See also **Section-**6.1.6**.d**).

6.3 Adverse Reactions and Precautions

The subjects will be educated as to what to expect during the procedure and the importance of immediately communicating any problems, unusual symptoms, or discomfort, to the investigator during the treatment and throughout the follow-up period. Subjects will also be educated as to what sensations or perceptions could indicate that neurological damage may be starting to occur. They will be told to use their handheld stop sonication button if they perceive anything unusual may be happening so that they can be neurologically assessed. All adverse reactions occurring in this study will be recorded in the Case Report Forms. Each AE will be assessed for its cause (i.e.,

categorized as definitely, probably, possibly, or unrelated to the device, procedure or disease progression). Alternative treatments resulting from post-surgical changes in neurological status will be captured and reported.

6.4 Criteria for Removal from the Study

The investigator may withdraw subjects from the study as is deemed necessary or deemed to be in the best interests of the subject, such as,

- continued noncompliance with the protocol or study visits,
- severe illness or disability during the study for non-study issues,
- pursuit of subsequent alternative treatment for the same condition, or
- development of intolerable side effects where continued follow-up becomes too burdensome.

In addition, a subject may also chose to exit the study at any time, but will be strongly encouraged to participate in the follow-up visits for safety reasons (continued monitoring of subject safety).

6.5 Rules for Stopping the Study

This study may be stopped, if, in the opinion of the DSMB, serious risks to the health and welfare of subjects are observed that are directly related to the use of the device. The following scenarios would invoke this section of the protocol to stop the study while the cause of the safety concerns is being investigated and to determine relation of these safety events to the device, along with any possible mitigating steps s to protect patients from device harm. Either of the following scenarios would create cause for stopping the study to evaluate patient safety:

- Any death that is deemed to be caused by the ExAblate treatment
- 2 or more events of intracranial hemorrhage deemed to be caused by the ExAblate device
- Any other serious, related Unanticipated event that causes significant disability/ harm to the subject that is deemed to be the result of the ExAblate device.

Investigator(s) shall promptly report the occurrence of any such events to the Sponsor (i.e. InSightec) and the Sponsor shall promptly initiate review by the DSMB as well as reporting such event(s) to the FDA. Once the DSMB has had an initial assessment of an event of this type, it may recommend stopping the study in order to investigate fully the events and circumstances. This will be communicated to FDA and all IRBs and sites. With full cooperation of the treating investigator and the Sponsor, the DSMB will evaluate the circumstances of the event, determine relation of these events to the ExAblate, and whether any modifications to the protocol or the device must be made to improve safety. The Sponsor will keep FDA informed of the results of the DSMB evaluation and of any potential actions taken by the Sponsor. Once the review is

completed and any mitigations are in place, and the DSMB recommends re-opening the study with concurrence by the FDA, then then InSightec will communicate this with all sites and their IRBs to begin to re-open and continue the study.

6.6 Adverse Event Reporting

It is the responsibility of the investigator to document all AE's occurring during the course of the study. At each visit, the investigator will evaluate AE's. AE's not previously documented in the study will be recorded on the Adverse Event Log within the CRF. The nature of each event, date and time (when appropriate) of onset, outcome, frequency, maximum intensity, action taken, expectedness, and causal relationship will be recorded. AEs already documented in the CRF (i.e., at a previous assessment) and designated as 'ongoing', should be reviewed at subsequent visits as necessary. If these have resolved, the documentation in the CRF should be completed including an end date for the event.

Standard Code of Federal Regulation (CFR) definitions for Serious Adverse Events (SAEs) will be used for evaluation of adverse events.

***SAE** [§803.3(aa)(1)] is an injury or illness that:*

- *causes death*
- *is life threatening, even if temporary in nature;*
- *results in permanent impairment of a body function or permanent damage to a body structure; or*
- *necessitates medical or surgical intervention to preclude permanent impairment of a body function or permanent damage to a body structure.*

All AEs (related or unrelated) meeting the criteria for an SAE require notification of the sponsor and the reviewing IRB as soon as possible, with subsequent completion of additional paperwork provided by the sponsor fully documenting the course of the event, all treatments, and final outcome. Initial reporting of an SAE should be made to the sponsor no later than two (2) working days after the PI learns of the incident. AE's that do not affect the safety or overall well being of the subject, are mild/moderate in nature, are estimated to be temporary in duration even though the exact end date may not be determined *a priori* (e.g., eye twitch increased from baseline) may be presented and discussed with DSMB to determine their final classification status as a serious or non-serious adverse event.

Standard Code of Federal Regulation (CFR) definitions for Unanticipated Adverse Device Effects (UADEs) will be used for evaluation of this type of adverse event.

***UADE** [§812.3(s)] means any serious adverse event on health or safety or any life-threatening problem or death caused by, or associated with, a device, if that effect, problem, or death was not previously identified in nature, severity, or degree of incidence in the investigational plan or application (including a supplementary plan or application), or any other unanticipated serious problem associated with a device that relates to the rights, safety, or welfare of subjects.*

Any UADEs will be reported to the Sponsor and to the reviewing IRB as soon as possible. However, in no event must this report be made later than two (2) working days after the PI learns of the incident.

6.7 Data Safety Monitoring Board

A Data Safety Monitoring Board will be used to review all AE's on the study. Their role is to evaluate all AE's that occur throughout the study and determine if they are in fact related to the ExAblate, or some other cause. Investigators will monitor all treatments for any AE's, and consider the following questions for AEs in the Test Arm:

- *Was the adverse event serious?*
- *Was the adverse event life-threatening, caused a disability, required or prolonged hospitalization, or caused death?*
- *Was the adverse event device related?*
- *Was the adverse event unexpected?*
- *Is there an unreasonable risk in continuing the trial?*

Adverse Events meeting all the above conditions would require reporting to the FDA, stopping the study pending the results of further investigation, and FDA approval to re-start the study. Following the DSMB review of the event, and if in the opinion of the DSMB, a modification of the study protocol were necessary to provide adequate protection to future study participants, the modification would be implemented prior to reinitiating the investigation. Any such amendment would be reported to the IRB and FDA for their respective approvals to re-start the study as it is required by the applicable regulations.

All adverse events will be assessed for their relationship to the study device or procedure. Standard Code of Federal Regulation (CFR) definitions for SAEs and UADEs will be used in assessment of adverse events.

7 POTENTIAL BENEFITS

There may or may not be any benefit to participating in this study. This technique is still being investigated. It may provide some therapeutic value for subjects with few or no other options due to the great risk that would be involved in open resection. The symptoms may decrease and/or the quality of life of the subject may improve due to relief of symptoms. However, there is no guarantee that this procedure will reduce, eliminate symptoms, or otherwise treat the underlying disorder. Other subjects may benefit from this procedure in the future, if further trials prove it to be a safe and effective therapy.

8 MONITORING PLAN

Clinical Monitoring for this study will be managed by InSightec. The Clinical Monitor is qualified by training and experience to oversee the conduct of this study. The Clinical Monitor's responsibilities include maintaining regular contact with each investigational site through telephone contact and on-site visits, to ensure that:

- The trial is conducted according to FDA and GCP requirements;
- The trial is conducted according to InSightec internal SOPs
- The Investigational Plan is followed;
- Complete, timely, and accurate data are submitted;
- Problems with inconsistent or incomplete data are addressed;
- Complications and unanticipated adverse effects are reported to the Sponsor and the IRB;
- The site facilities will be monitored to stay adequate to meet the requirements of the study.

The Clinical Monitor will initiate the Study during an on-site visit and will continue to perform on-site monitoring visits as frequently as deemed necessary. The first monitoring visit will usually be made as soon as possible after enrollment has been initiated. At this visit and all monitoring visits, the Clinical Monitor will compare the data entered onto the CRFs with the hospital or clinical records (source documents). Source documentation must be available to substantiate proper informed consent procedures, adherence to protocol procedures, adequate reporting and follow-up of AEs, and device procedure information. Findings from the review of CRFs and source documents during a monitoring visit will be discussed with the PI. Completed paper or electronic CRFs will be reviewed prior to data closure at each visit. The dates of the monitoring visits will be recorded in a Log to be kept at the clinical site. During monitoring visits, the Sponsor expects that the study coordinator and the PI will be available, the source documentation will be available, and a suitable environment will be provided for review of Study related documents.

Sites should make every effort to contact all subjects for study follow-up to encourage visit compliance. Sites should keep a log of dates of attempted contact and results. After 3 unsuccessful attempts at contact (e.g., by telephone or email) and sending 1 certified letter to solicit their visit compliance a subject may be considered lost to follow-up.

Monitoring procedures will follow the Sponsor SOPs.

8.1 Electronic Data Capture (EDC)

Electronic CRFs (eCRFs) will be to capture protocol-specific information during the conduct of this study. This electronic data capture of the eCRFs is based on the Oracle Software system, and is designed, run and hosted by Sponsor (Haifa, Israel).

9 INVESTIGATOR RESPONSIBILITIES

The Principal Investigator will be required to sign the Investigator Agreement. All investigators will undergo extensive training on the protocol and operation of the ExAblate system, and provide documentation of their specialized training.

10 APPENDICES

Appendix – A: Informed Consent

11 REFERENCES

1. Hynynen, K., et al., *Noninvasive mr imaging-guided focal opening of the blood-brain barrier in rabbits.* Radiology, 2001. **220**(3): p. 640-6.
2. Hynynen, K., et al., *MR imaging-guided focused ultrasound surgery of fibroadenomas in the breast: a feasibility study.* Radiology, 2001. **219**(1): p. 176-85.
3. Cline, H.E., et al., *Focused US system for MR imaging-guided tumor ablation.* Radiology, 1995. **194**(3): p. 731-7.
4. Cline, H.E., et al., *MR-guided focused ultrasound surgery.* J Comput Assist Tomogr, 1992. **16**(6): p. 956-65.
5. Cline, H.E., et al., *Magnetic resonance-guided thermal surgery.* Magn Reson Med, 1993. **30**(1): p. 98-106.
6. Hynynen, K., et al., *MRI-guided noninvasive ultrasound surgery.* Med Phys, 1993. **20**(1): p. 107-15.
7. Bednarski, M. and e. al., *In vivo Target Specific delivery of macromolecular agents with MR-Guided focused ultrasound.* Radiology, 1997. **204**: p. 263.
8. Bharucha, N.E., et al., *Prevalence of essential tremor in the Parsi community of Bombay, India.* Arch Neurol, 1988. **45**(8): p. 907-8.
9. Haerer, A.F., D.W. Anderson, and B.S. Schoenberg, *Prevalence of essential tremor. Results from the Copiah County study.* Arch Neurol, 1982. **39**(12): p. 750-1.
10. Hornabrook, R.W. and J.T. Nagurney, *Essential tremor in Papua, New Guinea.* Brain, 1976. **99**(4): p. 659-72.
11. Rajput, A.H., et al., *Essential tremor in Rochester, Minnesota: a 45-year study.* J Neurol Neurosurg Psychiatry, 1984. **47**(5): p. 466-70.
12. Rautakorpi, I., et al., *Essential tremor in a Finnish population.* Acta Neurol Scand, 1982. **66**(1): p. 58-67.
13. Nguyen, H.V., et al., *Quality of life in a random sample of community dwelling older patients with essential tremor.* Acta Neurol Scand, 2007. **116**(5): p. 289-92.
14. Parisi, S.L., et al., *Functional mobility and postural control in essential tremor.* Arch Phys Med Rehabil, 2006. **87**(10): p. 1357-64.
15. Stolze, H., et al., *The gait disorder of advanced essential tremor.* Brain, 2001. **124**(Pt 11): p. 2278-86.
16. Bove, M., et al., *Posturographic analysis of balance control in patients with essential tremor.* Mov Disord, 2006. **21**(2): p. 192-8.
17. Lombardi, W.J., et al., *Cognitive deficits in patients with essential tremor.* Neurology, 2001. **57**(5): p. 785-90.
18. Lorenz, D., et al., *Quality of life and personality in essential tremor patients.* Mov Disord, 2006. **21**(8): p. 1114-8.

19. Woods, S.P., et al., *Executive dysfunction and neuropsychiatric symptoms predict lower health status in essential tremor.* Cogn Behav Neurol, 2008. **21**(1): p. 28-33.
20. Schneier, F.R., et al., *Characteristics of social phobia among persons with essential tremor.* J Clin Psychiatry, 2001. **62**(5): p. 367-72.
21. Louis, E.D., et al., *Correlates of functional disability in essential tremor.* Mov Disord, 2001. **16**(5): p. 914-20.
22. Heroux, M.E., et al., *Upper-extremity disability in essential tremor.* Arch Phys Med Rehabil, 2006. **87**(5): p. 661-70.
23. Zesiewicz, T.A., et al., *Practice parameter: therapies for essential tremor: report of the Quality Standards Subcommittee of the American Academy of Neurology.* Neurology, 2005. **64**(12): p. 2008-20.
24. Laitinen, L.V., *Brain targets in surgery for Parkinson's disease. Results of a survey of neurosurgeons.* J Neurosurg, 1985. **62**(3): p. 349-51.
25. Brodkey, J.A., et al., *Tremor cells in the human thalamus: differences among neurological disorders.* J Neurosurg, 2004. **101**(1): p. 43-7.
26. Hirai, T., et al., *The correlation between tremor characteristics and the predicted volume of effective lesions in stereotaxic nucleus ventralis intermedius thalamotomy.* Brain, 1983. **106 (Pt 4)**: p. 1001-18.
27. Pahwa, R., et al., *Comparison of thalamotomy to deep brain stimulation of the thalamus in essential tremor.* Mov Disord, 2001. **16**(1): p. 140-3.
28. Schuurman, P.R., et al., *A comparison of continuous thalamic stimulation and thalamotomy for suppression of severe tremor.* N Engl J Med, 2000. **342**(7): p. 461-8.
29. Tasker, R.R., *Deep brain stimulation is preferable to thalamotomy for tremor suppression.* Surg Neurol, 1998. **49**(2): p. 145-53; discussion 153-4.
30. Obwegeser, A.A., et al., *Quantitative and qualitative outcome measures after thalamic deep brain stimulation to treat disabling tremors.* Neurosurgery, 2001. **48**(2): p. 274-81; discussion 281-4.
31. Obwegeser, A.A., et al., *Thalamic stimulation for the treatment of midline tremors in essential tremor patients.* Neurology, 2000. **54**(12): p. 2342-4.
32. Putzke, J.D., et al., *Bilateral thalamic deep brain stimulation: midline tremor control.* J Neurol Neurosurg Psychiatry, 2005. **76**(5): p. 684-90.
33. Carpenter, M.A., et al., *Reduction in voice tremor under thalamic stimulation.* Neurology, 1998. **50**(3): p. 796-8.
34. Diamond, A. and J. Jankovic, *The effect of deep brain stimulation on quality of life in movement disorders.* J Neurol Neurosurg Psychiatry, 2005. **76**(9): p. 1188-93.
35. Fields, J.A., et al., *Neuropsychological and quality of life outcomes 12 months after unilateral thalamic stimulation for essential tremor.* J Neurol Neurosurg Psychiatry, 2003. **74**(3): p. 305-11.

36. Hariz, G.M., M. Lindberg, and A.T. Bergenheim, *Impact of thalamic deep brain stimulation on disability and health-related quality of life in patients with essential tremor.* J Neurol Neurosurg Psychiatry, 2002. **72**(1): p. 47-52.

37. Mohadjer, M., et al., *Long-term results of stereotaxy in the treatment of essential tremor.* Stereotact Funct Neurosurg, 1990. **54-55**: p. 125-9.

38. Sydow, O., et al., *Multicentre European study of thalamic stimulation in essential tremor: a six year follow up.* J Neurol Neurosurg Psychiatry, 2003. **74**(10): p. 1387-91.

39. Benabid, A.L., et al., *Long-term electrical inhibition of deep brain targets in movement disorders.* Mov Disord, 1998. **13 Suppl 3**: p. 119-25.

40. Hariz, M.I., et al., *Tolerance and tremor rebound following long-term chronic thalamic stimulation for Parkinsonian and essential tremor.* Stereotact Funct Neurosurg, 1999. **72**(2-4): p. 208-18.

41. Yamamoto, T., et al., *Deep brain stimulation for the treatment of parkinsonian, essential, and poststroke tremor: a suitable stimulation method and changes in effective stimulation intensity.* J Neurosurg, 2004. **101**(2): p. 201-9.

42. Clement, G.T. and K. Hynynen, *A non-invasive method for focusing ultrasound through the human skull.* Physics in Medicine & Biology, 2002. **47**(8): p. 1219-36.

43. Clement, G.T., et al., *A magnetic resonance imaging-compatible, large-scale array for trans-skull ultrasound surgery and therapy.* Journal of Ultrasound in Medicine, 2005. **24**(8): p. 1117-25.

44. Hynynen, K., et al., *Pre-clinical testing of a phased array ultrasound system for MRI-guided noninvasive surgery of the brain--a primate study.* European Journal of Radiology, 2006. **59**(2): p. 149-56.

45. Hynynen, K. and F.A. Jolesz, *Demonstration of potential noninvasive ultrasound brain therapy through an intact skull.* Ultrasound Med Biol, 1998. **24**(2): p. 275-83.

46. McDannold, N., et al., *MRI investigation of the threshold for thermally induced blood-brain barrier disruption and brain tissue damage in the rabbit brain.* Magn Reson Med, 2004. **51**(5): p. 913-23.

47. Cohen, Z.R., et al., *Magnetic resonance imaging-guided focused ultrasound for thermal ablation in the brain: a feasibility study in a swine model.* Neurosurgery, 2007. **60**(4): p. 593-600; discussion 600.

48. Ram, Z., et al., *Magnetic resonance imaging-guided, high-intensity focused ultrasound for brain tumor therapy.* Neurosurgery, 2006. **59**(5): p. 949-55; discussion 955-6.

49. Martin, E., et al., *Focused Ultrasound for Non-invasive Neurosurgery.* Annals of Neurology, 2009. **In press.**

50. Jolesz, F.A., A.R. Bleier, and R.S. Lauter, *Laser surgery benefits from guidance by MR.* Diagn Imaging (San Franc), 1990. **12**(9): p. 103-8.

51. Jolesz, F.A. and S.M. Blumenfeld, *Interventional use of magnetic resonance imaging.* Magn Reson Q, 1994. **10**(2): p. 85-96.

52. Jolesz, F.A. and N. McDannold, *Current status and future potential of MRI-guided focused ultrasound surgery.* Journal of Magnetic Resonance Imaging, 2008. **27**(2): p. 391-9.

53. Jolesz, F.A., et al., *Response to and control of destructive energy by magnetic resonance.* Invest Radiol, 1989. **24**(12): p. 1024-7.

54. Moonen, C.T., et al., *Thermal therapies in interventional MR imaging. Focused ultrasound.* Neuroimaging Clinics of North America. **11**(4): p. 737-47.

55. Salomir, R., et al., *Image-based control of the magnetic resonance imaging-guided focused ultrasound thermotherapy.* Topics in Magnetic Resonance Imaging, 2006. **17**(3): p. 139-51.

56. Kleiner-Fisman, G., et al., *Subthalamic nucleus deep brain stimulation for parkinson's disease after successful pallidotomy: clinical and electrophysiological observations.* Mov Disord, 2004. **19**(10): p. 1209-14.

57. White, D.N., *Neurosonology pioneers.* Ultrasound Med Biol, 1988. **14**(7): p. 541-61.

58. Aaslid, R., T.M. Markwalder, and H. Nornes, *Noninvasive transcranial Doppler ultrasound recording of flow velocity in basal cerebral arteries.* J Neurosurg, 1982. **57**(6): p. 769-74.

59. Fry, W.J., et al., *Production of focal destructive lesions in the central nervous system with ultrasound.* J. Neurosurg, 1954. **11**: p. 471-478.

60. Fry, W.J., et al., *Ultrasonically produced localized selective lesions in the central nervous system.* Am. J. Phys. Med, 1955. **34**: p. 413-423.

61. Lele, P.P., *Production of deep focal lesions by focused ultrasound--current status.* Ultrasonics, 1967. **5**: p. 105-12.

62. Fry, F.J., *Ultrasonic visualization of human brain structure.* Invest Radiol, 1970. **5**(2): p. 117-21.

63. Fry, F.J. and J.E. Barger, *Acoustical properties of the human skull.* J Acoust Soc Am, 1978. **63**(5): p. 1576-90.

64. Fry, F.J., S.A. Goss, and J.T. Patrick, *Transkull focal lesions in cat brain produced by ultrasound.* Journal of Neurosurgery, 1981. **54**(5): p. 659-63.

65. Lynn, J.G., et al., *A new method for the generation and use of focused ultrasound in experimental biology.* J. Gen. Physiol., 1942. **26**: p. 179-193.

66. Smith, S.W., et al., *Some Advances in Acoustic imaging through Skull.* Symposium on Biological Effects, 1997: p. 37-52.

67. Guthkelch, A.N., et al., *Treatment of malignant brain tumors with focused ultrasound hyperthermia and radiation: results of a phase I trial.* J Neurooncol, 1991. **10**(3): p. 271-84.

68. Clement, G.T., et al., *A hemisphere array for non-invasive ultrasound brain therapy and surgery.* Phys Med Biol, 2000. **45**(12): p. 3707-19.

69. Clement, G.T., J. White, and K. Hynynen, *Investigation of a large-area phased array for focused ultrasound surgery through the skull.* Phys Med Biol, 2000. **45**(4): p. 1071-83.

70. Spiegel, J., et al., *Transient dystonia following magnetic resonance imaging in a patient with deep brain stimulation electrodes for the treatment of Parkinson disease. Case report.* J Neurosurg, 2003. **99**(4): p. 772-4.

71. Vykhodtseva, N., et al., *MRI detection of the thermal effects of focused ultrasound on the brain.* Ultrasound Med Biol, 2000. **26**(5): p. 871-80.

72. Niranjan, A., et al., *A comparison of surgical approaches for the management of tremor: radiofrequency thalamotomy, gamma knife thalamotomy and thalamic stimulation.* Stereotact Funct Neurosurg, 1999. **72**(2-4): p. 178-84.

73. Sansur, C.A., et al., *Incidence of symptomatic hemorrhage after stereotactic electrode placement.* J Neurosurg, 2007. **107**(5): p. 998-1003.

74. Deogaonkar, M., et al., *Clinical problem solving: finding the target.* Neurosurgery, 2007. **61**(4): p. 815-24; discussion 824-5.

75. Elias, W.J., K.M. Fu, and R.C. Frysinger, *Cortical and subcortical brain shift during stereotactic procedures.* J Neurosurg, 2007. **107**(5): p. 983-8.

76. Xiaowu, H., et al., *Risks of intracranial hemorrhage in patients with Parkinson's disease receiving deep brain stimulation and ablation.* Parkinsonism Relat Disord. **16**(2): p. 96-100.

77. Pahwa, R., et al., *Long-term evaluation of deep brain stimulation of the thalamus.* J Neurosurg, 2006. **104**(4): p. 506-12.

78. Duma, C.M., et al., *Gamma knife radiosurgery for thalamotomy in parkinsonian tremor: a five-year experience.* J Neurosurg, 1998. **88**(6): p. 1044-9.

79. Ohye, C. and T. Shibazaki, *Treatment of functional disorders with gamma knife thalamotomy.* Prog Neurol Surg, 2009. **22**: p. 170-81.

80. Jagannathan, J., et al., *High-Intensity Focused Ultrasound Surgery of the Brain: Part 1-a Historical Perspective with Modern Applications.* Neurosurgery, 2009. **64**(2): p. 201-211.

81. Gianfelice, D., et al., *MR imaging-guided focused US ablation of breast cancer: histopathologic assessment of effectiveness-- initial experience.* Radiology, 2003. **227**(3): p. 849-55.

82. Gianfelice, D., et al., *MR imaging-guided focused ultrasound surgery of breast cancer: correlation of dynamic contrast-enhanced MRI with histopathologic findings.* Breast Cancer Res Treat, 2003. **82**(2): p. 93-101.

83. Gianfelice, D., et al., *Feasibility of magnetic resonance imaging-guided focused ultrasound surgery as an adjunct to tamoxifen therapy in high-risk surgical patients with breast carcinoma.* J Vasc Interv Radiol, 2003. **14**(10): p. 1275-82.

84. Gianfelice, D., et al., *Palliative treatment of painful bone metastases with MR imaging--guided focused ultrasound.* Radiology, 2008. **249**(1): p. 355-63.

85. Stacy, M.A., et al., *Assessment of interrater and intrarater reliability of the Fahn-Tolosa-Marin Tremor Rating Scale in essential tremor.* Mov Disord, 2007. **22**(6): p. 833-8.

86. McDannold, et al., *MRI evaluation of thermal ablation of tumors with focused ultrasound.* J Magn Reson Imaging, 1998. **8**(1): p. 91-100.
