# Supplementary material for: Unilateral magnetic resonance-guided focused ultrasound for medication-refractory essential tremor: 5-year continued access study
Source: Front Neurol. 2025 Oct 22;16:1659203. doi: 10.3389/fneur.2025.1659203 (PMC12587677; doi:10.3389/fneur.2025.1659203)
Supplement: Supplementary file 4 [file Data_Sheet_3.pdf]

Clinical Protocol

**Version Date**	**January 21st , 2015**
**Amendment No.**	
**Protocol Number**	**ET002-LTF**
**IDE Number**	**G120246**
**Protocol Title**	**A Long-term Observational Follow-up Study of Medication Refractory Essential Tremor Subjects Treated with ExAblate Transcranial MRgFUS Thalamotomy under IDE G120246**

Approvals:

**Site**	**Sponsor**
[signature] 5/07/15<br>Principal Investigator Signature Date	[signature] 2/27/15<br>Nadir Alikacem, PhD, Date<br>VP Global Regulatory Affairs and CRO<br>InSightec

A Long-term Observational Follow-up Study of Medication Refractory Essential Tremor Subjects Treated with ExAblate Transcranial MRgFUS Thalamotomy in Clinical Trials

The Objective of this observational follow-up study is to collect long-term information regarding the Safety and Efficacy of medication-refractory Essential Tremor subjects treated with the ExAblate Transcranial System under IDE#120246.

The Indications for Use claim for this system is as follows: Treatment of medication-refractory tremor in patients with essential tremor.

Protocol Number: ET002-LTF

InSightec

4851 LBJ Freeway

Suite 400

Dallas, Texas 75244

Table Of Content

1. 1. BACKGROUND and SIGNIFICANCE ***5***

1.1 Movement Disorder in Essential Disorders Patients ***5***

1.2 ExAblate Transcranial MRgFUS System. ***5***

2 OBJECTIVES ***6***

2.1 Primary Endpoints ***6***

2.1.1 Safety 6

2.1.2 Effectiveness 6

2.1.2.1 Efficacy Assessments 6

2.2 Study Hypothesis ***7***

2.3 Case Report Form Data ***7***

3 DESCRIPTION OF PATIENT POPULATION ***7***

3.1 Patient Selection ***7***

3.1.1 Inclusion Criteria 7

3.1.2 Exclusion Criteria 7

4 INVESTIGATIONAL PLAN ***7***

4.1 Follow Up Periods Years 2 – 5 Post Treatment ***7***

4.2 Exit from the study for reason of alternative treatment ***8***

4.3 Study Requirements and Visit Schedule ***8***

5 DATA ANALYSIS PLAN ***9***

5.1 Safety ***9***

5.2 Efficacy ***9***

5.3 Subject Health Status ***9***

5.4 Statistical Considerations and Sample Size ***9***

5.5 Missing Data ***10***

5.6 Statistical Analysis Plan. ***10***

5.7 Subject Confidentiality ***10***

6 RISK ANALYSIS ***10***

6.1 Criteria for Removal from the Study ***11***

6.2 Data Safety Monitoring Board ***11***

7 POTENTIAL BENEFITS ***12***

8 MONITORING PLAN ***12***

8.1 Electronic Data Capture (EDC) ***13***

9 INVESTIGATOR RESPONSIBILITIES ***13***

10 REFERENCES 14

1.

1. BACKGROUND and SIGNIFICANCE

1.1 Movement Disorder in Essential Disorders Patients

Essential tremor is the most common movement disorder with an estimated prevalence between 0.3% and 5.6% [1-5]. Recent epidemiological reports[1] indicate that prevalence across 19 countries (pooled) was 0.9%, but increased to 4.6% among those 65 years and as high as 21.7% in those aged 95 and older. The condition is a genetically inherited disorder with a child of an ET person having a 50% chance of inheriting a gene causing the condition. Approximately 50-70% of people diagnosed with ET have a positive family history for the condition. Caucasians are 5 times more likely to report physician diagnoses of ET than are African Americans; Hispanics have a rate between them. Gender predisposition as reported in various articles is variable depending upon the author's population.

ET is a slowly progressive neurological disorder characterized by a tremor of the arms or hands that occurs during voluntary movements (intention tremor), such as eating/drinking and writing. The tremor may also present in the head (neck) and jaw and may affect voice. The disease may present in the teens or in the 40-50 age range. Generally, tremor begins in the arms and then spreads to these other regions in selected patients. Other types of tremor may also present, including postural tremor of the outstretched arms, and intentional tremor (below 5 Hz) and rest tremor of the arms. The amplitude of an intention tremor increases as an extremity approaches the endpoint of deliberate and visually guided movement (hence the name intention tremor). An intention tremor is usually perpendicular to the direction of movement. An intention tremor causes the person to overshoot or undershoot their target (dysmetria).

1.2 ExAblate Transcranial MRgFUS System.

The non-invasive high-intensity focused ultrasound has been coupled with high resolution MRI to provide precise, consistent treatments that can be monitored in real-time. The development of phased array transducers allows for tightly focused treatment volumes and for the ability to compensate for distortions by tissue heterogeneity [6-8]. The landmark advance in the ExAblate TcMRgFUS for neurosurgeons occurred as the ability to sonicate through the intact cranium was achieved with phased array transducers and acoustic modeling using CT reconstructions of the skull [6-10]. By coupling focused ultrasound technology with MRI, the ExAblate system allows detailed treatment plans to be performed and real time intra-procedure monitoring [11]. Standard MR sequences have been shown to reliably predict tissue damage during thermal lesioning with ultrasound [11, 12]. We anticipate that the ExAblate TcMRgFUS non-invasive thermal lesioning is safe and will provide several years of benefit through reduction of contralateral motor symptoms and potential medication side effects in ET subjects.

[1] http://www.medmerits.com/index.php/article/epidemiology_of_movement_disorders/P4

2 OBJECTIVES

The objective of this clinical trial is to follow, observationally, the medication-refractory Essential Tremor subjects who underwent ExAblate Transcranial thalamotomy under IDE# G120246 to capture long-term safety and effectiveness out to Year 5.

Safety: To evaluate long-term incidence and severity of adverse events (AE/AEs) associated with ExAblate Transcranial MRgFUS treatment of medication-refractory ET

Effectiveness: To collect long term effectiveness and quality of life of the ExAblate Transcranial MRgFUS treatment of medication-refractory Essential tremor ("ET").

This study is designed as a long-term prospective, observational clinical trial to follow device related safety, and long term effectiveness (CRST) and quality of life (QUEST) for subjects previously treated with ExAblate Transcranial under IDE#120246.

Previously treated subjects under original IDE (IDE # G120246) will be followed from their 1 year visit on previous clinical trials through 5 years post-treatment. Patients have already been consented on the pivotal and continued access studies through Year 5 follow-up. The purpose of this protocol is to set the long-term evaluations (Year 2 – Year 5) to be performed at annually scheduled follow-up visits.

2.1 Primary Endpoints

2.1.1 Safety

Safety of ExAblate will be determined by an evaluation of the incidence and severity of device / treatment related complications from the treatment day visit through ALL study follow ups through Year 5. Adverse events (type, frequency, severity) are expected to be similar to those of previous studies using ExAblate TcMRgFUS for Thalamotomy

Recorded adverse events will be reported and categorized by investigators as definitely, probably, possibly, or unrelated to the device or procedure. Events which are not considered to be possibly or probably caused by the device are not required to be reported here.

2.1.2 Effectiveness

Primary effectiveness will be assessed using the CRST as scored by the site neurologist.

Secondary effectiveness will be followed using the QUEST quality of life patient outcomes questionnaire.

2.1.2.1 Efficacy Assessments

Tremor symptom severity will be assessed using the CRST as scored by the site neurologist at each annual follow-up visit Year 2 through Year 5.. The CRST is a validated clinical instrument used to assess tremor symptom severity.

Quality of life will be evaluated using the QUEST assessment to assess durability (as measured by QUEST upper arm extremity questions) of the procedure. The QUEST is an ET specific assessment of quality of life changes associated with ET. Tröster *et al*., 2005, developed QUEST as a clinical tool for correlating changes in 30 aspects of tremor severity, social and personal disability, and perception of health. An independent validation study of the QUEST performed by Martinez-Martin *et al*. concluded that most of the psychometric parameters were found to be satisfactory in their ability to assess the impact of ET on the patients' quality of life.

2.2 Study Hypothesis

The purpose of this study is to continue to follow the ExAblate-treated medication-refractory ET subjects for long-term safety and effectiveness of MRI-guided focused ultrasound thermal ablation out through 5 years.

2.3 Case Report Form Data

The study data will be collected electronically. This electronic data capture (EDC) system complies with the current guidance of 21 CFR Part 11, Electronic Records and Signatures.

3 DESCRIPTION OF PATIENT POPULATION

3.1 Patient Selection

3.1.1 Inclusion Criteria

1. Subjects who have been treated with ExAblate for medication-refractory ET under previous clinical trials.

3.1.2 <u>Exclusion Criteria</u>

1. Subjects who have had a subsequent intervention for ET on their treated side.

4 INVESTIGATIONAL PLAN

4.1 Follow Up Periods Years 2 – 5 Post Treatment

The treatment long term safety and effectiveness follow up will be completed annually from Year 2 through Year 5 post treatment under this protocol. It should be noted that at these visits, the CRST assessments will be performed and scored by the site neurologist.

The following evaluations should be performed at Year 2, Year 3, Year 4 and Year 5:

- Review of medications

- Physical exam
- CRST – Assessed by site evaluator
- QUEST questionnaire should be completed by the subject
- Adverse events

4.2 Exit from the study for reason of alternative treatment

In this study, subjects who opt for alternative treatments for Essential Tremor (not including medication change) at any point in the follow-up period will be exited from the study after completing the required study examinations. The last set of evaluations prior to alternative therapy is considered the last study visit. The reason(s) for study exit will be noted on the Case Report Forms. No analyses of post alternative treatment changes are planned.

4.3 Study Requirements and Visit Schedule

The table below summarizes the study visit schedule and procedures.

The study visits are as follows:

Year 2 ± 2 Month;

Year 3 ± 2 Month,

Year 4 ± 2 Month,

Year 5 ± 2 Month.

Table 4.2—1 Summary of Study Schedules and Evaluations				
	**Year 2** ± 2 Month	**Year 3** ± 2 Month	**Year 4** ± 2 Month	**Year 5** ± 2 Month
**Physical Exam**	X	X	X	X
**CRST**	X	X	X	X
**QOL (QUEST)**	X	X	X	X
**ET medications**	X	X	X	X
**Adverse Events**	X	X	X	X
**Exit Form**	X	X	X	X

5 DATA ANALYSIS PLAN

5.1 Safety

Only ET-disease, device and procedure-related adverse events will be recorded. Recorded adverse events will be assessed and categorized according to severity, relationship to ET disease, ExAblate procedure, or device. Standard Code of Federal Regulation definitions for Serious Adverse Events (SAEs) and Unanticipated Adverse Device Effects (UADEs) will be used in assessment of AEs.

5.2 Efficacy

Primary effectiveness will be evaluated using the CRST scored by the site assessor based upon patients where unilateral ExAblate thalamotomy was performed. Secondary efficacy will be collected using the QUEST.

5.3 Subject Health Status

The results from the physical exams will be recorded in the CRFs and will be presented.

5.4 Statistical Considerations and Sample Size

There is no statistical consideration or sample size for this study. All ExAblate treated subjects who participated in either ET002 or ET002-CA through Month 12 will be included in this study; ET002-CA was FDA approved under IDE Supplement G120246/S07.

5.5 Missing Data

Analyses will be performed on both observed and data with missing values imputed per the method of last observation carried forward (LOCF) where data for missing visits is assigned the value of the previous visit.

NOTE:

All Demographic, Screening, Baseline, Treatment and Follow-up data through Month 12 are collected under ET002 or ET002-CA and these data will be used in coordination with that collected under this protocol.

5.6 Statistical Analysis Plan.

The analysis will be performed based upon the Statistical Analysis Plan ("SAP") procedures as devised for the pivotal study; FDA Approval of SAP under IDE Supplement G120246/S06.

5.7 Subject Confidentiality

Subject confidentiality will be maintained throughout this study, including all publications. Data collected and entered into the CRFs are the property of the study sponsor. Representatives from the study sponsor or authorized sponsor representatives, the Institutional Review Board, Ethics Committee or other regulatory bodies may receive copies of the study records and may review medical records related to the study.

6 RISK ANALYSIS

Worldwide, over 8000 treatments have been performed to date with the MR guided FUS ExAblate body system. Risk analysis for InSightec ExAblate systems/clinical investigations has been conducted as part of previously approved FDA IDE submissions (G930140, G990151, G990184, G990201, G000203, G010225, G020001, G020182, G050177, and G060023, G070022, G080009, G080206, G100127, G100169, P040003 and subsequent supplements, and P110039). This data has been re-examined by the study sponsor and it has been concluded that this risk analysis has limited applicability to the proposed clinical investigation. The key consideration here is the fact that this proposed study is conducted with an ExAblate transcranial system that is completely different from the body system. This is system is referred to internally as the Brain system. However, in principle, the body and brain systems have the same purpose, namely to coagulate soft tissue within the body by means of MR guided high intensity focused ultrasound.

There are no additional new risks anticipated under this study. All the risks were described under the original treatment protocol and are still active for this protocol. No new treatments are performed here as this is strictly a long-term observational study.

6.1 Criteria for Removal from the Study

The investigator may withdraw subjects from the study as is deemed necessary or deemed to be in the best interests of the subject, such as,

- continued noncompliance with the protocol or study visits,
- severe illness or disability during the study for non-study issues,
- pursuit of subsequent alternative treatment for the same condition, or
- development of intolerable side effects where continued follow-up becomes too burdensome.

In addition, a subject may also chose to exit the study at any time, but will be strongly encouraged to participate in the follow-up visits for safety reasons (continued monitoring of subject safety).

6.2 Data Safety Monitoring Board

A Data Safety Monitoring Board will be used to review all AEs on the study. Their role is to evaluate all AE's that occur throughout the study and determine if they are in fact related to the ExAblate, or some other cause. Investigators will capture all adverse events, and consider the following questions:

- *Was the adverse event serious?*

- *Was the adverse event life-threatening, caused a disability, required or prolonged hospitalization, or caused death?*

- *Was the adverse event device related?*

- *Was the adverse event unexpected?*

- *Is there an unreasonable risk in continuing the trial?*

Adverse Events meeting all the above conditions would require reporting to the FDA, stopping the study pending the results of further investigation, and FDA approval to re-start the study. Following the DSMB review of the event, and if in the opinion of the DSMB, a modification of the study protocol were necessary to provide adequate protection to future study participants, the modification would be implemented prior to reinitiating the investigation. Any such amendment would be reported to the IRB and FDA for their respective approvals to re-start the study as it is required by the applicable regulations.

All adverse events will be assessed for their relationship to the study device or procedure. Standard Code of Federal Regulation (CFR) definitions for SAEs and UADEs will be used in assessment of adverse events.

7 POTENTIAL BENEFITS

There may or may not be any benefit to participating in this study. This technique is still being investigated. It may provide some therapeutic value for subjects with few or no other options due to the great risk that would be involved in open resection. The symptoms may decrease and/or the quality of life of the subject may improve due to relief of symptoms. However, there is no guarantee that this procedure will reduce, eliminate symptoms, or otherwise treat the underlying disorder. Other subjects may benefit from this procedure in the future, if further trials prove it to be a safe and effective therapy.

8 MONITORING PLAN

Clinical Monitoring for this study will be managed by InSightec. The Clinical Monitor is qualified by training and experience to oversee the conduct of this study. The Clinical Monitor's responsibilities include maintaining regular contact with each investigational site through telephone contact and on-site visits, to ensure that:

- The trial is conducted according to FDA and GCP requirements;
- The trial is conducted according to InSightec internal SOPs
- The Investigational Plan is followed;
- Complete, timely, and accurate data are submitted;
- Problems with inconsistent or incomplete data are addressed;
- Complications and unanticipated adverse effects are reported to the Sponsor and the IRB;
- The site facilities will be monitored to stay adequate to meet the requirements of the study.

The Clinical Monitor will initiate the Study either in person or by phone and will continue to perform on-site monitoring visits as frequently as deemed necessary. At this visit and all monitoring visits, the Clinical Monitor will compare the data entered onto the CRFs with the hospital or clinical records (source documents). Source documentation must be available to substantiate proper informed consent procedures, adherence to protocol procedures, adequate reporting and follow-up of AEs, and device procedure information. Findings from the review of CRFs and source documents during a monitoring visit will be discussed with the PI. Completed paper or electronic CRFs will be reviewed prior to data closure at each visit. The dates of the monitoring visits will be recorded in a Log to be kept at the clinical site. During monitoring visits, the Sponsor expects that the study coordinator and the PI will be available, the source documentation will be available, and a suitable environment will be provided for review of Study related documents.

Sites should make every effort to contact all subjects for study follow-up to encourage visit compliance. Sites should keep a log of dates of attempted contact and results. After

3 unsuccessful attempts at contact (e.g., by telephone or email) and sending 1 certified letter to solicit their visit compliance a subject may be considered lost to follow-up.

Monitoring procedures will follow the Sponsor SOPs.

8.1 Electronic Data Capture (EDC)

Electronic CRFs (eCRFs) will be to capture protocol-specific information during the conduct of this study. This electronic data capture of the eCRFs is based on the Oracle Software system, and is designed, run and hosted by Sponsor (Haifa, Israel).

9 INVESTIGATOR RESPONSIBILITIES

The Principal Investigator will be required to sign the Investigator Agreement. All investigators will undergo extensive training on the protocol and operation of the ExAblate system, and provide documentation of their specialized training.

10 REFERENCES

1. Bharucha, N.E., et al., *Prevalence of essential tremor in the Parsi community of Bombay, India.* Arch Neurol, 1988. **45**(8): p. 907-8.
2. Haerer, A.F., D.W. Anderson, and B.S. Schoenberg, *Prevalence of essential tremor. Results from the Copiah County study.* Arch Neurol, 1982. **39**(12): p. 750-1.
3. Hornabrook, R.W. and J.T. Nagurney, *Essential tremor in Papua, New Guinea.* Brain, 1976. **99**(4): p. 659-72.
4. Rajput, A.H., et al., *Essential tremor in Rochester, Minnesota: a 45-year study.* J Neurol Neurosurg Psychiatry, 1984. **47**(5): p. 466-70.
5. Rautakorpi, I., et al., *Essential tremor in a Finnish population.* Acta Neurol Scand, 1982. **66**(1): p. 58-67.
6. Clement, G.T. and K. Hynynen, *A non-invasive method for focusing ultrasound through the human skull.* Physics in Medicine & Biology, 2002. **47**(8): p. 1219-36.
7. Clement, G.T., et al., *A magnetic resonance imaging-compatible, large-scale array for trans-skull ultrasound surgery and therapy.* Journal of Ultrasound in Medicine, 2005. **24**(8): p. 1117-25.
8. Hynynen, K., et al., *Pre-clinical testing of a phased array ultrasound system for MRI-guided noninvasive surgery of the brain--a primate study.* European Journal of Radiology, 2006. **59**(2): p. 149-56.
9. Benabid, A.L., et al., *Long-term electrical inhibition of deep brain targets in movement disorders.* Mov Disord, 1998. **13 Suppl 3**: p. 119-25.
10. Hynynen, K. and F.A. Jolesz, *Demonstration of potential noninvasive ultrasound brain therapy through an intact skull.* Ultrasound Med Biol, 1998. **24**(2): p. 275-83.
11. Cline, H.E., et al., *Magnetic resonance-guided thermal surgery.* Magn Reson Med, 1993. **30**(1): p. 98-106.
12. McDannold, N., et al., *MRI investigation of the threshold for thermally induced blood-brain barrier disruption and brain tissue damage in the rabbit brain.* Magn Reson Med, 2004. **51**(5): p. 913-23.
