# Supplementary material for: Unilateral magnetic resonance-guided focused ultrasound for medication-refractory essential tremor: 5-year continued access study
Source: Front Neurol. 2025 Oct 22;16:1659203. doi: 10.3389/fneur.2025.1659203 (PMC12587677; doi:10.3389/fneur.2025.1659203)
Supplement: Supplementary file 5 [file Data_Sheet_4.pdf]

Statistical Analysis Plan

InSightec

A Pivotal Study to Evaluate the Efficacy and Safety of ExAblate Transcranial MRgFUS Thalamotomy Treatment of Medication Refractory Essential Tremor Subjects

Final

February 10, 2014

Signature Page

**Study Name**	**A Pivotal Study to Evaluate the Efficacy and Safety of ExAblate Transcranial MRgFUS Thalamotomy Treatment of Medication Refractory Essential Tremor Subjects**
**Sponsor**	**InSightec, Inc.**
**SAP Date**	**February 10, 2014**

Written by:

Signature: [illegible]

Date: ___10 FEB 2014____________

Yulia Gavrilov, Ph. D.
Chief Biostatistician
TechnoStat Ltd
Raanana, Israel

Signature: [illegible]

Date: ____10 FEB 2014____________

Yossi Tal, Ph.D.
Managing Director
TechnoStat Ltd
Raanana, Israel

Sponsor Signature:

The undersigned hereby declare that they have examined the Statistical Analysis Plan document and agree to its form and content.

Signature: _____________________

Date: __________________________

Nadir Alikacem, PhD
VP Global Regulatory Affairs & CRO
InSightec

Version History

Version Number	Date
1.0	June 24, 2013
2.0	October 17, 2013
2.1	October 28, 2013
2.2	November 12, 2013
2.3	January 08, 2014
2.4	January 15, 2014
2.5	January 28, 2014
2.6	February 10, 2014

Table of Contents

1 INTRODUCTION 12
2 STUDY OBJECTIVES 12
3 TREATMENT GROUPS 12
4 STUDY SCHEDULE 13
5 ANALYSIS POPULATIONS 14
5.1 SAFETY ANALYSIS POPULATION 14
5.2 EFFICACY ANALYSIS POPULATIONS 14
5.2.1 INTENT TO TREAT (ITT) 14
5.2.2 PER PROTOCOL (PP) 14
5.3 CROSSOVER ANALYSIS POPULATION 14
6 DEFINITION OF ENDPOINTS 15
6.1 SAFETY ENDPOINTS 15
6.2 EFFICACY ENDPOINTS 15
6.2.1 CONFIRMATORY PRIMARY ENDPOINT 15
6.2.2 CONFIRMATORY SECONDARY ENDPOINTS 17
6.2.3 ADDITIONAL SECONDARY ENDPOINTS 19
7 HANDLING OF MISSING DATA 20
7.1 IMPUTATION OF CONFIRMATORY PRIMARY EFFICACY DATA 20
7.2 IMPUTATION OF CONFIRMATORY SECONDARY EFFICACY DATA 21
7.2.1 IMPUTATION OF CONFIRMATORY SECONDARY ENDPOINT 1 (SE1) 21
7.2.2 IMPUTATION OF CONFIRMATORY SECONDARY ENDPOINT 2 (SE2) 21
7.2.3 IMPUTATION OF CONFIRMATORY SECONDARY ENDPOINT 3 (SE3) 22
8 DATA DERIVATION AND TRANSFORMATION 22
9 INTERIM ANALYSIS 23
10 STATISTICAL ANALYSIS 23
10.1 SUBJECT DISPOSITION 23
10.2 BASELINE CHARACTERISTICS 24
10.3 TREATMENT PROCEDURE 26
10.4 BLINDING ASSESSMENT 27
10.5 SAFETY 27
10.6 EFFICACY 28
10.6.1 ADJUSTMENT FOR MULTIPLE COMPARISONS 28
10.6.2 CONFIRMATORY PRIMARY EFFICACY 29
10.6.3 CONFIRMATORY SECONDARY EFFICACY 29
10.6.4 ADDITIONAL SECONDARY EFFICACY 30
10.6.5 COVARIATE ANALYSES 31
10.6.6 SENSITIVITY ANALYSES 33

10.7 CROSSOVER STAGE ANALYSIS 34
11 DATA LISTINGS 35
12 APPENDIX 36
12.1 SUBJECT DISPOSITION 36
12.2 BASELINE CHARACTERISTICS 44
12.3 TREATMENT PROCEDURE 52
12.4 BLINDING ASSESSMENT 57
12.5 SAFETY 59
12.6 EFFICACY 64

List of Figures

FIGURE 1 SUBJECT DISPOSITION FLOW CHART 36
FIGURE 2 COVARIATE ANALYSES: SCATTER PLOT OF SE2 VERSUS AGE (ITT) 97
FIGURE 3 COVARIATE ANALYSES: SCATTER PLOT OF SE2 VERSUS BASELINE CRST SCORE (ITT) 97

List of Tables

TABLE 1 FREQUENCY DISTRIBUTION OF ANSWERS TO INCLUSION CRITERIA 37
TABLE 2 FREQUENCY DISTRIBUTION OF ANSWERS TO EXCLUSION CRITERIA 37
TABLE 3 FREQUENCY DISTRIBUTION OF OVERALL ELIGIBILITY 39
TABLE 4 LISTING OF SCREEN FAILURES 39
TABLE 5 SUBJECT ACCOUNTABILITY BY VISIT AND TREATMENT GROUP 39
TABLE 6 BY-SUBJECT LISTING OF PROTOCOL DEVIATIONS 40
TABLE 7 NUMBER AND PERCENTAGE OF SUBJECTS IN EACH OF THE ANALYSIS POPULATIONS BY TREATMENT GROUP (SAFETY) 40
TABLE 8 LISTING OF SUBJECTS EXCLUDED FROM EACH OF THE ANALYSIS POPULATIONS (SAFETY) 40
TABLE 9 NUMBER AND PERCENTAGE OF SUBJECTS BY CENTER AND TREATMENT GROUP (ITT) 41
TABLE 10 NUMBER AND PERCENTAGE OF SUBJECTS WHO COMPLETED / PREMATURELY DISCONTINUED THE FIRST 3 MONTHS FU OF THE STUDY MAIN STAGE BY TREATMENT GROUP (ITT) 41
TABLE 11 NUMBER AND PERCENTAGE OF SUBJECTS WHO COMPLETED / PREMATURELY DISCONTINUED THE FOLLOW UP PERIOD OF THE STUDY MAIN STAGE FOR EXABLATE GROUP (ITT) 41
TABLE 12 LISTING OF ALL DROPOUTS (ITT) 42
TABLE 13 CROSSOVER STAGE: NUMBER AND PERCENTAGE OF SUBJECTS BY CENTER (CROSSOVER) 42
TABLE 14 CROSSOVER STAGE: NUMBER AND PERCENTAGE OF SUBJECTS WHO COMPLETED / PREMATURELY DISCONTINUED THE STUDY CROSSOVER STAGE (CROSSOVER) 42

TABLE 15 CROSSOVER STAGE: LISTING OF ALL DROPOUTS (CROSSOVER) 42

TABLE 16 CROSSOVER STAGE: SUBJECT ACCOUNTABILITY BY VISIT 43

TABLE 17 DEMOGRAPHIC CHARACTERISTICS: DESCRIPTIVE STATISTICS OF AGE, BMI, HEIGHT AND WEIGHT BY TREATMENT GROUP (SAFETY) 44

TABLE 18 DEMOGRAPHIC CHARACTERISTICS: FREQUENCY DISTRIBUTION OF GENDER AND RACE BY TREATMENT GROUP (SAFETY)................................ 45

TABLE 19 MEDICAL HISTORY: FREQUENCY DISTRIBUTION OF SIGNIFICANT MEDICAL CONDITIONS (OTHER THAN THE PRESENT DISEASE) BY TREATMENT GROUP (SAFETY).. 45

TABLE 20 MEDICAL HISTORY: FREQUENCY DISTRIBUTION OF OTHER SIGNIFICANT MEDICAL CONDITIONS (PROTOCOL SPECIFIC) BY TREATMENT GROUP (SAFETY).. 47

TABLE 21 ESSENTIAL TREMORS HISTORY: DESCRIPTIVE STATISTICS OF TIME FROM INITIAL ET SYMPTOMS, TIME FROM INITIAL ET DIAGNOSIS AND TIME FROM FIRST ET MEDICAL THERAPY BY TREATMENT GROUP (SAFETY) 48

TABLE 22 ESSENTIAL TREMORS HISTORY: FREQUENCY DISTRIBUTION OF FAMILY HISTORY OF ET, INDICATION OF ETIOLOGY DUE TO NEUROLEPTIC DRUG EXPOSURE AND IS SUBJECT CONSIDERED MEDICATION REFRACTORY BY TREATMENT GROUP (SAFETY)............... 49

TABLE 23 LISTING OF SUBJECTS HAVING FAMILY HISTORY OF ET (SAFETY) 49

TABLE 24 FREQUENCY DISTRIBUTION OF MRI EXAMINATION RESULTS BY TREATMENT GROUP (SAFETY).. 50

TABLE 25 FREQUENCY DISTRIBUTION OF CT EXAMINATION RESULTS BY TREATMENT GROUP (SAFETY).. 50

TABLE 26 CRST AND QUEST QUESTIONNAIRE PARAMETERS AT BASELINE BY TREATMENT GROUP (SAFETY).. 51

TABLE 27 COMPARISON OF BASELINE CHARACTERISTICS BETWEEN TREATMENT GROUPS (SAFETY)... 51

TABLE 28 FREQUENCY DISTRIBUTION OF PRE-TREATMENT CHARACTERISTICS BY TREATMENT GROUP (SAFETY)................................ 52

TABLE 29 DESCRIPTIVE STATISTICS FOR VITAL SIGNS BY TIME POINT AND TREATMENT GROUP (SAFETY).. 52

TABLE 30 DESCRIPTIVE STATISTICS OF TREATMENT CHARACTERISTICS BY TREATMENT GROUP (SAFETY).. 53

TABLE 31 FREQUENCY DISTRIBUTION OF TREATED SIDE OF THE BODY BY TREATMENT GROUP (SAFETY).. 54

TABLE 32 FREQUENCY DISTRIBUTION OF PROCEDURE INTERRUPTION OCCURRENCE BY TREATMENT GROUP (SAFETY)... 55

TABLE 33 LISTING OF ALL DEVICE MALFUNCTIONS (SAFETY)..................................... 55

TABLE 34 FREQUENCY DISTRIBUTION OF PROCEDURE TERMINATION PRIOR TO COMPLETION BY TREATMENT GROUP (SAFETY) 55

TABLE 35 LISTING OF REASONS FOR PROCEDURE TERMINATION PRIOR TO COMPLETION (SAFETY) 56

TABLE 36 FREQUENCY DISTRIBUTION OF SUBJECTIVE PERCEPTION OF TREATMENT RECEIVED BY VISIT AND TREATMENT GROUP (SAFETY).. 57

TABLE 37 FREQUENCY DISTRIBUTION OF CORE LAB REVIEWER SUBJECTIVE PERCEPTION OF TREATMENT RECEIVED BY VISIT FOR EXABLATE GROUP (SAFETY) ... 58

TABLE 38 FREQUENCY DISTRIBUTION OF CORE LAB REVIEWER SUBJECTIVE PERCEPTION OF TREATMENT RECEIVED DURING CROSSOVER STAGE BY VISIT FOR EXABLATE GROUP (CROSSOVER) ... 58

TABLE 39 DESCRIPTIVE STATISTICS OF NUMBER OF ADVERSE EVENTS PER SUBJECT BY TREATMENT GROUP (SAFETY) ... 59

TABLE 40 FREQUENCY DISTRIBUTION OF EXPERIENCE OF AT LEAST ONE ADVERSE EVENT BY TREATMENT GROUP (SAFETY) ... 59

TABLE 41 ADVERSE EVENTS STARTED WITHIN 30 DAYS POST TREATMENT BY BODY SYSTEM, PREFERRED TERM, RESOLUTION TIME AND TREATMENT GROUP (SAFETY) ... 59

TABLE 42 ADVERSE EVENTS STARTED WITHIN 31-90 DAYS POST TREATMENT BY BODY SYSTEM, PREFERRED TERM, RESOLUTION TIME AND TREATMENT GROUP (SAFETY) ... 60

TABLE 43 ADVERSE EVENTS STARTED MORE THAN 90 DAYS POST TREATMENT BY BODY SYSTEM, PREFERRED TERM, RESOLUTION TIME FOR EXABLATE GROUP (SAFETY) ... 60

TABLE 44 ADVERSE EVENTS BY BODY SYSTEM, PREFERRED TERM, SEVERITY AND TREATMENT GROUP (SAFETY) ... 61

TABLE 45 ADVERSE EVENTS BY BODY SYSTEM, PREFERRED TERM, RELATION TO TREATMENT AND TREATMENT GROUP (SAFETY) ... 62

TABLE 46 LISTING OF SERIOUS ADVERSE EVENTS BY TREATMENT GROUP (SAFETY) ... 63

TABLE 47 CONFIRMATORY PRIMARY EFFICACY (PE): DESCRIPTIVE STATISTICS OF PERCENT IMPROVEMENT FROM BASELINE AT THREE MONTHS POST-TREATMENT IN THE TREATED (CONTRALATERAL) UPPER EXTREMITY CRST SUB SCORE BY TREATMENT GROUP (ITT) ... 64

TABLE 48 CONFIRMATORY SECONDARY EFFICACY (SE1): DESCRIPTIVE STATISTICS OF IMPROVEMENT FROM BASELINE AT THREE MONTHS POST-TREATMENT IN QUALITY OF LIFE IN ESSENTIAL TREMOR (QUEST) OUTCOME BY TREATMENT GROUP (ITT) ... 64

TABLE 49 CONFIRMATORY SECONDARY EFFICACY (SE2): DESCRIPTIVE STATISTICS OF PERCENT IMPROVEMENT FROM BASELINE AT TWELVE MONTHS POST-TREATMENT IN THE TREATED (CONTRALATERAL) UPPER EXTREMITY CRST SUB SCORE IN EXABLATE GROUP (ITT) ... 65

TABLE 50 CONFIRMATORY SECONDARY EFFICACY (SE3): DESCRIPTIVE STATISTICS OF PERCENT IMPROVEMENT FROM BASELINE AT THREE MONTHS POST-TREATMENT IN FUNCTIONAL DISABILITIES TOTAL SCORE, AS MEASURED BY CRST PART-C BY TREATMENT GROUP (ITT) ... 65

TABLE 51 ADDITIONAL SECONDARY EFFICACY (PE): DESCRIPTIVE STATISTICS OF PERCENT IMPROVEMENT FROM BASELINE AT THREE MONTHS POST-TREATMENT IN THE TREATED (CONTRALATERAL) UPPER EXTREMITY CRST SUB SCORE BY TREATMENT GROUP (PP) ... 66

TABLE 52 ADDITIONAL SECONDARY EFFICACY (SE1): DESCRIPTIVE STATISTICS OF IMPROVEMENT FROM BASELINE AT THREE

MONTHS POST-TREATMENT IN QUALITY OF LIFE IN ESSENTIAL TREMOR (QUEST) OUTCOME BY TREATMENT GROUP (PP) 66

TABLE 53 ADDITIONAL SECONDARY EFFICACY (SE2): DESCRIPTIVE STATISTICS OF PERCENT IMPROVEMENT FROM BASELINE AT TWELVE MONTHS POST-TREATMENT IN THE TREATED (CONTRALATERAL) UPPER EXTREMITY CRST SUB SCORE IN EXABLATE GROUP (PP) 67

TABLE 54 ADDITIONAL SECONDARY EFFICACY (SE3): DESCRIPTIVE STATISTICS OF PERCENT IMPROVEMENT FROM BASELINE AT THREE MONTHS POST-TREATMENT IN FUNCTIONAL DISABILITIES TOTAL SCORE, AS MEASURED BY CRST PART-C BY TREATMENT GROUP (PP) 67

TABLE 55 ADDITIONAL SECONDARY EFFICACY: DESCRIPTIVE STATISTICS OF PART A AND B CRST OVER TIME BY SIDE OF THE BODY AND TREATMENT GROUP (ITT) 68

TABLE 56 ADDITIONAL SECONDARY EFFICACY: DESCRIPTIVE STATISTICS OF PART A AND B CRST OVER TIME BY SIDE OF THE BODY AND TREATMENT GROUP (PP) 68

TABLE 57 ADDITIONAL SECONDARY EFFICACY: DESCRIPTIVE STATISTICS OF PART B SCORE FOR TREATED SIDE OF THE BODY FOR HANDWRITING OVER TIME BY TREATMENT GROUP AND ANALYSIS POPULATION (ITT, PP) 69

TABLE 58 ADDITIONAL SECONDARY EFFICACY: DESCRIPTIVE STATISTICS OF PART B SCORE FOR TREATED SIDE OF THE BODY FOR DRAWING A OVER TIME BY TREATMENT GROUP AND ANALYSIS POPULATION (ITT, PP) 70

TABLE 59 ADDITIONAL SECONDARY EFFICACY: DESCRIPTIVE STATISTICS OF PART B SCORE FOR TREATED SIDE OF THE BODY FOR DRAWING B OVER TIME BY TREATMENT GROUP AND ANALYSIS POPULATION (ITT, PP) 70

TABLE 60 ADDITIONAL SECONDARY EFFICACY: DESCRIPTIVE STATISTICS OF PART B SCORE FOR TREATED SIDE OF THE BODY FOR DRAWING C OVER TIME BY TREATMENT GROUP AND ANALYSIS POPULATION (ITT, PP) 71

TABLE 61 ADDITIONAL SECONDARY EFFICACY: DESCRIPTIVE STATISTICS OF PART B SCORE FOR TREATED SIDE OF THE BODY FOR POURING OVER TIME BY TREATMENT GROUP AND ANALYSIS POPULATION (ITT, PP) 72

TABLE 62 ADDITIONAL SECONDARY EFFICACY: DESCRIPTIVE STATISTICS OF PART C SCORE FOR SPEAKING OVER TIME BY TREATMENT GROUP AND ANALYSIS POPULATION (ITT, PP) 72

TABLE 63 ADDITIONAL SECONDARY EFFICACY: DESCRIPTIVE STATISTICS OF PART C SCORE FOR EATING OVER TIME BY TREATMENT GROUP AND ANALYSIS POPULATION (ITT, PP) 73

TABLE 64 ADDITIONAL SECONDARY EFFICACY: DESCRIPTIVE STATISTICS OF PART C SCORE FOR DRINKING OVER TIME BY TREATMENT GROUP AND ANALYSIS POPULATION (ITT, PP) 74

TABLE 65 ADDITIONAL SECONDARY EFFICACY: DESCRIPTIVE STATISTICS OF PART C SCORE FOR HYGIENE OVER TIME BY TREATMENT GROUP AND ANALYSIS POPULATION (ITT, PP) 74

TABLE 66 ADDITIONAL SECONDARY EFFICACY: DESCRIPTIVE STATISTICS OF PART C SCORE FOR DRESSING OVER TIME BY TREATMENT GROUP AND ANALYSIS POPULATION (ITT, PP).. 75

TABLE 67 ADDITIONAL SECONDARY EFFICACY: DESCRIPTIVE STATISTICS OF PART C SCORE FOR WRITING OVER TIME BY TREATMENT GROUP AND ANALYSIS POPULATION (ITT, PP).. 76

TABLE 68 ADDITIONAL SECONDARY EFFICACY: DESCRIPTIVE STATISTICS OF PART C SCORE FOR WORKING OVER TIME BY TREATMENT GROUP AND ANALYSIS POPULATION (ITT, PP).. 76

TABLE 69 ADDITIONAL SECONDARY EFFICACY: DESCRIPTIVE STATISTICS OF PART C SCORE FOR SOCIAL ACTIVITIES OVER TIME BY TREATMENT GROUP AND ANALYSIS POPULATION (ITT, PP)...................... 77

TABLE 70 ADDITIONAL SECONDARY EFFICACY: DESCRIPTIVE STATISTICS OF PART C OVERALL SCORE OVER TIME BY TREATMENT GROUP AND ANALYSIS POPULATION (ITT, PP) 78

TABLE 71 ADDITIONAL SECONDARY EFFICACY: DESCRIPTIVE STATISTICS OF OVERALL CRST SCORE OVER TIME BY TREATMENT GROUP AND ANALYSIS POPULATION (ITT, PP) 78

TABLE 72 ADDITIONAL SECONDARY EFFICACY: DESCRIPTIVE STATISTICS OF OVERALL QUALITY OF LIFE SCORE OVER TIME BY TREATMENT GROUP AND ANALYSIS POPULATION (ITT, PP) 79

TABLE 73 ADDITIONAL SECONDARY EFFICACY: DESCRIPTIVE STATISTICS OF WAKING HOURS WITH TREMOR IN ANY BODY PART OVER TIME BY TREATMENT GROUP AND ANALYSIS POPULATION (ITT, PP)...................... 80

TABLE 74 ADDITIONAL SECONDARY EFFICACY: DESCRIPTIVE STATISTICS OF TREMOR SEVERITY SCORE FOR HEAD OVER TIME BY TREATMENT GROUP AND ANALYSIS POPULATION (ITT, PP) 80

TABLE 75 ADDITIONAL SECONDARY EFFICACY: DESCRIPTIVE STATISTICS OF TREMOR SEVERITY SCORE FOR VOICE OVER TIME BY TREATMENT GROUP AND ANALYSIS POPULATION (ITT, PP) 81

TABLE 76 ADDITIONAL SECONDARY EFFICACY: DESCRIPTIVE STATISTICS OF TREMOR SEVERITY SCORE FOR TREATED ARM/HAND OVER TIME BY TREATMENT GROUP AND ANALYSIS POPULATION (ITT, PP) 82

TABLE 77 ADDITIONAL SECONDARY EFFICACY: DESCRIPTIVE STATISTICS OF TREMOR SEVERITY SCORE FOR NON-TREATED ARM/HAND OVER TIME BY TREATMENT GROUP AND ANALYSIS POPULATION (ITT, PP).... 82

TABLE 78 ADDITIONAL SECONDARY EFFICACY: DESCRIPTIVE STATISTICS OF TREMOR SEVERITY SCORE FOR LEG/FOOT CONTRALATERAL TO BRAIN TREATMENT OVER TIME BY TREATMENT GROUP AND ANALYSIS POPULATION (ITT, PP) 83

TABLE 79 ADDITIONAL SECONDARY EFFICACY: DESCRIPTIVE STATISTICS OF TREMOR SEVERITY SCORE FOR LEG/FOOT IPSILATERAL TO BRAIN TREATMENT OVER TIME BY TREATMENT GROUP AND ANALYSIS POPULATION (ITT, PP) 84

TABLE 80 ADDITIONAL SECONDARY EFFICACY: DESCRIPTIVE STATISTICS OF QUEST COMMUNICATION SCORE OVER TIME BY TREATMENT GROUP AND ANALYSIS POPULATION (ITT, PP) 84

TABLE 81 ADDITIONAL SECONDARY EFFICACY: DESCRIPTIVE STATISTICS OF QUEST WORK AND FINANCES SCORE OVER TIME BY TREATMENT GROUP AND ANALYSIS POPULATION (ITT, PP) 85

TABLE 82 ADDITIONAL SECONDARY EFFICACY: DESCRIPTIVE STATISTICS OF QUEST HOBBIES AND LEISURE SCORE OVER TIME BY TREATMENT GROUP AND ANALYSIS POPULATION (ITT, PP) 86

TABLE 83 ADDITIONAL SECONDARY EFFICACY: DESCRIPTIVE STATISTICS OF QUEST PHYSICAL SCORE OVER TIME BY TREATMENT GROUP AND ANALYSIS POPULATION (ITT, PP) 86

TABLE 84 ADDITIONAL SECONDARY EFFICACY: DESCRIPTIVE STATISTICS OF QUEST PSYCHOSOCIAL SCORE OVER TIME BY TREATMENT GROUP AND ANALYSIS POPULATION (ITT, PP) 87

TABLE 85 ADDITIONAL SECONDARY EFFICACY: DESCRIPTIVE STATISTICS OF QUEST SUMMARY OF DIMENSIONS TOTAL SCORE OVER TIME BY TREATMENT GROUP AND ANALYSIS POPULATION (ITT, PP) 88

TABLE 86 COVARIATE ANALYSES: DESCRIPTIVE STATISTICS OF PRIMARY ENDPOINT (PE) BY COVARIATE AND TREATMENT GROUP (ITT) 89

TABLE 87 COVARIATE ANALYSES: DESCRIPTIVE STATISTICS OF FIRST SECONDARY ENDPOINT (SE1) BY COVARIATE AND TREATMENT GROUP (ITT) 90

TABLE 88 COVARIATE ANALYSES: DESCRIPTIVE STATISTICS OF THIRD SECONDARY ENDPOINT (SE3) BY COVARIATE AND TREATMENT GROUP (ITT) 92

TABLE 89 COVARIATE ANALYSES: P-VALUES FOR MAIN EFFECT OF TREATMENT, COVARIATE AND INTERACTION EFFECTS WHEN PREDICTING PE, SE1 AND SE3 (ITT) 93

TABLE 90 COVARIATE ANALYSES: DESCRIPTIVE STATISTICS OF SECOND SECONDARY ENDPOINT (SE2) BY COVARIATE IN EXABLATE GROUP ALONG WITH COMPARISON P-VALUE (ITT) 95

TABLE 91 SENSITIVITY ANALYSES: WORST CASE – DESCRIPTIVE STATISTICS OF PERCENT IMPROVEMENT FROM BASELINE AT THREE MONTHS POST-TREATMENT IN THE TREATED (CONTRALATERAL) UPPER EXTREMITY CRST SUB SCORE BY TREATMENT GROUP (ITT) 98

TABLE 92 SENSITIVITY ANALYSES: BEST CASE – DESCRIPTIVE STATISTICS OF PERCENT IMPROVEMENT FROM BASELINE AT THREE MONTHS POST-TREATMENT IN THE TREATED (CONTRALATERAL) UPPER EXTREMITY CRST SUB SCORE BY TREATMENT GROUP (ITT) 98

TABLE 93 SENSITIVITY ANALYSES: MULTIPLE IMPUTATIONS – DESCRIPTIVE STATISTICS OF PERCENT IMPROVEMENT FROM BASELINE AT THREE MONTHS POST-TREATMENT IN THE TREATED (CONTRALATERAL) UPPER EXTREMITY CRST SUB SCORE BY TREATMENT GROUP FOR EACH OF THE 10 IMPUTED DATA SETS (ITT) 98

TABLE 94 SENSITIVITY ANALYSES: MULTIPLE IMPUTATIONS – OVERALL RESULT (10 IMPUTED DATASETS COMBINED) (ITT) 100

List of Abbreviations

AE	Adverse Events
BMI	Body Mass Index
CRF	Case Report Form
CRST	Clinical Rating Scale for Tremor
CT	Computer Tomography
DVT	Deep Vein Thrombosis
ET	Essential Tremor
ITT	Intent to Treat
LOCF	Last Observation Carried Forward
MRI	Magnetic Resonance Imaging
PHQ	Patient Health Questionnaire
PP	Per Protocol
QUEST	Quality of Life in Essential Tremor
SAE	Serious Adverse Events
SAP	Statistical Analysis Plan
SF	Screen Failure

1 Introduction

The current document describes a full statistical analysis plan (SAP) for the ET-002 Study. ET-002 is a prospective, multi-center, randomized, double-blind (to subjects and Tremor Core Lab assessor), two-arm study. The design of this study includes two stages: Main and Crossover. In the Main stage subjects are randomized in a 3:1 ratio to either ExAblate or Sham Control (henceforth "ExAblate" and "Sham," respectively). The Crossover stage of the study is designed for subjects randomized to the Sham group in the Main stage to receive an unblinded ExAblate treatment, if eligible, at 3-months follow-up post Sham treatment, and to be followed in a similar fashion as the Main stage of ExAblate subjects in terms of planned follow-up visits and assessments.

2 Study Objectives

The objectives of this trial are to evaluate the efficacy and safety of ExAblate system in treatment of medication-refractory tremor in subjects with essential tremor.

The Main stage of the study relates to subjects randomized to either ExAblate or Sham, while the Crossover stage of the study examines outcomes for subjects randomized to Sham who eventually received active treatment with ExAblate. Both Main and Crossover stages of the study provide information on the safety and efficacy of the procedure up to twelve months post-treatment. However, only the Main stage will be used to test device safety and efficacy for the purpose of regulatory approval. The Crossover stage will serve to follow-up safety and efficacy to 12 months.

3 Treatment Groups

Each subject in the Main stage of the study will be randomly assigned to either of the following treatment groups:

- ExAblate – Active treatment with ExAblate
- Sham – Sham Control treatment

The randomization scheme will be 3:1 ExAblate-to-Sham.

Subjects in the Crossover stage of the study will undergo active treatment with ExAblate following their initial Sham treatment.

4 Study Schedule

The following table summarizes the sequence of procedures that a subject in this study will follow.

Summary of Study Schedules and Measurements									
	**Screening**	**Baseline Assessment**	**Treatment**	**1 Day**	**1 Week**	**1 Month**	**3 Month**	**6 Month**	**12 Month**
**Consent**	X								
**Eligibility Evaluation with Labs**	X	X							
**Medications**	X	X	X	X	X	X	X	X	X
**30 Day Medication Stabilization**		X							
**Medical History**	X								
**Physical Exam**	X			X	X	X	X	X	X
**Neurological Status**	X		X	X	X	X	X	X	X
**CRST (Unblinded Site Assessor)**	X							X	X
**CRST (Blinded Site Assessor)**		X				X	X		
**CRST (Blinded Tremor Core Lab)**		X				X	X	X	X
**QUEST**	X	X				X	X	X	X
**PHQ-9**	X					X	X	X	X
**CT**		X							
**MRI**		X							X
**Treatment**			X						
**Adverse Events**			X	X	X	X	X	X	X
**Exit Form**									X

5 Analysis Populations

5.1 Safety Analysis Population

The Safety analysis population will include all randomized subjects who received at least one sonication – ExAblate or Sham – in the Main stage of the study, as indicated by the following:

- CRF field "Number of Performed Sonications" on the ExAblate Treatment Form has a value of at least one

  or

- At least one CRF field “Sonication Duration [sec]” on Sonication Form has a value greater than zero.

5.2 Efficacy Analysis Populations

5.2.1 Intent to Treat (ITT)

The ITT analysis population will include all Safety subjects for whom there exist valid baseline measurement and at least one post-baseline measurement on the primary efficacy data.

5.2.2 Per Protocol (PP)

The PP analysis population will include all ITT subjects who have observed primary efficacy data at three months and have no major protocol violations likely to affect outcome.

5.3 Crossover Analysis Population

The Crossover analysis population will include all subjects who received at least one sonication in the Crossover stage of the study.

6 Definition of Endpoints

6.1 Safety Endpoints

The safety endpoints are:

- Adverse Events (AE's)
- Serious Adverse Events (SAE's)

6.2 Efficacy Endpoints

6.2.1 Confirmatory Primary Endpoint

The confirmatory primary efficacy endpoint in this study is Percent Improvement from Baseline at three months post-treatment in the treated (contralateral) upper extremity CRST sub score. For each subject the primary endpoint will be calculated as follows:

1. Identify which side of the brain was treated, as indicated by CRF field “Side of Brain being treated” on the ExAblate Treatment Form
2. If the RIGHT side of brain was treated, then the Tremor Core LAB variables from the LEFT side of the CRST assessment (contralateral side) will be used:
   - Part A = item #6 (LUE tremor): Rest + Posture + Action/Intention

     Note:

     - ➔ If only two individual rates are obtained, the sum will be calculated over the two available rates.
     - ➔ If a single individual rate is obtained, Part A will be considered as missing and should be imputed as described in Section 7.1.
   - Part B = item #11 Left* + item #12 Left + item #13 Left + item #14 Left + item #15 Left

     *Item #11 will be taken into account only for left-handed subjects. For right-handed subjects Part B will be the sum of 4 items only, without item #11.

     Note:

     - ➔ If a single item is missing, the sum will be calculated over the available items – four items for left-handed subjects and three items for right-handed subjects.

➔ If more than one item is missing, Part B will be considered as missing and should be imputed as described in Section 7.1.

3. If the LEFT side of brain was treated, then the Tremor Core LAB variables from the RIGHT side of the CRST assessment (contralateral side) will be used:
   - Part A = item #5 (RUE tremor): Rest + Posture + Action/Intention

     Note:

     ➔ If only two individual rates are obtained, the sum will be calculated over the two available rates.

     ➔ If a single individual rate is obtained, Part A will be considered as missing and should be imputed as described in Section 7.1.
   - Part B = item #11 Right* + item #12 Right + item #13 Right + item #14 Right + item #15 Right

     *Item #11 will be taken into account only for right-handed subjects. For left-handed subjects Part B will be the sum of 4 items only, without item #11.

     Note:

     ➔ If a single item is missing, the sum will be calculated over the available items – four items for right-handed subjects and three items for left-handed subjects.

     ➔ If more than one item is missing, Part B will be considered as missing and should be imputed as described in Section 7.1.
4. Denote the treated (contralateral) upper extremity CRST sub score at visit [k] as $\text{CRST}_{[\text{contralateral, k}]}$, then:

$$\text{CRST}_{[\text{contralateral, k}]} = \frac{Part\ A + Part\ B}{Total}$$

Where, *Total* is the maximal sum that could be achieved in Part A and Part B, based on the available items (obtained and imputed). Note that Total should be re-adjusted for each patient based on his/her available information.

For example, if the following conditions for a subject hold

- The treated side of the body is ipsilateral to handedness,
- One item in Part A is missing
- All five items in Part B are obtained

then the Total = 8 (Maximum that could be achieved in Part A) + 20 (Maximum that could be achieved in Part B) = 28

Note: Lower $CRST_{[contralateral,\ k]}$ scores are better than higher scores.

5. The confirmatory primary efficacy endpoint (denoted as PE) – Percent Improvement from Baseline at three months post-treatment in the treated (contralateral) upper extremity CRST sub score – will be calculated as follows:

$$PE = \frac{CRST_{[contralateral,\ Baseline]} - CRST_{[contralateral,\ 3\ months\ FU]}}{CRST_{[contralateral,\ Baseline]}} \times 100$$

Note: higher PE values represent improvement.

For both stages (Main and Crossover) the primary efficacy endpoint will be calculated using Baseline score, measured before Main stage treatment initiation.

6.2.2 Confirmatory Secondary Endpoints

The confirmatory secondary efficacy endpoints in this study are the following:

- SE1=Improvement from Baseline at three months in Quality of Life in Essential Tremor (QUEST). SE1 will be calculated as follows:
  - Categorize the 30 questions into 5 dimensions as follows:
    - Communication: #1 – #3
    - Work and Finances: #4 – #9
    - Hobbies and Leisure: #10 – #12
    - Physical: #13 – #21
    - Psychosocial: #22 – #30
  - Score all applicable questionnaire answers as follows:
    - Never = 0
    - Rarely = 1
    - Sometimes = 2
    - Frequently = 3
    - Always = 4

  Note:

  ✓ All questions answered "N/A" will be excluded from the calculation (only questions 4, 5, 7, 8 and 14 can be answered "N/A")

- ✓ Questions 6, 7, 11 and 12 can only be scored at two values – either a 0 or a 4

- For each subject, calculate dimension score as a percentage of total possible score for each dimension separately. Denote dimension score for dimension [i] at visit [k] as $DS_{[i, k]}$, then:

$$DS_{[i,k]} = \frac{Total\ \boldsymbol{applicable}\ points_{[i,k]}}{Total\ possible\ points\ (\#\ of\ \boldsymbol{applicable}\ questions\ \times 4)_{[i,k]}} \times 100$$

Note: missing data should be imputed as described in Section 7.2.1.

- For each subject, calculate QUEST summary of dimensions total score as a mean of the five dimension scores. Denote QUEST summary of dimensions total score at visit [k] as $QUEST_{[k]}$, then:

$$QUEST_{[k]} = \frac{\sum_{i=1}^{5} DS_{[i,k]}}{5}$$

Note: lower $QUEST_{[k]}$ scores are better than higher scores.

- For each subject, calculate SE1 as follows:

$$SE1 = \frac{QUEST_{[Baseline]} - QUEST_{[3\ months\ FU]}}{QUEST_{[Baseline]}}$$

Note: higher SE1 values represent improvement.

- SE2=Percent Improvement from Baseline at twelve months post-treatment in the treated (contralateral) upper extremity CRST sub score. SE2 will be calculated similarly to PE from the previous section, based on 12 months FU data.

- SE3=Percent Improvement from Baseline at three months post-treatment in functional disabilities CRST total score, as measured by CRST Part C. Note, that Part C is performed by Site Assessor only. SE3 will be calculated for each subject as follows:
  - Denote functional disabilities CRST Part C total score at visit [k] as $CRST\_C_{[k]}$, then:

$CRST\_C_{[k]}$ = average of answers to items #16-#23 at visit [k]

Note: if no more than two items are missing, the average will be calculated over the available items; otherwise, CRST Part C total score will be considered missing and should be imputed as described in Section 7.2.3.

- Calculate SE3 as follows:

$$SE3 = \frac{CRST\_C_{[Baseline]} - CRST\_C_{[3\ months\ FU]}}{CRST\_C_{[Baseline]}} \times 100$$

Note: higher SE3 values represent improvement.

6.2.3 Additional Secondary Endpoints

The additional secondary endpoints in this trial include, for each subject:

- Clinical Rating Scale for Tremor (CRST) Parameters:
  - Part A and B CRST Score, as assessed by Core LAB Reviewer:
    - For treated side of the body = $CRST_{[contralateral,\ k]}$, as defined in Section 6.2.1
    - For non-treated side of the body = $CRST_{[ipsilateral,\ k]}$, which is defined similar to $CRST_{[contralateral,\ k]}$ when using values obtained from upper extremity ipsilateral to the side of brain being treated
  - Part B Score for treated side of the body, as assessed by Core LAB Reviewer, for each motor task
  - Part C Score, as assessed by Site Assessor:
    - For each item separately
    - Overall, defined as a sum of all individual items
  - Overall CRST Score, defined as "Total A + Total B + Total C", where:

    Total A = sum of all severity scores of Part A items (1-10 at rest, posture and action/intention), as assessed by Core Lab Reviewer, excluding Head Tremor at Rest

    Total B = sum of all severity scores of Part B items (11-15, right and left), as assessed by Core Lab Reviewer

    Total C = sum of all severity scores of Part C items (16-23), as assessed by Site Assessor

- Quality of Life in Essential Tremor Parameters, as assessed by QUEST questionnaire:
  - Overall Quality of Life Score, as defined by CRF field “Overall, how would you rate your quality of life?”
  - Waking Hours with Tremor in Any Body Part, as defined by CRF field “On a typical day, how many of your waking hours do you have tremor in ANY body part?”
  - Tremor Severity Score, separately for the following categories:
    - Head
    - Voice
    - Treated arm/hand
    - Non-treated arm/hand
    - Treated leg/foot
    - Non-treated leg/foot
  - QUEST Parameters (based on questions 1-30), as defined in Section 6.2.2:
    - Dimension Score ($DS_{[i, k]}$), separately for each dimension
    - Summary of Dimensions Total Score ($QUEST_{[k]}$)

7 Handling of Missing Data

ITT analysis population: missing confirmatory primary and secondary efficacy data will be imputed using the last observation carried forward (LOCF) method, as described in Sections 7.1 and 7.2 below. Other missing data will not be imputed; only observed data will be used.

Safety, PP and Crossover analysis populations: all missing data will not be imputed; only observed data will be used.

7.1 Imputation of Confirmatory Primary Efficacy Data

Confirmatory primary efficacy endpoint (PE) is considered missing if either Part A or Part B at 3 Months FU is missing.

<u>Imputation of Part A</u>

Part A is considered missing if at least two out of the three individual rates within item #5 (or #6) are missing. Missing rates at 3 Months FU will be imputed using LOCF method, i.e. rates from 1 Month FU will be carried forward. If a single individual rate is obtained and two other rates are missing, the non-missing rate will be taken as is and will not be imputed.

<u>Imputation of Part B</u>

Part B is considered missing if more than one required items is missing. Missing items at 3 Months FU will be imputed using LOCF method, i.e. items from 1 Month FU will be carried forward. The obtained items will be taken as is and will not be imputed.

7.2 Imputation of Confirmatory Secondary Efficacy Data

7.2.1 Imputation of Confirmatory Secondary Endpoint 1 (SE1)

SE1 is defined as average over five QUEST dimension scores. Thus, SE1 is considered missing if at least one dimension score is missing. Below we define when and how each of these dimension scores should be imputed.

<u>Communication, Work and Finances, Hobbies and Leisure Dimensions</u>

The dimension score is considered missing if at least one of applicable items (within a specific dimension) is missing. Missing applicable items will be imputed using LOCF method.

<u>Physical and Psychosocial Dimensions</u>

The dimension score is considered missing if more than two applicable items (within a specific dimension) are missing. Missing applicable items will be imputed using LOCF method.

7.2.2 Imputation of Confirmatory Secondary Endpoint 2 (SE2)

The imputation of SE2 will be done similarly to PE, as described in Section 7.1.

7.2.3 Imputation of Confirmatory Secondary Endpoint 3 (SE3)

CRST Part C total score is considered missing if more than two items are missing. Missing items at 3 Months FU will be imputed using LOCF method (where applicable). The obtained (non-missing) items will be taken as is and will not be imputed.

8 Data Derivation and Transformation

Data not originally part of the CRF will be derived as follows:

- Age [years] = (Date of Informed Consent – Date of Birth + 1) / 365.25
- BMI [kg/m^2] = Weight (kg) / Height $(m)^2$
- Time from Initial ET Symptoms [years] = (Date of Informed Consent – Approximate Date of Initial ET Symptoms[1] + 1) / 365.25
- Time from Initial ET Diagnosis [years] = (Date of Informed Consent – Date of Initial ET Diagnosis[1] + 1) / 365.25
- Time from First ET Medical Therapy [years] = (Date of Informed Consent – Date of First ET Medical Therapy[1] + 1) / 365.25
- Handedness (Right / Left) is indicated by CRF field “Right or Left Handed” on Baseline CRST Form, as assessed by Site Assessor
- Treated Side of the Body (Right / Left) is defined as the opposite side of the brain being treated, indicated by CRF field “Side of Brain being treated” on the ExAblate Treatment Form
- Time Inside Scanner [min] = Time Out of Scanner – Time In Scanner
- Sonication Treatment Time [min] = Sonication End Time – Sonication Start Time
- Target Size [cm^3] = $\frac{4}{3}\pi(\frac{1}{2} \times SI[cm] \times RL[cm] \times AP[cm])$, where SI, RL and AP are the target dimensions Superoinferior, Right-Left and Anteroposterior respectively, as collected on the ExAblate Treatment Form
- The following will be provided by InSightec:

[1] If only Day is missing then the 15th of the Month will be imputed; if both Day and Month are missing then the 1st of July will be imputed.

- By-subject listing of all protocol deviations
- Coding of all Adverse Events

9 Interim Analysis

No interim analysis is planned for the ET-002 study.

10 Statistical Analysis

All statistical analyses will be carried out using SAS® Version 9.2 or higher under Windows® Server 2008 Terminal.

The data will be summarized in tables listing the mean, standard deviation, minimum, median, maximum and number of subjects for continuous data, or in tables listing count and percentage for categorical data where appropriate.

10.1 Subject Disposition

The following will be provided based on all recruited subjects:

- Subject disposition flow chart (Figure 1)
- Frequency distribution of answers (Yes / No) to inclusion / exclusion criteria (Table 1, Table 2)
- Frequency distribution of overall eligibility for the study (Yes / No) based on inclusion / exclusion answers (Table 3)
- Listing of screen failures along with the reason for not entering the study (Table 4)
- Subject accountability by visit and treatment group (Table 5)
- By-subject listing of all protocol deviations (Table 6)

The following will be provided using Safety analysis population:

- Number and percentage of subjects in each of the analysis populations by treatment group (Table 7)

- Listing of subjects excluded from each of the analysis populations along with the reason for exclusion (Table 8)

The following will be provided using ITT analysis population:

- Number and percentage of subjects by center and treatment group (Table 9)
- Termination of the Study Main Stage:
  - Number and percentage of subjects who completed / prematurely discontinued the first 3 months FU of the study Main stage by treatment group (Table 10)
  - Number and percentage of subjects who completed / prematurely discontinued the follow up period (from 3 up to 12 months) of the study Main stage for ExAblate group only (Table 11)
  - Listing of all dropouts along with reason for termination, treatment group and last available visit (Table 12)

The following will be provided using Crossover analysis population:

- Number and percentage of subjects by center (Table 13)
- Termination of the Study Crossover Stage:
  - Number and percentage of subjects who completed / prematurely discontinued the study crossover stage (Table 14)
  - Listing of all dropouts along with reason for termination and last available visit (Table 15)
- Subject accountability by visit (Table 16)

10.2 Baseline Characteristics

Baseline characteristics will be analyzed using the Safety analysis population. Descriptive statistics or frequency distribution, as appropriate, by treatment group will be provided for the following:

- Demographic Characteristics and Vital Signs:
  - Age, BMI, Height, Weight (Table 17)
  - Gender, Race (Table 18)
- Medical History:

- Significant medical conditions (other than the present disease) (Table 19)
- Other significant medical conditions (protocol specific) (Table 20)

- Essential Tremors History:
  - Time from Initial ET Symptoms (Table 21)
  - Time from Initial ET Diagnosis (Table 21)
  - Time from First ET Medical Therapy (Table 21)
  - Family History of ET (Table 22)
    - Listing of subjects having family history of ET along with specification of relationship degree and the number of relatives (Table 23)
  - Indication of etiology due to neuroleptic drug exposure (Table 22)
  - Is subject considered medication refractory? (Table 22)
- MRI Examination (Table 24):
  - Any arteriovenous malformations that require treatment
  - Any aneurysms that require treatment
  - Evidence of a recent hemorrhage
- CT Examination (Table 25):
  - Any calcifications present within the treated area
  - Any implants within the skull or brain
  - Is the skull suitable for treatment?
  - History of DVT or systemic thrombosis
    - Evidence of acute thrombosis in lower extremities for subjects who had a history of DVT or systemic thrombosis
- CRST and QUEST questionnaire parameters at Baseline, as defined in Sections 6.2.1 and 6.2.2 (Table 26):
  - The Treated (Contralateral) Upper Extremity CRST Sub Score ($CRST_{[contralateral, Baseline]}$)
  - QUEST Summary of Dimensions Total Score ($QUEST_{[Baseline]}$)
  - Functional Disabilities CRST Part C Total Score ($CRST\_C_{[Baseline]}$)

Where applicable, baseline characteristics will be compared between treatment groups using two-sided t-test for continuous normal variables, Wilcoxon rank-sum test for

continuous variables with distribution deviating from normal and Fisher's Exact test for categorical variables (Table 27).

10.3 Treatment Procedure

Treatment procedure characteristics will be analyzed using the Safety analysis population.

Descriptive statistics or frequency distribution, as appropriate, by treatment group will be provided for the following:

- Pre-Treatment Characteristics (Table 28):
  - Head Condition
  - Scars or Lesions on Head
  - Was the subject "off" medication for at least 12 hours prior to the treatment?
- Vital Signs per Time Point (Table 29):
  - Systolic Blood Pressure
  - Diastolic Blood Pressure
  - Heart Rate
  - O_2 Saturation
- Treatment Characteristics (Table 30):
  - Time Inside Scanner
  - Sonication Treatment Time
  - Target Size
  - Minimal Energy
  - Maximal Energy
  - Number of Performed Sonications
  - Treated Side of the Body (Right / Left) (Table 31)
- Procedure Interruption / Early Termination:
  - Interruption:
    - Procedure interrupted for more than 30 minutes due to MR system problems (Table 32)
    - Procedure interrupted for more than 30 minutes due to ExAblate system problems (Table 32)

    - Listing of all device malfunctions (Table 33)
  - Early Termination:
    - Procedure terminated prior to completion (Table 34)
    - Listing of all termination reasons (Table 35)

10.4 Blinding Assessment

Blinding assessment will be analyzed using the Safety and Crossover analysis populations separately. Frequency distribution of subjective perception of treatment received by visit and treatment group will be provided (Table 36, Table 37, Table 38) for the following blinding assessments:

- Subject Perception
- Site Assessor Perception
- Core Lab Reviewer Perception

Where appropriate, Fisher's exact test will be conducted to compare subjective perception between treatment groups.

10.5 Safety

All safety analyses will be performed on the Safety and Crossover analysis populations – separately and combined, and will be descriptive and narrative in nature. Note that mock tables are presented for Safety population only. For Crossover and combined (Safety and Crossover) populations the tables will be prepared in the similar manner, presenting ExAblate group only. The following will be provided by treatment group:

- Adverse Events:
  - Descriptive statistics of Number of Adverse Events per Subject (Table 39)
  - Frequency distribution of Experience of at Least One Adverse Event (Table 40)
  - All Adverse Events (AEs) will be tabulated using Frequency tables with – Number of Incidents, Number of Subjects and Percentage of Subjects by:

        - Body System, Preferred Term and Resolution Time (within 30 days, within 31-90 days, more than 90 days), separately for Adverse Events started within 30 days post treatment, 31-90 days post treatment and more than 90 days post treatment (Table 41, Table 42, Table 43). Note that in case resolution date is missing, AE will be assigned to "Unresolved" category.
        - Body System, Preferred Term and Severity (Table 44)
        - Body System, Preferred Term and Relation to Treatment (Table 45)
    - Serious Adverse Events (SAEs):
        - Listing of all SAEs (Table 46)
        - In case the number of SAEs is greater than 12, all AE tables will be repeated, presenting SAEs only
    - The Kaplan-Meier curve of AE's free percentage of subjects by time will be presented by treatment group. The groups will be compared using a Log-Rank test.

10.6 Efficacy

10.6.1 Adjustment for Multiple Comparisons

This study has one primary and three secondary efficacy confirmatory endpoints. In order to control for multiplicity across the confirmatory endpoints, we will employ a hierarchical testing design. The primary efficacy analysis will be performed with a significance level of alpha = 0.05. Dependent on the successful confirmatory testing of the primary efficacy analysis, we will proceed with testing each of the three secondary efficacy confirmatory endpoints in the order listed in Section 6.2.2, proceeding with each test with alpha = 0.05 if all previous tests were successful. No confirmatory statements will be made about endpoints that are listed lower on the hierarchy after an endpoint fails testing. This way we will control the Type 1 error across all endpoints tested in this study.

10.6.2 Confirmatory Primary Efficacy

Primary efficacy analyses will be conducted on the ITT analysis population and will test the following hypothesis:

H_0: $M3_{ExAblate} \leq M3_{Sham}$

H_1: $M3_{ExAblate} > M3_{Sham}$

Where, $M3_{ExAblate}$ and $M3_{Sham}$ are means of Primary Endpoint (PE), as defined in Section 6.2.1, in the ExAblate and Sham groups, respectively.

This hypothesis will be analyzed using independent groups t-test with two-sided alpha=0.05, should the data not differ appreciably from normal theory. Otherwise, the Wilcoxon rank-sum test will be applied.

We will have succeeded if the Null is rejected and mean PE is higher in ExAblate than Sham. Descriptive statistics of the primary endpoint and comparison p-value will be provided (Table 47).

10.6.3 Confirmatory Secondary Efficacy

As indicated in Section 6.2.2, this study has three confirmatory secondary endpoints (SE1, SE2 and SE3). Hierarchical testing design will be applied to control for multiple statistical testing (as explained in Section 10.6.1).

<u>SE1 Analysis:</u>

The first confirmatory secondary efficacy analysis will be conducted on the ITT analysis population. The following hypothesis will be tested for SE1:

H_0: $Q3_{ExAblate} \leq Q3_{Sham}$

H_1: $Q3_{ExAblate} > Q3_{Sham}$

Where, $Q3_{ExAblate}$ and $Q3_{Sham}$ are means of the first Secondary Endpoint (SE1), as defined in Section 6.2.2, in the ExAblate and Sham groups, respectively.

This hypothesis will be analyzed using independent groups t-test with two-sided alpha=0.05, should the data not differ appreciably from normal theory. Otherwise, the Wilcoxon rank-sum test will be applied. Descriptive statistics and comparison p-value will be provided (Table 48).

SE2 Analysis:

The second confirmatory secondary efficacy analysis will be conducted on the ITT (subjects from ExAblate group only) population. The following hypothesis will be tested for SE2:

H_0: $M12_{ExAblate} \le 0$

H_1: $M12_{ExAblate} > 0$

Where, $M12_{ExAblate}$ is the mean of the second Secondary Endpoint (SE2), as defined in Section 6.2.2, in the ExAblate group.

This hypothesis will be analyzed using one-sample t-test with two-sided Alpha = 0.05, should the data not differ appreciably from normal theory. Otherwise, the one-sample Wilcoxon signed rank test will be applied. Descriptive statistics and comparison p-value will be provided (Table 49).

SE3 Analysis:

The third confirmatory secondary efficacy analysis will be conducted on the ITT analysis population. The following hypothesis will be tested for SE3:

H_0: $C3_{ExAblate} \le C3_{Sham}$

H_1: $C3_{ExAblate} > C3_{Sham}$

Where, $C3_{ExAblate}$ and $C3_{Sham}$ are means of the third Secondary Endpoint (SE3), as defined in Section 6.2.2, in the ExAblate and Sham groups, respectively.

This hypothesis will be analyzed using independent groups t-test with two-sided alpha=0.05, should the data not differ appreciably from normal theory. Otherwise, the Wilcoxon rank-sum test will be applied. Descriptive statistics and comparison p-value will be provided (Table 50).

10.6.4 Additional Secondary Efficacy

Additional secondary efficacy analyses will repeat those in the preceding sections (confirmatory primary and secondary efficacy analyses), when using PP analysis population instead of ITT (Table 51, Table 52, Table 53, Table 54).

Analysis of additional secondary efficacy endpoints (defined in Section 6.2.3) will be conducted on both ITT and PP analysis populations.

Descriptive statistics of raw values and change or percent change from baseline, as appropriate, over time by treatment group will be provided for the following:

- Clinical Rating Scale for Tremor (CRST) Parameters:
  - Part A and B CRST, separately for treated and non-treated side of the body (Table 55, Table 56)
  - Part B Score for treated side of the body, for each motor task (Table 57 – Table 61)
  - Part C Score for each item and Overall (Table 62 – Table 70)
  - Overall CRST Score (Table 71)
- Quality of Life in Essential Tremor Questionnaire (QUEST) Parameters:
  - Overall Quality of Life Score (Table 72)
  - Waking Hours with Tremor in Any Body Part (Table 73)
  - Tremor Severity Score, separately for each category (Table 74 – Table 79)
  - QUEST Parameters (based on questions 1-30):
    - Dimension Score, separately for each dimension (Table 80 – Table 84)
    - Summary of Dimensions Total Score (Table 85)

In addition, where appropriate, the following will be provided:

- Graphical presentation over time by treatment group
- Statistical testing to compare:
  - between treatment groups, by visit
  - within each treatment group, comparing Baseline to each follow-up visit
  - between treated and non-treated sides, within each treatment group, by visit

10.6.5 Covariate Analyses

The effects of covariates will be assessed for all confirmatory primary and secondary efficacy analyses.

The following covariates will be examined:

- Age
- Baseline CRST Score ($CRST_{[contralateral, Baseline]}$)
- Gender
- Center

Center is expected to have several categories. Keeping the number of categories as is will, in some cases, yield small subgroups and substantially reduce power. To address this issue, categories will be combined by grouping those with few observations into a single category. Grouping of categories will be specified after category frequency have been produced but before statistical testing.

The following will be provided:

1. Analyses for PE, SE1 and SE3 will be conducted on ITT analysis population comparing ExAblate and Sham groups. The following model will be applied:

   *Y = Covariate + Treatment Group+ Covariate*Treatment Group*

   Where,

   Y = PE, SE1 or SE3, depending on the analysis

   Covariate = covariate of interest

   *Covariate*Treatment Group*= covariate by group interaction

   Models both with and without *Covariate*Treatment Group* interaction will be considered (Table 89).

   Statistical testing will be done by linear regression. In case the distribution of Y is extremely not normal, the non-parametric methods will be applied depending on the emerged distribution.

   Descriptive statistics of each endpoint will be presented by covariate levels and treatment group. For this purpose numerical covariates (Age and Baseline CRST Score) will be categorized into equal subgroups inside the emerged range of values (Table 86, Table 87, Table 88).

   Assessing the effect of a covariate on treatment will be done by examining the degree to which the significance of the Treatment Effect changes with the inclusion of the covariate (Table 89). Weakening or strengthening of the Treatment Effect in the presence of a covariate will be interpreted using both clinical and statistical considerations.

2. Analyses for SE2 will be conducted on the ITT (subjects from ExAblate group only) population and will repeat the second confirmatory secondary efficacy analysis for each level of covariate separately. For this purpose numeric covariates (Age and Baseline CRST Score) will be categorized into equal subgroups inside the emerged range of values.
   Descriptive statistics of SE2 will be presented by covariate levels (Table 90). In addition, for numeric covariates a scatter plot of SE2 versus covariate will be produced (Figure 2, Figure 3). If no trend is observed, as shown on mock figures, the conclusion would be that the covariate has no effect on SE2.

10.6.6 Sensitivity Analyses

The method for imputation of missing data for the confirmatory primary efficacy analysis is defined as Last Observation Carried Forward (LOCF). In the case that there is a large amount of missing data, additional methods of imputing missing data will be used to examine the robustness of the treatment effect.
All sensitivity analyses will be conducted on the ITT analysis population and will repeat the confirmatory primary efficacy analysis.

Worst Case

Worst Case analysis will be assessed by imputing missing values as follows:

- The 10th percentile PE value of ExAblate group will be imputed to subjects with missing data in ExAblate group
- The 90th percentile PE value of Sham group will be imputed to subjects with missing data in Sham group

Descriptive statistics with comparison p-value will be provided (Table 91).

Best Case

Best Case analysis will be assessed by imputing missing values as follows:

- The 90th percentile PE value of ExAblate group will be imputed to subjects with missing data in ExAblate group
- The 10th percentile PE value of Sham group will be imputed to subjects with

missing data in Sham group

Descriptive statistics with comparison p-value will be provided (Table 92).

Multiple Imputations

Multiple Imputations analysis will be assessed by creating 10 imputed data sets using the multiple imputation procedure (PROC MI in SAS®). Various baseline characteristics will be considered for the purpose of imputation. Missing PE values will be imputed. The confirmatory primary efficacy analysis will be repeated on each of 10 imputed data sets (Table 93). The summarized result (using PROC MIANALYZE in SAS®) will be presented (Table 94).

10.7 Crossover Stage Analysis

The Crossover stage includes Sham subjects who terminated the Main stage of the study with the reason “Sham subject crossed over ExAblate Arm at Month 3”. According to the protocol, these subjects undergo Active ExAblate treatment and subsequent follow-up assessments, just as defined for the ExAblate group in the Main stage of the study.

All analyses in this section will be presented based on the Crossover analysis population. Results from the Main stage under Sham treatment will be presented in parallel with results under ExAblate treatment during Crossover stage, similarly to presentation of the Main stage results.

Descriptive statistics over time by treatment group will be provided for the following:

- Confirmatory primary and secondary efficacy endpoints
- Additional secondary endpoints

In addition, where appropriate, the following will be provided:

- Graphical presentation over time by treatment group
- Statistical testing to compare:
    - between treatment groups, by visit
    - within each treatment group, comparing Baseline to each follow-up visit

- between treated and non-treated sides, within each treatment group, by visit

11 Data Listings

Data listings will be provided for all data available.

12 Appendix

The following section presents mock tables and figures for Section 10.

12.1 Subject Disposition

Figure 1 Subject Disposition Flow Chart

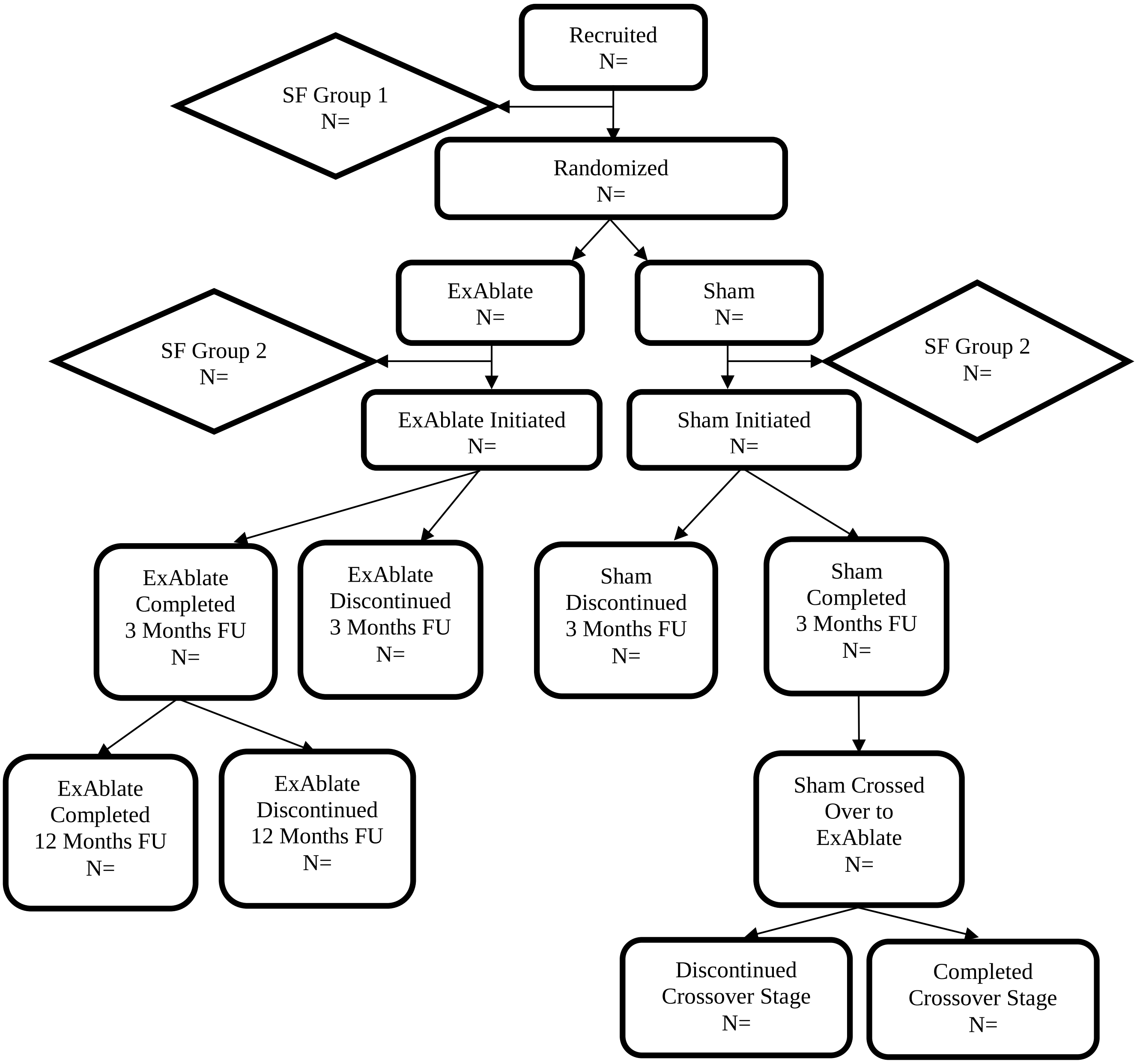

SF = Screen Failure

Table 1 Frequency Distribution of Answers to Inclusion Criteria

Number / Inclusion Criteria		Inclusion Answer				Total	
		Yes		No			
		N	%	N	%	N	%
1	Men and women, age 22 years and older						100
2	Subjects who are able and willing to give informed consent and able to attend all study visits						100
3	A diagnosis of ET as confirmed from clinical history and examination by a neurologist or neurosurgeon specialized in movement disorder						100
4	Tremor refractory to adequate trials of at least two medications, one of which should be a first line therapy of either propranolol or primidone						100
5	Following the 1-month medication stability period, subject must be on stable medication for tremor						100
6	Vim nucleus of thalamus can be target by the ExAblate device						100
7	Able to communicate sensations during the ExAblate MRgFUS treatment						100
8	Postural or intention tremor severity score of greater than or equal to 2 in the dominant hand/arm as measured by the CRST rating scale while stable on medication						100
9	May have bilateral appendicular tremor						100
10	Significant disability due to essential tremor despite medical treatment						100
11	Inclusion and exclusion criteria have been agreed upon by two members of the medical team						100
12	Subjects on stable antidepressant medications for at least 3 months may be enrolled into the study						100

Table 2 Frequency Distribution of Answers to Exclusion Criteria

Number / Exclusion Criteria		Exclusion Answer				Total	
		Yes		No			
		N	%	N	%	N	%
1	Subjects with unstable cardiac status						100
2	Subjects exhibiting any behavior(s) consistent with ethanol or substance abuse as defined by the criteria outlined in the DSM-IV						100
3	Severe hypertension (diastolic BP > 100 on medication)						100
4	Subjects with standard contraindications for MR imaging such as non-MRI compatible implanted metallic devices						100
5	Known intolerance or allergies to the MRI contrast agent (e.g. Gadolinium or Magnevist) including advanced kidney disease						100

Number / Exclusion Criteria		Exclusion Answer				Total	
		Yes		No			
		N	%	N	%	N	%
6	Patient with severely impaired renal function with estimated glomerular filtration rate <30 mL/min/1.73m^2 (or per local standards should that be more restrictive) and/or who is on dialysis						100
7	History of abnormal bleeding and/or coagulopathy						100
8	Receiving anticoagulant (e.g. warfarin) or antiplatelet (e.g. aspirin) therapy within one week of focused ultrasound procedure or drugs known to increase risk or hemorrhage (e.g. Avastin) within one month of focused ultrasound procedure						100
9	Active or suspected acute or chronic uncontrolled infection						100
10	History of immunocompromise including those who are HIV positive						100
11	History of intracranial hemorrhage						100
12	Cerebrovascular disease (multiple CVA or CVA within 6 months)						100
13	Subjects with uncontrolled symptoms and signs of increased intracranial pressure						100
14	Individuals who are not able or willing to tolerate the required prolonged stationary supine position during treatment						100
15	Are participating or have participated in another clinical trial in the last 30 days						100
16	Significant claustrophobia that cannot be managed with mild medication						100
17	Subjects unable to communicate with the investigator and staff						100
18	Presence of any other neurodegenerative disease such as Parkinson-plus syndromes suspected on neurological examination						100
19	Anyone suspected to have the diagnosis of idiopathic Parkinson’s disease						100
20	Presence of significant cognitive impairment as determined with a score ≤ 24 on the Mini Mental Status Examination (MMSE)						100
21	Subjects with life-threatening systemic disease						100
22	Subjects with a history of seizures within the past year						100
23	Subjects with presence or history of psychosis						100
24	Subjects with risk factors for intraoperative or postoperative bleeding						100
25	Subjects with brain tumors						100
26	Any illness that in the investigator's opinion preclude participation in this study						100
27	Pregnancy or lactation						100
28	Legal incapacity or limited legal capacity						100
29	Subjects who have had deep brain stimulation or a prior stereotactic ablation of the basal ganglia						100
30	Subjects who have been administered botulinum toxins into the arm, neck or face within 5 months prior to baseline						100

Table 3 Frequency Distribution of Overall Eligibility

Does the subject meet all inclusion/exclusion criteria?				Total	
Yes		No			
N	%	N	%	N	%

Table 4 Listing of Screen Failures

Line No.	Treatment Group	Subject ID	Reason for Screening Failure	Time of Screen Failure
1	SF Group-1			Before randomization
…	…			…
…	SF Group2 – ExAblate			After randomization but before the initiation of treatment
…	SF Group2 – Sham			After randomization but before the initiation of treatment
…	…			…

Table 5 Subject Accountability by Visit and Treatment Group

Category	Baseline		Week 1		Month 1		Month 3		Month 6	Month 12
	ExAblate	Sham	ExAblate	Sham	ExAblate	Sham	ExAblate	Sham	ExAblate	ExAblate
Consented										
SF 1[1]										
Randomized[2]										
SF 2[3]										
Theoretical[4]										
Death										
Failure[5]										
Expected[6]										
Actual[7]										
Actual %[8]										

1 - SF 1 – Those subjects consented, but not meeting enrollment criteria
2 - Randomized equals those consented minus SF 1
3 - SF 2 – Randomized subjects who have not received any sonication
4 - Theoretical is equal to the number of subjects consented minus SF 1 and 2. Therefore, theoretical is equal to the number of subjects eligible to receive treatment in either group
5 - Failures include any subjects (ExAblate or Sham) who discontinued study due to beginning another treatment for their condition.
6 - Expected equals Theoretical minus Death minus Failures
7 - Actual is the number of subjects actually returning for the follow-up visit
8 - Actual % is the number of Actual subjects divided by Expected

Table 6 By-Subject Listing of Protocol Deviations

Line No.	Treatment Group	Center	Subject ID	Deviation Category	Protocol Deviation
1					
2					
…					

Table 7 Number and Percentage of Subjects in Each of the Analysis Populations by Treatment Group (Safety)

Analysis Set / Status		Treatment Group			
		ExAblate		Sham	
		N	%	N	%
Safety	Included				
	Excluded				
	Total		100		100
ITT	Included				
	Excluded				
	Total		100		100
PP	Included				
	Excluded				
	Total		100		100

Table 8 Listing of Subjects Excluded from Each of the Analysis Populations (Safety)

Line No.	Analysis Population Excluded From	Treatment Group	Subject ID	Reasons for Exclusion from Analysis Population
1				
2				
3				
…				

Table 9 Number and Percentage of Subjects by Center and Treatment Group (ITT)

Center	Treatment Group			
	ExAblate		Sham	
	N	%	N	%
…				
…				
…				
Total		100		100

Table 10 Number and Percentage of Subjects Who Completed / Prematurely Discontinued the First 3 Months FU of the Study Main Stage by Treatment Group (ITT)

Subject Status in the Study	Treatment Group			
	ExAblate		Sham	
	N	%	N	%
Completed 3 Months FU				
Discontinued Prior to 3 Months FU				
Total		100		100

Table 11 Number and Percentage of Subjects Who Completed / Prematurely Discontinued the Follow Up Period of the Study Main Stage for ExAblate Group (ITT)

Subject Status in the Study	Treatment Group	
	ExAblate	
	N	%
Completed 12 Months FU		
Discontinued Prior to 12 Months FU		
Total		100

Table 12 Listing of All Dropouts (ITT)

Line No.	Last Visit	Treatment Group	Subject ID	Reason for Dropout
1				
2				
…				

Table 13 Crossover Stage: Number and Percentage of Subjects by Center (Crossover)

Center	N	%
…		
…		
…		
Total		100

Table 14 Crossover Stage: Number and Percentage of Subjects Who Completed / Prematurely Discontinued the Study Crossover Stage (Crossover)

Subject Status in the Study	N	%
Completed 12 Months FU		
Discontinued Prior to 12 Months FU		
Total		100

Table 15 Crossover Stage: Listing of All Dropouts (Crossover)

Line No.	Last Visit	Subject ID	Reason for Dropout
1			
2			
…			

Table 16 Crossover Stage: Subject Accountability by Visit

	Category	Treatment	Week 1	Month 1	Month 3	Month 6	Month 12
Theoretical[1]							
Death							
Failure[2]							
Expected[3]							
Actual[4]							
Actual %[5]							

1 - Theoretical is equal to the number of subjects received an active ExAblate treatment during the Crossover study stage
2 - Failures include any subjects who discontinued study due to beginning another treatment for their condition.
3 - Expected equals Theoretical minus Death minus Failures
4 - Actual is the number of subjects actually returning for the follow-up visit
5 - Actual % is the number of Actual subjects divided by Expected

12.2 Baseline Characteristics

Table 17 Demographic Characteristics: Descriptive Statistics of Age, BMI, Height and Weight by Treatment Group (Safety)

Demographic Characteristics		Treatment Group	
		ExAblate	Sham
Age [Years]	Mean		
	Std		
	Min		
	Median		
	Max		
	N		
BMI [kg/m^2]	Mean		
	Std		
	Min		
	Median		
	Max		
	N		
Height [cm]	Mean		
	Std		
	Min		
	Median		
	Max		
	N		
Weight [kg]	Mean		
	Std		
	Min		
	Median		
	Max		
	N		

Table 18 Demographic Characteristics: Frequency Distribution of Gender and Race by Treatment Group (Safety)

Demographic Characteristics		Treatment Group			
		ExAblate		Sham	
		N	%	N	%
Gender	Female				
	Male				
	Total		100		100
Race	Caucasian				
	Black				
	Asian				
	Hispanic				
	Other				
	Total		100		100

Table 19 Medical History: Frequency Distribution of Significant Medical Conditions (Other than the Present Disease) by Treatment Group (Safety)

Significant Medical Condition Present		Treatment Group			
		ExAblate		Sham	
		N	%	N	%
1. Chest Pain/Angina	Yes				
	No				
	Total		100		100
2. High Blood Pressure	Yes				
	No				
	Total		100		100
3. Heart Disease (including CHF)	Yes				
	No				
	Total		100		100
4. Heart Attack	Yes				
	No				
	Total		100		100
5. Pacemaker	Yes				
	No				
	Total		100		100

Significant Medical Condition Present		Treatment Group			
		ExAblate		Sham	
		N	%	N	%
6. Kidney Problems	Yes				
	No				
	Total		100		100
7. Diabetes Mellitus	Yes				
	No				
	Total		100		100
8. Bowel/Bladder Problems	Yes				
	No				
	Total		100		100
9. Asthma/Breathing Difficulties	Yes				
	No				
	Total		100		100
10. Liver Problems	Yes				
	No				
	Total		100		100
11. Seizures	Yes				
	No				
	Total		100		100
12. Metal Implants	Yes				
	No				
	Total		100		100
13. Dizziness/Fainting	Yes				
	No				
	Total		100		100
14. Severe Nausea/Vomiting (non-medication related)	Yes				
	No				
	Total		100		100
15. Hypoglycemia	Yes				
	No				
	Total		100		100
16. Back	Yes				
	No				
	Total		100		100

Significant Medical Condition Present		Treatment Group			
		ExAblate		Sham	
		N	%	N	%
17. Thyroid	Yes				
	No				
	Total		100		100
18. DVT	Yes				
	No				
	Total		100		100
19. Allergies	Yes				
	No				
	Total		100		100
20. Ethanol or Substance Abuse	Yes				
	No				
	Total		100		100
21. Mental Status	Yes				
	No				
	Total		100		100
22. Other Conditions	Yes				
	No				
	Total		100		100

Table 20 Medical History: Frequency Distribution of Other Significant Medical Conditions (Protocol Specific) by Treatment Group (Safety)

Significant Medical Condition Present		Treatment Group			
		ExAblate		Sham	
		N	%	N	%
1. Bleeding Disorders	Yes				
	No				
	Total		100		100
2. Intracranial Hemorrhage	Yes				
	No				
	Total		100		100
3. Stroke	Yes				
	No				
	Total		100		100

Significant Medical Condition Present		Treatment Group			
		ExAblate		Sham	
		N	%	N	%
4. Brain Tumors	Yes				
	No				
	Total		100		100
5. Positive HIV Status	Yes				
	No				
	Total		100		100
6. Alzheimer’s	Yes				
	No				
	Total		100		100
7. Parkinson’s Disease	Yes				
	No				
	Total		100		100

Table 21 Essential Tremors History: Descriptive Statistics of Time from Initial ET Symptoms, Time from Initial ET Diagnosis and Time from First ET Medical Therapy by Treatment Group (Safety)

Essential Tremors History		Treatment Group	
		ExAblate	Sham
Time from Initial ET Symptoms [Years]	Mean		
	Std		
	Min		
	Median		
	Max		
	N		
Time from Initial ET Diagnosis [Years]	Mean		
	Std		
	Min		
	Median		
	Max		
	N		
Time from First ET Medical Therapy	Mean		
	Std		

Essential Tremors History		Treatment Group	
		ExAblate	Sham
[Years]	Min		
	Median		
	Max		
	N		

Table 22 Essential Tremors History: Frequency Distribution of Family History of ET, Indication of Etiology due to Neuroleptic Drug Exposure and Is Subject Considered Medication Refractory by Treatment Group (Safety)

Essential Tremors History Present		Treatment Group			
		ExAblate		Sham	
		N	%	N	%
Family History of ET	Yes				
	No				
	Total		100		100
Is there indication of etiology due to neuroleptic drug exposure?	Yes				
	No				
	Total		100		100
Is the subject considered medication refractory?	Yes				
	No				
	Total		100		100

Table 23 Listing of Subjects Having Family History of ET (Safety)

Line No.	Subject ID	Relationship Degree and the Number of Relatives
1		
2		
…		

Table 24 Frequency Distribution of MRI Examination Results by Treatment Group (Safety)

MRI Examination		Treatment Group			
		ExAblate		Sham	
		N	%	N	%
Does the patient have any arteriovenous malformations that require treatment?	Yes				
	No				
	Total		100		100
Does the patient have any aneurysms that require treatment?	Yes				
	No				
	Total		100		100
Does the patient have evidence of a recent hemorrhage?	Yes				
	No				
	Total		100		100

Table 25 Frequency Distribution of CT Examination Results by Treatment Group (Safety)

CT Examination		Treatment Group			
		ExAblate		Sham	
		N	%	N	%
Are there any calcifications present within the treated area?	Yes				
	No				
	Total		100		100
Does the patient have any implants within the skull or brain?	Yes				
	No				
	Total		100		100
Is the skull suitable for Treatment?	Yes				
	No				
	Total		100		100
Is there a history of DVT or systemic thrombosis?	Yes				
	No				
	Total		100		100
Evidence of acute thrombosis in lower extremities (for subjects who had a history of DVT or systemic thrombosis)	Yes				
	No				
	Total		100		100

Table 26 CRST and QUEST Questionnaire Parameters at Baseline by Treatment Group (Safety)

CRST and QUEST Parameters at Baseline		Treatment Group	
		ExAblate	Sham
The Treated (Contralateral) Upper Extremity CRST Sub Score	Mean		
	Std		
	Min		
	Median		
	Max		
	N		
QUEST Summary of Dimensions Total Score	Mean		
	Std		
	Min		
	Median		
	Max		
	N		
Functional Disabilities CRST Part C Total Score	Mean		
	Std		
	Min		
	Median		
	Max		
	N		

Table 27 Comparison of Baseline Characteristics between Treatment Groups (Safety)

Parameter	P-Value	Statistical Test Used
…	…	…

12.3 Treatment Procedure

Table 28 Frequency Distribution of Pre-Treatment Characteristics by Treatment Group (Safety)

Pre-Treatment Characteristics		Treatment Group			
		ExAblate		Sham	
		N	%	N	%
Head Condition	Normal				
	Abnormal				
	Total		100		100
Does subject have scars or lesions on head?	Yes				
	No				
	Total		100		100
Was the subject "off" medication for at least 12 hours prior to the treatment?	Yes				
	No				
	Total		100		100

Table 29 Descriptive Statistics for Vital Signs by Time Point and Treatment Group (Safety)

Vital Signs		Pre-Procedure		Intra-Procedure		Post-Procedure	
		ExAblate	Sham	ExAblate	Sham	ExAblate	Sham
Systolic Blood Pressure [mmHg]	Mean						
	Std						
	Min						
	Median						
	Max						
	N						
Diastolic Blood Pressure [mHg]	Mean						
	Std						
	Min						
	Median						
	Max						
	N						
Heart Rate [bpm]	Mean						
	Std						

Vital Signs		Pre-Procedure		Intra-Procedure		Post-Procedure	
		ExAblate	Sham	ExAblate	Sham	ExAblate	Sham
	Min						
	Median						
	Max						
	N						
O_2 Saturation [%]	Mean						
	Std						
	Min						
	Median						
	Max						
	N						

Table 30 Descriptive Statistics of Treatment Characteristics by Treatment Group (Safety)

Treatment Characteristics		Treatment Group	
		ExAblate	Sham
Time Inside Scanner [min]	Mean		
	Std		
	Min		
	Median		
	Max		
	N		
Sonication Treatment Time [min]	Mean		
	Std		
	Min		
	Median		
	Max		
	N		
Target Size [cm^3]	Mean		
	Std		
	Min		
	Median		
	Max		
	N		
Minimal Energy [J]	Mean		

Treatment Characteristics		Treatment Group	
		ExAblate	Sham
	Std		
	Min		
	Median		
	Max		
	N		
Maximal Energy [J]	Mean		
	Std		
	Min		
	Median		
	Max		
	N		
Number of Performed Sonications	Mean		
	Std		
	Min		
	Median		
	Max		
	N		

Table 31 Frequency Distribution of Treated Side of the Body by Treatment Group (Safety)

Treated Side of the Body	Treatment Group			
	ExAblate		Sham	
	N	%	N	%
Right				
Left				
Total		100		100

Table 32 Frequency Distribution of Procedure Interruption Occurrence by Treatment Group (Safety)

Procedure Interruption		Treatment Group			
		ExAblate		Sham	
		N	%	N	%
Was the procedure interrupted for more than 30 minutes due to MR system problems?	Yes				
	No				
	Total		100		100
Was the procedure interrupted for more than 30 minutes due to ExAblate system problems?	Yes				
	No				
	Total		100		100

Table 33 Listing of All Device Malfunctions (Safety)

Line No.	Treatment Group	Subject ID	Malfunction Number	Device Failure / Malfunction of	How long was the delay? [min]	Was Treatment Completed?	For ExAblate Failure: Did resolution require InSightec personnel visit?	Failure / Malfunction Resolution Description
1	ExAblate		1	ExAblate Device / MR Scanner				
2			2					
3	Sham		1					
…								

Table 34 Frequency Distribution of Procedure Termination Prior to Completion by Treatment Group (Safety)

Procedure Terminated Prior to Completion	Treatment Group			
	ExAblate		Sham	
	N	%	N	%
Yes				
No				
Total		100		100

Table 35 Listing of Reasons for Procedure Termination Prior to Completion (Safety)

Line No.	Treatment Group	Subject ID	Reason for Procedure Termination Prior to Completion
1	ExAblate		
…			
…	Sham		
…			

12.4 Blinding Assessment

Table 36 Frequency Distribution of Subjective Perception of Treatment Received by Visit and Treatment Group (Safety)

Assessment / Visit / Response to Blinding Question			Treatment Group				P-Value
			ExAblate		Sham		
			N	%	N	%	
Subject Perception	Treatment	Actual					
		Placebo					
		Total		100		100	
	1 Week FU	Actual					
		Placebo					
		Total		100		100	
	1 Month FU	Actual					
		Placebo					
		Total		100		100	
	3 Months FU	Actual					
		Placebo					
		Total		100		100	
Site Assessor Perception	1 Month FU	Actual					
		Placebo					
		Total		100		100	
	3 Months FU	Actual					
		Placebo					
		Total		100		100	
Core Lab Reviewer Perception	Baseline	Actual					
		Placebo					
		Total		100		100	
	1 Month FU	Actual					
		Placebo					
		Total		100		100	
	3 Months FU	Actual					
		Placebo					
		Total		100		100	

Table 37 Frequency Distribution of Core Lab Reviewer Subjective Perception of Treatment Received by Visit for ExAblate Group (Safety)

Assessment / Visit / Response to Blinding Question			Treatment Group	
			ExAblate	
			N	%
Core Lab Reviewer Perception	6 Months FU	Actual		
		Placebo		
		Total		100
	12 Months FU	Actual		
		Placebo		
		Total		100

Table 38 Frequency Distribution of Core Lab Reviewer Subjective Perception of Treatment Received during Crossover Stage by Visit for ExAblate Group (Crossover)

Assessment / Visit / Response to Blinding Question			Treatment Group	
			ExAblate	
			N	%
Core Lab Reviewer Perception	1 Month FU	Actual		
		Placebo		
		Total		100
	3 Month FU	Actual		
		Placebo		
		Total		100
	6 Months FU	Actual		
		Placebo		
		Total		100
	12 Months FU	Actual		
		Placebo		
		Total		100

12.5 Safety

Table 39 Descriptive Statistics of Number of Adverse Events per Subject by Treatment Group (Safety)

Number of AEs per Subject	Treatment Group	
	ExAblate	Sham
Mean		
Std		
Min		
Median		
Max		
N		

Table 40 Frequency Distribution of Experience of at Least One Adverse Event by Treatment Group (Safety)

Experience of at Least One Adverse Event	Treatment Group			
	ExAblate		Sham	
	N	%	N	%
Yes				
No				
Total		100		100

Table 41 Adverse Events Started within 30 Days Post Treatment by Body System, Preferred Term, Resolution Time and Treatment Group (Safety)

Body System / Preferred Term / Resolution Time			ExAblate	Sham
Any	Any	Any	Number of incidents; Number of subjects (% of subjects)	
		<= 30 Days		
		31-90 Days		
		> 90 Days		
…	Any	Any		
		<= 30 Days		
		31-90 Days		
		> 90 Days		
…	…	Any		

Body System / Preferred Term / Resolution Time			ExAblate	Sham
		<= 30 Days		
		31-90 Days		
		> 90 Days		

Table 42 Adverse Events Started within 31-90 Days Post Treatment by Body System, Preferred Term, Resolution Time and Treatment Group (Safety)

Body System / Preferred Term / Resolution Time			ExAblate	Sham
Any	Any	Any	Number of incidents; Number of subjects (% of subjects)	
		<= 30 Days		
		31-90 Days		
		> 90 Days		
…	Any	Any		
		<= 30 Days		
		31-90 Days		
		> 90 Days		
…	…	Any		
		<= 30 Days		
		31-90 Days		
		> 90 Days		

Table 43 Adverse Events Started More than 90 Days Post Treatment by Body System, Preferred Term, Resolution Time for ExAblate Group (Safety)

Body System / Preferred Term / Resolution Time			ExAblate
Any	Any	Any	Number of incidents; Number of subjects (% of subjects)
		<= 30 Days	
		31-90 Days	
		> 90 Days	
…	Any	Any	
		<= 30 Days	
		31-90 Days	
		> 90 Days	
…	…	Any	
		<= 30 Days	

Body System / Preferred Term / Resolution Time			ExAblate
		31-90 Days	
		> 90 Days	

Table 44 Adverse Events by Body System, Preferred Term, Severity and Treatment Group (Safety)

Body System / Preferred Term / Severity			ExAblate	Sham
Any	Any	Any	Number of incidents; Number of subjects (% of subjects)	
		Mild		
		Moderate		
		Severe		
		Life-threatening		
…	Any	Any		
		Mild		
		Moderate		
		Severe		
		Life-threatening		
…	…	Any		
		Mild		
		Moderate		
		Severe		
		Life-threatening		

Table 45 Adverse Events by Body System, Preferred Term, Relation to Treatment and Treatment Group (Safety)

Body System / Preferred Term / Relation to Treatment			ExAblate	Sham
Any	Any	Any	Number of incidents; Number of subjects (% of subjects)	
		…		
		…		
…	Any	Any		
		…		
		…		
…	…	Any		
		…		
		…		

Table 46 Listing of Serious Adverse Events by Treatment Group (Safety)

Line No.	Treatment Group	Subject	Date Treated	SAE Start Date	SAE Stop Date	Reason Serious	Severity	Relation	Action	Outcome	Incident Description	Treatment Description
1												
…												
…												

12.6 Efficacy

Table 47 Confirmatory Primary Efficacy (PE): Descriptive Statistics of Percent Improvement from Baseline at Three Months Post-Treatment in the Treated (Contralateral) Upper Extremity CRST Sub Score by Treatment Group (ITT)

PE	Treatment Group		P-Value
	ExAblate	Sham	
Mean			
Std			
Min			
Median			
Max			
N			

Table 48 Confirmatory Secondary Efficacy (SE1): Descriptive Statistics of Improvement from Baseline at Three Months Post-Treatment in Quality of Life in Essential Tremor (QUEST) Outcome by Treatment Group (ITT)

SE1	Treatment Group		P-Value
	ExAblate	Sham	
Mean			
Std			
Min			
Median			
Max			
N			

Table 49 Confirmatory Secondary Efficacy (SE2): Descriptive Statistics of Percent Improvement from Baseline at Twelve Months Post-Treatment in the Treated (Contralateral) Upper Extremity CRST Sub Score in ExAblate Group (ITT)

SE2	Treatment Group	P-Value
	ExAblate	
Mean		
Std		
Min		
Median		
Max		
N		

Table 50 Confirmatory Secondary Efficacy (SE3): Descriptive Statistics of Percent Improvement from Baseline at Three Months Post-Treatment in Functional Disabilities Total Score, as Measured by CRST Part-C by Treatment Group (ITT)

SE3	Treatment Group		P-Value
	ExAblate	Sham	
Mean			
Std			
Min			
Median			
Max			
N			

Table 51 Additional Secondary Efficacy (PE): Descriptive Statistics of Percent Improvement from Baseline at Three Months Post-Treatment in the Treated (Contralateral) Upper Extremity CRST Sub Score by Treatment Group (PP)

PE	Treatment Group		P-Value
	ExAblate	Sham	
Mean			
Std			
Min			
Median			
Max			
N			

Table 52 Additional Secondary Efficacy (SE1): Descriptive Statistics of Improvement from Baseline at Three Months Post-Treatment in Quality of Life in Essential Tremor (QUEST) Outcome by Treatment Group (PP)

SE1	Treatment Group		P-Value
	ExAblate	Sham	
Mean			
Std			
Min			
Median			
Max			
N			

Table 53 Additional Secondary Efficacy (SE2): Descriptive Statistics of Percent Improvement from Baseline at Twelve Months Post-Treatment in the Treated (Contralateral) Upper Extremity CRST Sub Score in ExAblate Group (PP)

SE2	Treatment Group	P-Value
	ExAblate	
Mean		
Std		
Min		
Median		
Max		
N		

Table 54 Additional Secondary Efficacy (SE3): Descriptive Statistics of Percent Improvement from Baseline at Three Months Post-Treatment in Functional Disabilities Total Score, as Measured by CRST Part-C by Treatment Group (PP)

SE3	Treatment Group		P-Value
	ExAblate	Sham	
Mean			
Std			
Min			
Median			
Max			
N			

Table 55 Additional Secondary Efficacy: Descriptive Statistics of Part A and B CRST over Time by Side of the Body and Treatment Group (ITT)

Visit / Part A and B CRST		Raw Values				Change from Baseline			
		Treated Side		Non-Treated Side		Treated Side		Non-Treated Side	
		ExAblate	Sham	ExAblate	Sham	ExAblate	Sham	ExAblate	Sham
Baseline	Mean					NA	NA	NA	NA
	Std					NA	NA	NA	NA
	N					NA	NA	NA	NA
1 Month FU	Mean								
	Std								
	N								
3 Months FU	Mean								
	Std								
	N								
6 Months FU	Mean		NA		NA		NA		NA
	Std		NA		NA		NA		NA
	N		NA		NA		NA		NA
12 Months FU	Mean		NA		NA		NA		NA
	Std		NA		NA		NA		NA
	N		NA		NA		NA		NA

Table 56 Additional Secondary Efficacy: Descriptive Statistics of Part A and B CRST over Time by Side of the Body and Treatment Group (PP)

Visit / Part A and B CRST		Raw Values				Change from Baseline			
		Treated Side		Non-Treated Side		Treated Side		Non-Treated Side	
		ExAblate	Sham	ExAblate	Sham	ExAblate	Sham	ExAblate	Sham
Baseline	Mean					NA	NA	NA	NA
	Std					NA	NA	NA	NA
	N					NA	NA	NA	NA
1 Month FU	Mean								
	Max								
	N								
3 Months FU	Mean								
	Std								
	N								

Visit / Part A and B CRST		Raw Values				Change from Baseline			
		Treated Side		Non-Treated Side		Treated Side		Non-Treated Side	
		ExAblate	Sham	ExAblate	Sham	ExAblate	Sham	ExAblate	Sham
6 Months FU	Mean		NA		NA		NA		NA
	Std		NA		NA		NA		NA
	N		NA		NA		NA		NA
12 Months FU	Mean		NA		NA		NA		NA
	Std		NA		NA		NA		NA
	N		NA		NA		NA		NA

Table 57 Additional Secondary Efficacy: Descriptive Statistics of Part B Score for Treated Side of the Body for Handwriting over Time by Treatment Group and Analysis Population (ITT, PP)

Visit / Part B Score for Handwriting		ITT				PP			
		Raw Values		Change from Baseline		Raw Values		Change from Baseline	
		ExAblate	Sham	ExAblate	Sham	ExAblate	Sham	ExAblate	Sham
Baseline	Mean					NA	NA	NA	NA
	Std					NA	NA	NA	NA
	N					NA	NA	NA	NA
1 Month FU	Mean								
	Std								
	N								
3 Months FU	Mean								
	Std								
	N								
6 Months FU	Mean		NA		NA		NA		NA
	Std		NA		NA		NA		NA
	N		NA		NA		NA		NA
12 Months FU	Mean		NA		NA		NA		NA
	Std		NA		NA		NA		NA
	N		NA		NA		NA		NA

Table 58 Additional Secondary Efficacy: Descriptive Statistics of Part B Score for Treated Side of the Body for Drawing A over Time by Treatment Group and Analysis Population (ITT, PP)

Visit / Part B Score for Drawing A		ITT				PP			
		Raw Values		Change from Baseline		Raw Values		Change from Baseline	
		ExAblate	Sham	ExAblate	Sham	ExAblate	Sham	ExAblate	Sham
Baseline	Mean					NA	NA	NA	NA
	Std					NA	NA	NA	NA
	N					NA	NA	NA	NA
1 Month FU	Mean								
	Std								
	N								
3 Months FU	Mean								
	Std								
	N								
6 Months FU	Mean		NA		NA		NA		NA
	Std		NA		NA		NA		NA
	N		NA		NA		NA		NA
12 Months FU	Mean		NA		NA		NA		NA
	Std		NA		NA		NA		NA
	N		NA		NA		NA		NA

Table 59 Additional Secondary Efficacy: Descriptive Statistics of Part B Score for Treated Side of the Body for Drawing B over Time by Treatment Group and Analysis Population (ITT, PP)

Visit / Part B Score for Drawing B		ITT				PP			
		Raw Values		Change from Baseline		Raw Values		Change from Baseline	
		ExAblate	Sham	ExAblate	Sham	ExAblate	Sham	ExAblate	Sham
Baseline	Mean					NA	NA	NA	NA
	Std					NA	NA	NA	NA
	N					NA	NA	NA	NA
1 Month FU	Mean								
	Std								
	N								
3 Months	Mean								

Visit / Part B Score for Drawing B		ITT				PP			
		Raw Values		Change from Baseline		Raw Values		Change from Baseline	
		ExAblate	Sham	ExAblate	Sham	ExAblate	Sham	ExAblate	Sham
FU	Std								
	N								
6 Months FU	Mean		NA		NA		NA		NA
	Std		NA		NA		NA		NA
	N		NA		NA		NA		NA
12 Months FU	Mean		NA		NA		NA		NA
	Std		NA		NA		NA		NA
	N		NA		NA		NA		NA

Table 60 Additional Secondary Efficacy: Descriptive Statistics of Part B Score for Treated Side of the Body for Drawing C over Time by Treatment Group and Analysis Population (ITT, PP)

Visit / Part B Score for Drawing C		ITT				PP			
		Raw Values		Change from Baseline		Raw Values		Change from Baseline	
		ExAblate	Sham	ExAblate	Sham	ExAblate	Sham	ExAblate	Sham
Baseline	Mean					NA	NA	NA	NA
	Std					NA	NA	NA	NA
	N					NA	NA	NA	NA
1 Month FU	Mean								
	Std								
	N								
3 Months FU	Mean								
	Std								
	N								
6 Months FU	Mean		NA		NA		NA		NA
	Std		NA		NA		NA		NA
	N		NA		NA		NA		NA
12 Months FU	Mean		NA		NA		NA		NA
	Std		NA		NA		NA		NA
	N		NA		NA		NA		NA

Table 61 Additional Secondary Efficacy: Descriptive Statistics of Part B Score for Treated Side of the Body for Pouring over Time by Treatment Group and Analysis Population (ITT, PP)

Visit / Part B Score for Pouring		ITT				PP			
		Raw Values		Change from Baseline		Raw Values		Change from Baseline	
		ExAblate	Sham	ExAblate	Sham	ExAblate	Sham	ExAblate	Sham
Baseline	Mean					NA	NA	NA	NA
	Std					NA	NA	NA	NA
	N					NA	NA	NA	NA
1 Month FU	Mean								
	Std								
	N								
3 Months FU	Mean								
	Std								
	N								
6 Months FU	Mean		NA		NA		NA		NA
	Std		NA		NA		NA		NA
	N		NA		NA		NA		NA
12 Months FU	Mean		NA		NA		NA		NA
	Std		NA		NA		NA		NA
	N		NA		NA		NA		NA

Table 62 Additional Secondary Efficacy: Descriptive Statistics of Part C Score for Speaking over Time by Treatment Group and Analysis Population (ITT, PP)

Visit / Part C Score for Speaking		ITT				PP			
		Raw Values		Change from Baseline		Raw Values		Change from Baseline	
		ExAblate	Sham	ExAblate	Sham	ExAblate	Sham	ExAblate	Sham
Baseline	Mean			NA	NA			NA	NA
	Std			NA	NA			NA	NA
	N			NA	NA			NA	NA
1 Month FU	Mean								
	Std								
	N								
3 Months	Mean								
	Std								

Visit / Part C Score for Speaking		ITT				PP			
		Raw Values		Change from Baseline		Raw Values		Change from Baseline	
		ExAblate	Sham	ExAblate	Sham	ExAblate	Sham	ExAblate	Sham
FU	N								
6 Months FU	Mean		NA		NA		NA		NA
	Std		NA		NA		NA		NA
	N		NA		NA		NA		NA
12 Months FU	Mean		NA		NA		NA		NA
	Std		NA		NA		NA		NA
	N		NA		NA		NA		NA

Table 63 Additional Secondary Efficacy: Descriptive Statistics of Part C Score for Eating over Time by Treatment Group and Analysis Population (ITT, PP)

Visit / Part C Score for Eating		ITT				PP			
		Raw Values		Change from Baseline		Raw Values		Change from Baseline	
		ExAblate	Sham	ExAblate	Sham	ExAblate	Sham	ExAblate	Sham
Baseline	Mean			NA	NA			NA	NA
	Std			NA	NA			NA	NA
	N			NA	NA			NA	NA
1 Month FU	Mean								
	Std								
	N								
3 Months FU	Mean								
	Std								
	N								
6 Months FU	Mean		NA		NA		NA		NA
	Std		NA		NA		NA		NA
	N		NA		NA		NA		NA
12 Months FU	Mean		NA		NA		NA		NA
	Std		NA		NA		NA		NA
	N		NA		NA		NA		NA

Table 64 Additional Secondary Efficacy: Descriptive Statistics of Part C Score for Drinking over Time by Treatment Group and Analysis Population (ITT, PP)

Visit / Part C Score for Drinking		ITT				PP			
		Raw Values		Change from Baseline		Raw Values		Change from Baseline	
		ExAblate	Sham	ExAblate	Sham	ExAblate	Sham	ExAblate	Sham
Baseline	Mean			NA	NA			NA	NA
	Std			NA	NA			NA	NA
	N			NA	NA			NA	NA
1 Month FU	Mean								
	Std								
	N								
3 Months FU	Mean								
	Std								
	N								
6 Months FU	Mean		NA		NA		NA		NA
	Std		NA		NA		NA		NA
	N		NA		NA		NA		NA
12 Months FU	Mean		NA		NA		NA		NA
	Std		NA		NA		NA		NA
	N		NA		NA		NA		NA

Table 65 Additional Secondary Efficacy: Descriptive Statistics of Part C Score for Hygiene over Time by Treatment Group and Analysis Population (ITT, PP)

Visit / Part C Score for Hygiene		ITT				PP			
		Raw Values		Change from Baseline		Raw Values		Change from Baseline	
		ExAblate	Sham	ExAblate	Sham	ExAblate	Sham	ExAblate	Sham
Baseline	Mean			NA	NA			NA	NA
	Std			NA	NA			NA	NA
	N			NA	NA			NA	NA
1 Month FU	Mean								
	Std								
	N								
3 Months FU	Mean								
	Std								
	N								

Visit / Part C Score for Hygiene		ITT				PP			
		Raw Values		Change from Baseline		Raw Values		Change from Baseline	
		ExAblate	Sham	ExAblate	Sham	ExAblate	Sham	ExAblate	Sham
6 Months FU	Mean		NA		NA		NA		NA
	Std		NA		NA		NA		NA
	N		NA		NA		NA		NA
12 Months FU	Mean		NA		NA		NA		NA
	Std		NA		NA		NA		NA
	N		NA		NA		NA		NA

Table 66 Additional Secondary Efficacy: Descriptive Statistics of Part C Score for Dressing over Time by Treatment Group and Analysis Population (ITT, PP)

Visit / Part C Score for Dressing		ITT				PP			
		Raw Values		Change from Baseline		Raw Values		Change from Baseline	
		ExAblate	Sham	ExAblate	Sham	ExAblate	Sham	ExAblate	Sham
Baseline	Mean			NA	NA			NA	NA
	Std			NA	NA			NA	NA
	N			NA	NA			NA	NA
1 Month FU	Mean								
	Std								
	N								
3 Months FU	Mean								
	Std								
	N								
6 Months FU	Mean		NA		NA		NA		NA
	Std		NA		NA		NA		NA
	N		NA		NA		NA		NA
12 Months FU	Mean		NA		NA		NA		NA
	Std		NA		NA		NA		NA
	N		NA		NA		NA		NA

Table 67 Additional Secondary Efficacy: Descriptive Statistics of Part C Score for Writing over Time by Treatment Group and Analysis Population (ITT, PP)

Visit / Part C Score for Writing		ITT				PP			
		Raw Values		Change from Baseline		Raw Values		Change from Baseline	
		ExAblate	Sham	ExAblate	Sham	ExAblate	Sham	ExAblate	Sham
Baseline	Mean			NA	NA			NA	NA
	Std			NA	NA			NA	NA
	N			NA	NA			NA	NA
1 Month FU	Mean								
	Std								
	N								
3 Months FU	Mean								
	Std								
	N								
6 Months FU	Mean		NA		NA		NA		NA
	Std		NA		NA		NA		NA
	N		NA		NA		NA		NA
12 Months FU	Mean		NA		NA		NA		NA
	Std		NA		NA		NA		NA
	N		NA		NA		NA		NA

Table 68 Additional Secondary Efficacy: Descriptive Statistics of Part C Score for Working over Time by Treatment Group and Analysis Population (ITT, PP)

Visit / Part C Score for Working		ITT				PP			
		Raw Values		Change from Baseline		Raw Values		Change from Baseline	
		ExAblate	Sham	ExAblate	Sham	ExAblate	Sham	ExAblate	Sham
Baseline	Mean			NA	NA			NA	NA
	Std			NA	NA			NA	NA
	N			NA	NA			NA	NA
1 Month FU	Mean								
	Std								
	N								
3 Months FU	Mean								
	Std								
	N								

Visit / Part C Score for Working		ITT				PP			
		Raw Values		Change from Baseline		Raw Values		Change from Baseline	
		ExAblate	Sham	ExAblate	Sham	ExAblate	Sham	ExAblate	Sham
6 Months FU	Mean		NA		NA		NA		NA
	Std		NA		NA		NA		NA
	N		NA		NA		NA		NA
12 Months FU	Mean		NA		NA		NA		NA
	Std		NA		NA		NA		NA
	N		NA		NA		NA		NA

Table 69 Additional Secondary Efficacy: Descriptive Statistics of Part C Score for Social Activities over Time by Treatment Group and Analysis Population (ITT, PP)

Visit / Part C Score for Social Activities		ITT				PP			
		Raw Values		Change from Baseline		Raw Values		Change from Baseline	
		ExAblate	Sham	ExAblate	Sham	ExAblate	Sham	ExAblate	Sham
Baseline	Mean			NA	NA			NA	NA
	Std			NA	NA			NA	NA
	N			NA	NA			NA	NA
1 Month FU	Mean								
	Std								
	N								
3 Months FU	Mean								
	Std								
	N								
6 Months FU	Mean		NA		NA		NA		NA
	Std		NA		NA		NA		NA
	N		NA		NA		NA		NA
12 Months FU	Mean		NA		NA		NA		NA
	Std		NA		NA		NA		NA
	N		NA		NA		NA		NA

Table 70 Additional Secondary Efficacy: Descriptive Statistics of Part C Overall Score over Time by Treatment Group and Analysis Population (ITT, PP)

Visit / Part C Overall Score		ITT				PP			
		Raw Values		Change from Baseline		Raw Values		Change from Baseline	
		ExAblate	Sham	ExAblate	Sham	ExAblate	Sham	ExAblate	Sham
Baseline	Mean			NA	NA			NA	NA
	Std			NA	NA			NA	NA
	N			NA	NA			NA	NA
1 Month FU	Mean								
	Std								
	N								
3 Months FU	Mean								
	Std								
	N								
6 Months FU	Mean		NA		NA		NA		NA
	Std		NA		NA		NA		NA
	N		NA		NA		NA		NA
12 Months FU	Mean		NA		NA		NA		NA
	Std		NA		NA		NA		NA
	N		NA		NA		NA		NA

Table 71 Additional Secondary Efficacy: Descriptive Statistics of Overall CRST Score over Time by Treatment Group and Analysis Population (ITT, PP)

Visit / Overall CRST Score		ITT				PP			
		Raw Values		Change from Baseline		Raw Values		Change from Baseline	
		ExAblate	Sham	ExAblate	Sham	ExAblate	Sham	ExAblate	Sham
Baseline	Mean			NA	NA			NA	NA
	Std			NA	NA			NA	NA
	N			NA	NA			NA	NA
1 Month FU	Mean								
	Std								
	N								
3 Months FU	Mean								
	Std								
	N								

Visit / Overall CRST Score		ITT				PP			
		Raw Values		Change from Baseline		Raw Values		Change from Baseline	
		ExAblate	Sham	ExAblate	Sham	ExAblate	Sham	ExAblate	Sham
6 Months FU	Mean		NA		NA		NA		NA
	Std		NA		NA		NA		NA
	N		NA		NA		NA		NA
12 Months FU	Mean		NA		NA		NA		NA
	Std		NA		NA		NA		NA
	N		NA		NA		NA		NA

Table 72 Additional Secondary Efficacy: Descriptive Statistics of Overall Quality of Life Score over Time by Treatment Group and Analysis Population (ITT, PP)

Visit / Overall Quality of Life Score		ITT				PP			
		Raw Values		Change from Baseline		Raw Values		Change from Baseline	
		ExAblate	Sham	ExAblate	Sham	ExAblate	Sham	ExAblate	Sham
Baseline	Mean			NA	NA			NA	NA
	Std			NA	NA			NA	NA
	N			NA	NA			NA	NA
1 Month FU	Mean								
	Std								
	N								
3 Months FU	Mean								
	Std								
	N								
6 Months FU	Mean		NA		NA		NA		NA
	Std		NA		NA		NA		NA
	N		NA		NA		NA		NA
12 Months FU	Mean		NA		NA		NA		NA
	Std		NA		NA		NA		NA
	N		NA		NA		NA		NA

Table 73 Additional Secondary Efficacy: Descriptive Statistics of Waking Hours with Tremor in Any Body Part over Time by Treatment Group and Analysis Population (ITT, PP)

Visit / Waking Hours with Tremor in Any Body Part		ITT				PP			
		Raw Values		Change from Baseline		Raw Values		Change from Baseline	
		ExAblate	Sham	ExAblate	Sham	ExAblate	Sham	ExAblate	Sham
Baseline	Mean			NA	NA			NA	NA
	Std			NA	NA			NA	NA
	N			NA	NA			NA	NA
1 Month FU	Mean								
	Std								
	N								
3 Months FU	Mean								
	Std								
	N								
6 Months FU	Mean		NA		NA		NA		NA
	Std		NA		NA		NA		NA
	N		NA		NA		NA		NA
12 Months FU	Mean		NA		NA		NA		NA
	Std		NA		NA		NA		NA
	N		NA		NA		NA		NA

Table 74 Additional Secondary Efficacy: Descriptive Statistics of Tremor Severity Score for Head over Time by Treatment Group and Analysis Population (ITT, PP)

Visit / Tremor Severity Score for Head		ITT				PP			
		Raw Values		Change from Baseline		Raw Values		Change from Baseline	
		ExAblate	Sham	ExAblate	Sham	ExAblate	Sham	ExAblate	Sham
Baseline	Mean			NA	NA			NA	NA
	Std			NA	NA			NA	NA
	N			NA	NA			NA	NA
1 Month FU	Mean								
	Std								
	N								
3	Mean								

Visit / Tremor Severity Score for Head		ITT				PP			
		Raw Values		Change from Baseline		Raw Values		Change from Baseline	
		ExAblate	Sham	ExAblate	Sham	ExAblate	Sham	ExAblate	Sham
Months FU	Std								
	N								
6 Months FU	Mean		NA		NA		NA		NA
	Std		NA		NA		NA		NA
	N		NA		NA		NA		NA
12 Months FU	Mean		NA		NA		NA		NA
	Std		NA		NA		NA		NA
	N		NA		NA		NA		NA

Table 75 Additional Secondary Efficacy: Descriptive Statistics of Tremor Severity Score for Voice over Time by Treatment Group and Analysis Population (ITT, PP)

Visit / Tremor Severity Score for Voice		ITT				PP			
		Raw Values		Change from Baseline		Raw Values		Change from Baseline	
		ExAblate	Sham	ExAblate	Sham	ExAblate	Sham	ExAblate	Sham
Baseline	Mean			NA	NA			NA	NA
	Std			NA	NA			NA	NA
	N			NA	NA			NA	NA
1 Month FU	Mean								
	Std								
	N								
3 Months FU	Mean								
	Std								
	N								
6 Months FU	Mean		NA		NA		NA		NA
	Std		NA		NA		NA		NA
	N		NA		NA		NA		NA
12 Months FU	Mean		NA		NA		NA		NA
	Std		NA		NA		NA		NA
	N		NA		NA		NA		NA

Table 76 Additional Secondary Efficacy: Descriptive Statistics of Tremor Severity Score for Treated Arm/Hand over Time by Treatment Group and Analysis Population (ITT, PP)

Visit / Tremor Severity Score for Treated Arm/Hand		ITT				PP			
		Raw Values		Change from Baseline		Raw Values		Change from Baseline	
		ExAblate	Sham	ExAblate	Sham	ExAblate	Sham	ExAblate	Sham
Baseline	Mean			NA	NA			NA	NA
	Std			NA	NA			NA	NA
	N			NA	NA			NA	NA
1 Month FU	Mean								
	Std								
	N								
3 Months FU	Mean								
	Std								
	N								
6 Months FU	Mean		NA		NA		NA		NA
	Std		NA		NA		NA		NA
	N		NA		NA		NA		NA
12 Months FU	Mean		NA		NA		NA		NA
	Std		NA		NA		NA		NA
	N		NA		NA		NA		NA

Table 77 Additional Secondary Efficacy: Descriptive Statistics of Tremor Severity Score for Non-Treated Arm/Hand over Time by Treatment Group and Analysis Population (ITT, PP)

Visit / Tremor Severity Score for Non-Treated Arm/Hand		ITT				PP			
		Raw Values		Change from Baseline		Raw Values		Change from Baseline	
		ExAblate	Sham	ExAblate	Sham	ExAblate	Sham	ExAblate	Sham
Baseline	Mean			NA	NA			NA	NA
	Std			NA	NA			NA	NA
	N			NA	NA			NA	NA
1 Month FU	Mean								
	Std								
	N								
3	Mean								

Visit / Tremor Severity Score for Non-Treated Arm/Hand		ITT				PP			
		Raw Values		Change from Baseline		Raw Values		Change from Baseline	
		ExAblate	Sham	ExAblate	Sham	ExAblate	Sham	ExAblate	Sham
Months FU	Std								
	N								
6 Months FU	Mean		NA		NA		NA		NA
	Std		NA		NA		NA		NA
	N		NA		NA		NA		NA
12 Months FU	Mean		NA		NA		NA		NA
	Std		NA		NA		NA		NA
	N		NA		NA		NA		NA

Table 78 Additional Secondary Efficacy: Descriptive Statistics of Tremor Severity Score for Leg/Foot Contralateral to Brain Treatment over Time by Treatment Group and Analysis Population (ITT, PP)

Visit / Tremor Severity Score for Leg/Foot Contralateral to Brain Treatment		ITT				PP			
		Raw Values		Change from Baseline		Raw Values		Change from Baseline	
		ExAblate	Sham	ExAblate	Sham	ExAblate	Sham	ExAblate	Sham
Baseline	Mean			NA	NA			NA	NA
	Std			NA	NA			NA	NA
	N			NA	NA			NA	NA
1 Month FU	Mean								
	Std								
	N								
3 Months FU	Mean								
	Std								
	N								
6 Months FU	Mean		NA		NA		NA		NA
	Std		NA		NA		NA		NA
	N		NA		NA		NA		NA
12 Months FU	Mean		NA		NA		NA		NA
	Std		NA		NA		NA		NA
	N		NA		NA		NA		NA

Table 79 Additional Secondary Efficacy: Descriptive Statistics of Tremor Severity Score for Leg/Foot Ipsilateral to Brain Treatment over Time by Treatment Group and Analysis Population (ITT, PP)

Visit / Tremor Severity Score for Leg/Foot Ipsilateral to Brain Treatment		ITT				PP			
		Raw Values		Change from Baseline		Raw Values		Change from Baseline	
		ExAblate	Sham	ExAblate	Sham	ExAblate	Sham	ExAblate	Sham
Baseline	Mean			NA	NA			NA	NA
	Std			NA	NA			NA	NA
	N			NA	NA			NA	NA
1 Month FU	Mean								
	Std								
	N								
3 Months FU	Mean								
	Std								
	N								
6 Months FU	Mean		NA		NA		NA		NA
	Std		NA		NA		NA		NA
	N		NA		NA		NA		NA
12 Months FU	Mean		NA		NA		NA		NA
	Std		NA		NA		NA		NA
	N		NA		NA		NA		NA

Table 80 Additional Secondary Efficacy: Descriptive Statistics of QUEST Communication Score over Time by Treatment Group and Analysis Population (ITT, PP)

Visit / QUEST Communication Score		ITT				PP			
		Raw Values		Change from Baseline		Raw Values		Change from Baseline	
		ExAblate	Sham	ExAblate	Sham	ExAblate	Sham	ExAblate	Sham
Baseline	Mean			NA	NA			NA	NA
	Std			NA	NA			NA	NA
	N			NA	NA			NA	NA
1 Month FU	Mean								
	Std								
	N								
3	Mean								

Visit / QUEST Communication Score		ITT				PP			
		Raw Values		Change from Baseline		Raw Values		Change from Baseline	
		ExAblate	Sham	ExAblate	Sham	ExAblate	Sham	ExAblate	Sham
Months FU	Std								
	N								
6 Months FU	Mean		NA		NA		NA		NA
	Std		NA		NA		NA		NA
	N		NA		NA		NA		NA
12 Months FU	Mean		NA		NA		NA		NA
	Std		NA		NA		NA		NA
	N		NA		NA		NA		NA

Table 81 Additional Secondary Efficacy: Descriptive Statistics of QUEST Work and Finances Score over Time by Treatment Group and Analysis Population (ITT, PP)

Visit / QUEST Work and Finances Score		ITT				PP			
		Raw Values		Change from Baseline		Raw Values		Change from Baseline	
		ExAblate	Sham	ExAblate	Sham	ExAblate	Sham	ExAblate	Sham
Baseline	Mean			NA	NA			NA	NA
	Std			NA	NA			NA	NA
	N			NA	NA			NA	NA
1 Month FU	Mean								
	Std								
	N								
3 Months FU	Mean								
	Std								
	N								
6 Months FU	Mean		NA		NA		NA		NA
	Std		NA		NA		NA		NA
	N		NA		NA		NA		NA
12 Months FU	Mean		NA		NA		NA		NA
	Std		NA		NA		NA		NA
	N		NA		NA		NA		NA

Table 82 Additional Secondary Efficacy: Descriptive Statistics of QUEST Hobbies and Leisure Score over Time by Treatment Group and Analysis Population (ITT, PP)

Visit / QUEST Hobbies and Leisure Score		ITT				PP			
		Raw Values		Change from Baseline		Raw Values		Change from Baseline	
		ExAblate	Sham	ExAblate	Sham	ExAblate	Sham	ExAblate	Sham
Baseline	Mean			NA	NA			NA	NA
	Std			NA	NA			NA	NA
	N			NA	NA			NA	NA
1 Month FU	Mean								
	Std								
	N								
3 Months FU	Mean								
	Std								
	N								
6 Months FU	Mean		NA		NA		NA		NA
	Std		NA		NA		NA		NA
	N		NA		NA		NA		NA
12 Months FU	Mean		NA		NA		NA		NA
	Std		NA		NA		NA		NA
	N		NA		NA		NA		NA

Table 83 Additional Secondary Efficacy: Descriptive Statistics of QUEST Physical Score over Time by Treatment Group and Analysis Population (ITT, PP)

Visit / QUEST Physical Score		ITT				PP			
		Raw Values		Change from Baseline		Raw Values		Change from Baseline	
		ExAblate	Sham	ExAblate	Sham	ExAblate	Sham	ExAblate	Sham
Baseline	Mean			NA	NA			NA	NA
	Std			NA	NA			NA	NA
	N			NA	NA			NA	NA
1 Month FU	Mean								
	Std								
	N								
3	Mean								

Visit / QUEST Physical Score		ITT				PP			
		Raw Values		Change from Baseline		Raw Values		Change from Baseline	
		ExAblate	Sham	ExAblate	Sham	ExAblate	Sham	ExAblate	Sham
Months FU	Std								
	N								
6 Months FU	Mean		NA		NA		NA		NA
	Std		NA		NA		NA		NA
	N		NA		NA		NA		NA
12 Months FU	Mean		NA		NA		NA		NA
	Std		NA		NA		NA		NA
	N		NA		NA		NA		NA

Table 84 Additional Secondary Efficacy: Descriptive Statistics of QUEST Psychosocial Score over Time by Treatment Group and Analysis Population (ITT, PP)

Visit / QUEST Psychosocial Score		ITT				PP			
		Raw Values		Change from Baseline		Raw Values		Change from Baseline	
		ExAblate	Sham	ExAblate	Sham	ExAblate	Sham	ExAblate	Sham
Baseline	Mean			NA	NA			NA	NA
	Std			NA	NA			NA	NA
	N			NA	NA			NA	NA
1 Month FU	Mean								
	Std								
	N								
3 Months FU	Mean								
	Std								
	N								
6 Months FU	Mean		NA		NA		NA		NA
	Std		NA		NA		NA		NA
	N		NA		NA		NA		NA
12 Months FU	Mean		NA		NA		NA		NA
	Std		NA		NA		NA		NA
	N		NA		NA		NA		NA

Table 85 Additional Secondary Efficacy: Descriptive Statistics of QUEST Summary of Dimensions Total Score over Time by Treatment Group and Analysis Population (ITT, PP)

Visit / QUEST Summary of Dimensions Total Score		ITT				PP			
		Raw Values		Change from Baseline		Raw Values		Change from Baseline	
		ExAblate	Sham	ExAblate	Sham	ExAblate	Sham	ExAblate	Sham
Baseline	Mean			NA	NA			NA	NA
	Std			NA	NA			NA	NA
	N			NA	NA			NA	NA
1 Month FU	Mean								
	Std								
	N								
3 Months FU	Mean								
	Std								
	N								
6 Months FU	Mean		NA		NA		NA		NA
	Std		NA		NA		NA		NA
	N		NA		NA		NA		NA
12 Months FU	Mean		NA		NA		NA		NA
	Std		NA		NA		NA		NA
	N		NA		NA		NA		NA

Table 86 Covariate Analyses: Descriptive Statistics of Primary Endpoint (PE) by Covariate and Treatment Group (ITT)

Covariate / Category / PE			Treatment Group	
			ExAblate	Sham
Age	Age Subgroup 1	Mean		
		Std		
		Min		
		Median		
		Max		
		N		
	…	Mean		
		Std		
		Min		
		Median		
		Max		
		N		
Baseline CRST Score	CRST Subgroup 1	Mean		
		Std		
		Min		
		Median		
		Max		
		N		
	…	Mean		
		Std		
		Min		
		Median		
		Max		
		N		
Gender	Female	Mean		
		Std		
		Min		
		Median		
		Max		
		N		
	Male	Mean		
		Std		

Covariate / Category / PE			Treatment Group	
			ExAblate	Sham
		Min		
		Median		
		Max		
		N		
Center	Center 1	Mean		
		Std		
		Min		
		Median		
		Max		
		N		
	…	Mean		
		Std		
		Min		
		Median		
		Max		
		N		

Table 87 Covariate Analyses: Descriptive Statistics of First Secondary Endpoint (SE1) by Covariate and Treatment Group (ITT)

Covariate / Category / SE1			Treatment Group	
			ExAblate	Sham
Age	Age Subgroup 1	Mean		
		Std		
		Min		
		Median		
		Max		
		N		
	…	Mean		
		Std		
		Min		
		Median		
		Max		
		N		
Baseline CRST Score	CRST Subgroup 1	Mean		

Covariate / Category / SE1			Treatment Group	
			ExAblate	Sham
		Std		
		Min		
		Median		
		Max		
		N		
	…	Mean		
		Std		
		Min		
		Median		
		Max		
		N		
Gender	Female	Mean		
		Std		
		Min		
		Median		
		Max		
		N		
	Male	Mean		
		Std		
		Min		
		Median		
		Max		
		N		
Center	Center 1	Mean		
		Std		
		Min		
		Median		
		Max		
		N		
	…	Mean		
		Std		
		Min		
		Median		
		Max		

Covariate / Category / SE1			Treatment Group	
			ExAblate	Sham
		N		

Table 88 Covariate Analyses: Descriptive Statistics of Third Secondary Endpoint (SE3) by Covariate and Treatment Group (ITT)

Covariate / Category / SE3			Treatment Group	
			ExAblate	Sham
Age	Age Subgroup 1	Mean		
		Std		
		Min		
		Median		
		Max		
		N		
	…	Mean		
		Std		
		Min		
		Median		
		Max		
		N		
Baseline CRST Score	CRST Subgroup 1	Mean		
		Std		
		Min		
		Median		
		Max		
		N		
	…	Mean		
		Std		
		Min		
		Median		
		Max		
		N		
Gender	Female	Mean		
		Std		
		Min		
		Median		

Covariate / Category / SE3			Treatment Group	
			ExAblate	Sham
		Max		
		N		
	Male	Mean		
		Std		
		Min		
		Median		
		Max		
		N		
Center	Center 1	Mean		
		Std		
		Min		
		Median		
		Max		
		N		
	…	Mean		
		Std		
		Min		
		Median		
		Max		
		N		

Table 89 Covariate Analyses: P-values for Main Effect of Treatment, Covariate and Interaction Effects when Predicting PE, SE1 and SE3 (ITT)

Endpoint Predicted	Covariate Assessed	With or Without Interaction	P-Value, Group Effect	P-Value, Covariate Effect	P-Value Interaction
PE	Age	Without			
		With			
	Baseline CRST Score	Without			
		With			
	Gender	Without			
		With			
	Center	Without			
		With			

Endpoint Predicted	Covariate Assessed	With or Without Interaction	P-Value, Group Effect	P-Value, Covariate Effect	P-Value Interaction
SE1	Age	Without			
		With			
	Baseline CRST Score	Without			
		With			
	Gender	Without			
		With			
	Center	Without			
		With			
SE3	Age	Without			
		With			
	Baseline CRST Score	Without			
		With			
	Gender	Without			
		With			
	Center	Without			
		With			

Table 90 Covariate Analyses: Descriptive Statistics of Second Secondary Endpoint (SE2) by Covariate in ExAblate Group along with Comparison P-Value (ITT)

Covariate / Category / SE2			Treatment Group	P-Value
			ExAblate	
Age	Age Subgroup 1	Mean		
		Std		
		Min		
		Median		
		Max		
		N		
	…	Mean		
		Std		
		Min		
		Median		
		Max		
		N		
Baseline CRST Score	CRST Subgroup 1	Mean		
		Std		
		Min		
		Median		
		Max		
		N		
	…	Mean		
		Std		
		Min		
		Median		
		Max		
		N		
Gender	Female	Mean		
		Std		
		Min		
		Median		
		Max		
		N		
	Male	Mean		

Covariate / Category / SE2			Treatment Group	P-Value
			ExAblate	
		Std		
		Min		
		Median		
		Max		
		N		
Center	Center 1	Mean		
		Std		
		Min		
		Median		
		Max		
		N		
	…	Mean		
		Std		
		Min		
		Median		
		Max		
		N		

Figure 2 Covariate Analyses: Scatter Plot of SE2 versus Age (ITT)

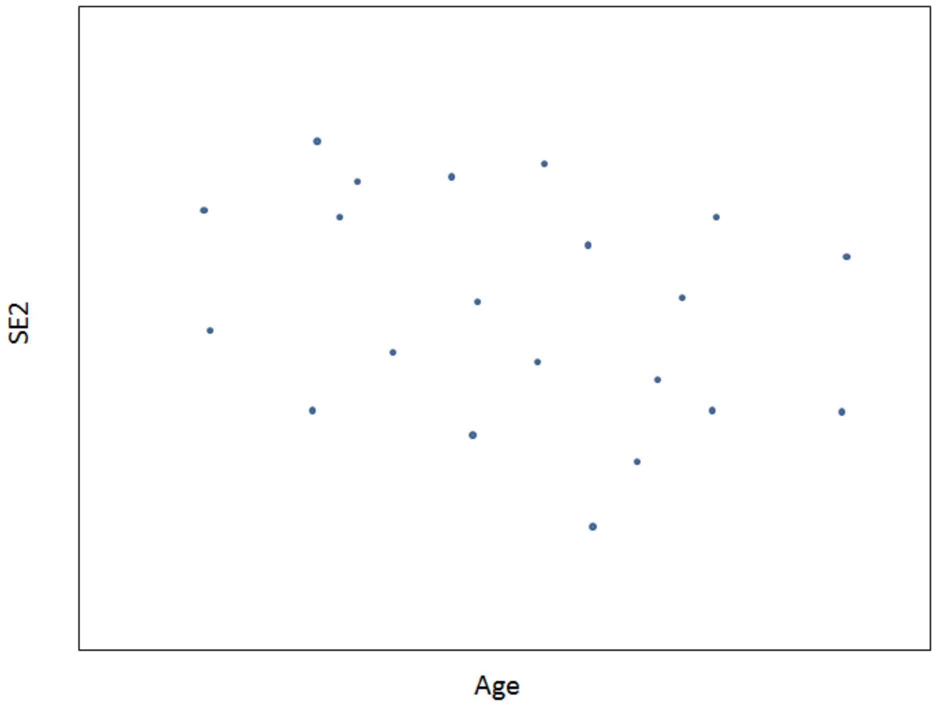

Figure 3 Covariate Analyses: Scatter Plot of SE2 versus Baseline CRST Score (ITT)

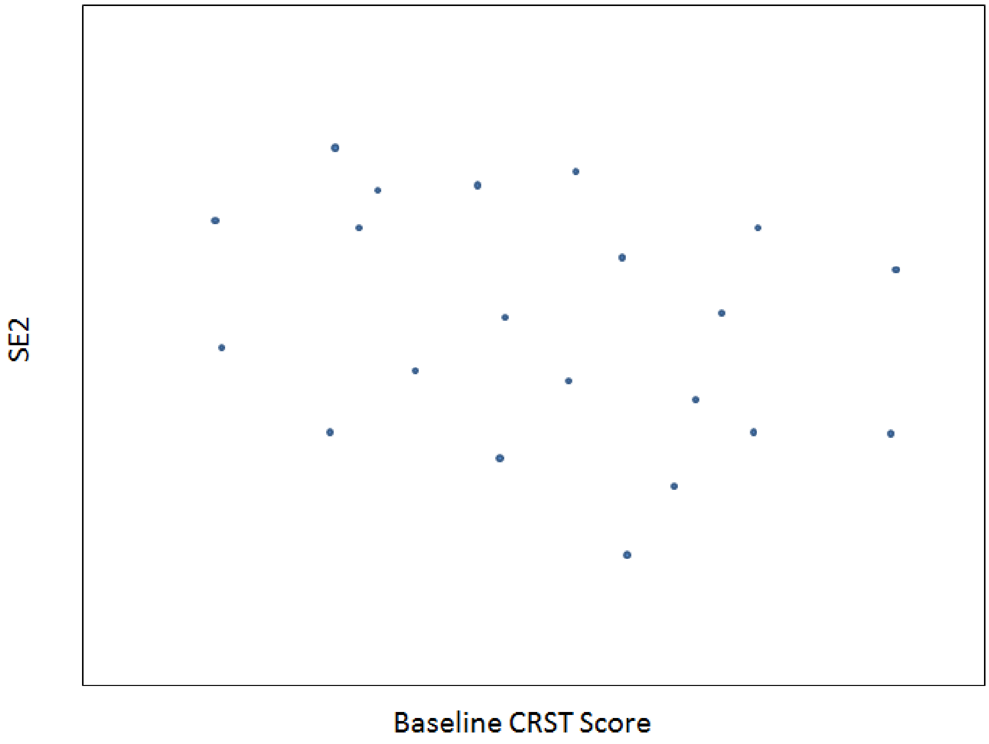

Table 91 Sensitivity Analyses: Worst Case – Descriptive Statistics of Percent Improvement from Baseline at Three Months Post-Treatment in the Treated (Contralateral) Upper Extremity CRST Sub Score by Treatment Group (ITT)

PE	Treatment Group		P-Value
	ExAblate	Sham	
Mean			
Std			
Min			
Median			
Max			
N			

Table 92 Sensitivity Analyses: Best Case – Descriptive Statistics of Percent Improvement from Baseline at Three Months Post-Treatment in the Treated (Contralateral) Upper Extremity CRST Sub Score by Treatment Group (ITT)

PE	Treatment Group		P-Value
	ExAblate	Sham	
Mean			
Std			
Min			
Median			
Max			
N			

Table 93 Sensitivity Analyses: Multiple Imputations – Descriptive Statistics of Percent Improvement from Baseline at Three Months Post-Treatment in the Treated (Contralateral) Upper Extremity CRST Sub Score by Treatment Group for Each of the 10 Imputed Data Sets (ITT)

Imputed Data Set / PE		Treatment Group		P-Value
		ExAblate	Sham	
1	Mean			
	Std			
	Min			
	Median			

Imputed Data Set / PE		Treatment Group		P-Value
		ExAblate	Sham	
	Max			
	N			
2	Mean			
	Std			
	Min			
	Median			
	Max			
	N			
3	Mean			
	Std			
	Min			
	Median			
	Max			
	N			
4	Mean			
	Std			
	Min			
	Median			
	Max			
	N			
5	Mean			
	Std			
	Min			
	Median			
	Max			
	N			
6	Mean			
	Std			
	Min			
	Median			
	Max			
	N			
7	Mean			
	Std			

Imputed Data Set / PE		Treatment Group		P-Value
		ExAblate	Sham	
	Min			
	Median			
	Max			
	N			
8	Mean			
	Std			
	Min			
	Median			
	Max			
	N			
9	Mean			
	Std			
	Min			
	Median			
	Max			
	N			
10	Mean			
	Std			
	Min			
	Median			
	Max			
	N			

Table 94 Sensitivity Analyses: Multiple Imputations – Overall Result (10 Imputed Datasets Combined) (ITT)

Treatment Group	Estimate	95% Lower CL	95% Upper CL
ExAblate			
Sham			
ExAblate - Sham			
